# In Silico Discovery and Optimisation of a Novel Structural Class of Hsp90 C-Terminal Domain Inhibitors

**DOI:** 10.3390/biom12070884

**Published:** 2022-06-24

**Authors:** Živa Zajec, Jaka Dernovšek, Martina Gobec, Tihomir Tomašič

**Affiliations:** Faculty of Pharmacy, University of Ljubljana, Aškerčeva Cesta 7, 1000 Ljubljana, Slovenia; ziva.zajec@ffa.uni-lj.si (Ž.Z.); jaka.dernovsek@ffa.uni-lj.si (J.D.); martina.gobec@ffa.uni-lj.si (M.G.)

**Keywords:** allosteric, cancer, heat shock, Hsp90, inhibitor, virtual screening

## Abstract

Hsp90 is a promising target for the development of novel agents for cancer treatment. The N-terminal Hsp90 inhibitors have several therapeutic limitations, the most important of which is the induction of heat shock response, which can be circumvented by targeting the allosteric binding site on the C-terminal domain (CTD) of Hsp90. In the absence of an Hsp90—CTD inhibitor co-crystal structure, the use of structure-based design approaches for the Hsp90 CTD is difficult and the structural diversity of Hsp90 CTD inhibitors is limited. In this study, we describe the discovery of a novel structural class of Hsp90 CTD inhibitors. A structure-based virtual screening was performed by docking a library of diverse compounds to the Hsp90β CTD binding site. Three selected virtual hits were tested in the MCF-7 breast cancer cell line, with compound **TVS-23** showing antiproliferative activity with an IC_50_ value of 26.4 ± 1.1 µM. We report here the optimisation, synthesis and biological evaluation of **TVS-23** analogues. Several analogues showed significantly enhanced antiproliferative activities in MCF-7 breast cancer and SK-N-MC Ewing sarcoma cell lines, with **7l** being the most potent (IC_50_ = 1.4 ± 0.4 µM MCF-7; IC_50_ = 2.8 ± 0.4 µM SK-N-MC). The results of this study highlight the use of virtual screening to expand the structural diversity of Hsp90 CTD inhibitors and provide new starting points for further development.

## 1. Introduction

Hsp90, one of the most abundant proteins in the cell cytoplasm [1], is a molecular chaperone responsible for maintaining and regulating protein homeostasis by facilitating protein folding and maturation, mediating refolding of misfolded proteins and preventing protein aggregation [2]. Hsp90 is overexpressed in many cancers where it plays an important role in promoting carcinogenesis by correctly folding oncogenic client proteins (e.g., c-Raf, Her2, Akt, HIF1, CDK) involved in tumour growth and adhesion, metastasis, neoangiogenesis, invasion and apoptosis [3,4,5,6]. In addition, Hsp90 protects cancer cells from hypoxia, genetic instability and proteotoxic and nutritional stress induced by malignant transformation [7]. Because Hsp90 plays a critical role in cancer, it has become an attractive target for anticancer drug development. Inhibition of Hsp90 has the great advantage of subsequently affecting multiple oncogenic proteins and signalling pathways involved in malignant transformation [8].

Hsp90 is a homodimer; dimerisation of two monomers is essential for its function. Each monomeric unit consists of the following three domains: the N-terminal domain (NTD) responsible for ATPase activity, the middle domain (MTD) important for interaction with co-chaperones and the C-terminal domain (CTD) responsible for dimerisation of Hsp90 [9,10,11]. Hsp90 consists of four different isoforms; the cytosolic isoforms Hsp90α and Hsp90β, the mitochondrial isoform TRAP1, and Grp94, which is localised in the endoplasmic reticulum [12].

The first Hsp90 inhibitor identified was the natural product geldanamycin [13], which binds to the Hsp90 NTD. Since then, many analogues of geldanamycin, such as 17-AAG and 17-DMAG, as well as other Hsp90 NTD inhibitors, have been developed, several of which have entered clinical trials [14,15]. Unfortunately, none of them has been successful so far due to toxicities (hepatotoxicity, cardiotoxicity, ocular side effects) and induction of heat shock response (HSR). HSR induces upregulation of the pro-survival and anti-apoptotic heat shock proteins Hsp70 and Hsp27, which significantly reduces the efficacy of Hsp90 NTD inhibitors [16,17,18,19,20]. Therefore, alternative strategies to inhibit Hsp90 are required, such as isoform-selective Hsp90 inhibition, targeting protein-protein interactions between Hsp90 and its co-chaperones and client proteins and allosteric Hsp90 CTD inhibition [21,22,23].

**Figure 1:**
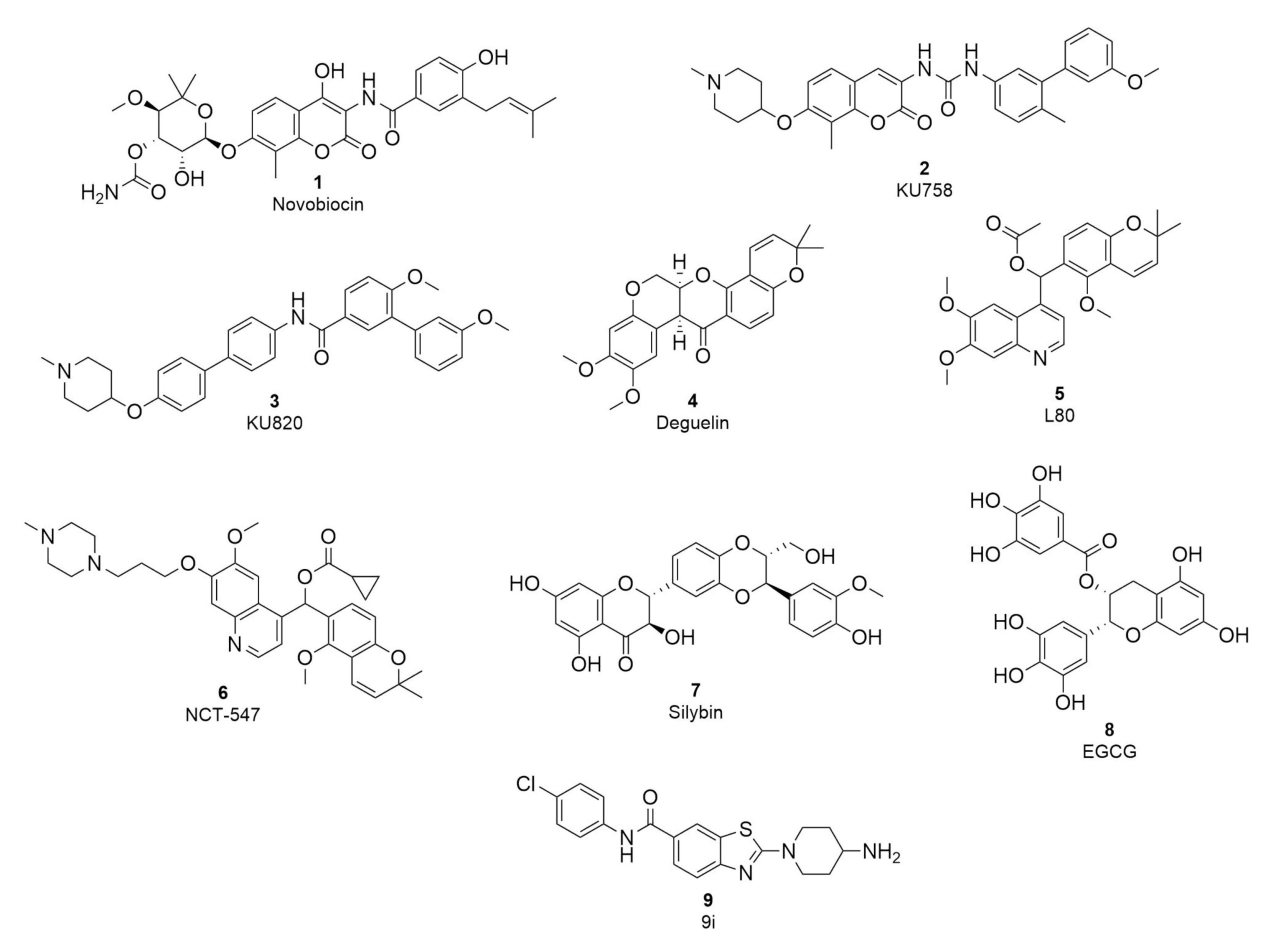
Representative structures of Hsp90 CTD inhibitors; novobiocin and analogues (**1–3**) [28,29], deguelin and analogues (**4–6**) [31,33], silybin (**7**), EGCG (**8**) and a benzothiazole-based inhibitor (**9**) [37].

The first Hsp90 CTD inhibitor discovered was the coumarin antibiotic novobiocin (**1**, Figure 1), which binds to the allosteric nucleotide-binding site in the CTD that becomes available only after ATP binds to the NTD [24,25]. Novobiocin has a modest antiproliferative effect but nevertheless lowers the level of oncogenic proteins without inducing HSR [24,26]. In an attempt to discover more potent Hsp90 CTD inhibitors and explore their structure-activity relationships (SAR), several novobiocin analogues were synthesised [27]. Studies of the novobiocin analogues showed that noviose sugar can be replaced with ionisable amines, which later proved to be important for improved activity against cancer cells. In addition, the coumarin core can be substituted for a biaryl or phenyl cyclohexyl carboxamide fragment (**2** and **3**, Figure 1) [28,29,30]. Although the potency of Hsp90 CTD inhibitors has improved significantly compared to novobiocin, the scaffold diversity is still scarce. Known inhibitors include novobiocin analogues, analogues of other natural products such as deguelin, EGCG, silybin and scaffolds already established as inhibitors of other targets, e.g., benzothiazole-based DNA gyrase B inhibitors (**4–9**, Figure 1) [31,32,33,34,35,36,37]. To expand the structural diversity of Hsp90 CTD inhibitors, we performed structure-based virtual screening to discover novel scaffolds.

The structures of the full-length Hsp90 dimer have been determined by X-ray crystallography and more recently by cryo-electron microscopy [38,39]. However, the structure of Hsp90 in complex with a non-covalent allosteric CTD inhibitor has not yet been determined, making structure-based design challenging. Recently, we developed ligand-based and molecular dynamics (MD)-derived structure-based pharmacophore models for Hsp90 CTD inhibitor design and the identification of new hits by virtual screening [40]. We discovered a new structural class of Hsp90 CTD inhibitors bearing a substituted aromatic ring and a basic amine at the required distance based on the observed structure-activity relationships and our pharmacophore models. Moreover, we have successfully used the pharmacophore model to optimise the benzothiazole class of Hsp90 CTD inhibitors [37].

Computational techniques have been used previously to identify novel Hsp90 CTD inhibitors, but they were mainly based on ligand-based methods [41,42]. To the best of our knowledge, structure-based virtual screening has not been used to identify Hsp90 CTD inhibitors. With the goal of discovering new starting points for the development of Hsp90 CTD inhibitors for cancer treatment, a library of diverse commercially available compounds was docked to the Hsp90β CTD binding site in the conformation from the MD simulation trajectory, from which we had previously derived our structure-based pharmacophore model. We report here the discovery of a new chemotype of Hsp90 CTD inhibitors, the synthesis of analogues, molecular modelling studies and the antiproliferative activity of the compounds in cancer cell lines.

## 2. Materials and Methods

### 2.1. Chemistry

Reagents and solvents for synthesis were purchased from Enamine Ltd. (Kyiv, Ukraine), Fluorochem Ltd. (Hadfield, Derbyshire, UK), Sigma-Aldrich (St. Louis, MO, USA) and TCI (Tokyo, Japan), and were not further purified. Analytical thin-layer chromatography was performed on silica gel aluminium sheets (0.20 mm; 60 F254; Merck, Darmstadt, Germany). Reverse phase flash chromatography was carried out on Biotage^®^ Isolera One system (Biotage, Uppsala, Sweden) using Biotage^®^ Sfär Bio C18 Duo 300 Å 20 μm column. Mobile phase consisted of 0.1% CF_3_COOH in purified water (solvent A) and acetonitrile (solvent B). Gradient used was 100% solvent A to 100% solvent B in 410 mL. The ^1^H and ^13^C NMR spectra were recorded on a 400 MHz NMR spectrometer (Bruker Advance 3, Bruker, Billerica, MA, USA). The purities of the prepared compounds were monitored by liquid chromatography-mass spectrometry that was performed using method A (see below) on a 1260 Infinity II LC system (Agilent Technologies, Santa Clara, CA, USA), which was equipped with a quaternary pump and a wavelength detector. The system was coupled to mass spectrometry (Expression CMS^L^; Advion Inc., Ithaca, NY, USA). The high-resolution mass spectra (HRMS) were recorded on Exactive Plus Orbitrap mass spectrometer (Thermo Scientific Inc., Waltham, MA, USA).

Method A: A C18 column was used (Waters xBridge BEH; 4.6 mm × 150 mm, 3.5 µm) at 40 °C. The flow rate of the mobile phase was 1.5 mL/min, the injection volume was 10 µL, and the products were detected at 254 nm. Solvent A comprised 1% CH_3_CN and 0.1% HCOOH in double-distilled H_2_O; Solvent B comprised CH_3_CN. The following elution gradient was used: 0→1 min, 25% B; 1→6 min, 25%→98% B; 6→6.5 min, 98% B; 6.5→7 min, 98%→25% B; 7→10 min, 25% B.

Chemical synthesis procedures and analytical data of all intermediates and final compounds are described in Appendix A.

### 2.2. Virtual Screening

#### 2.2.1. Preparation of the Compound Library

Diversity sets of small molecule libraries from Asinex, ChemBridge, Enamine, Life chemicals, Key Organics, Maybridge, Vitas-M and Pharmeks were downloaded from vendor websites in SDF format. These libraries were merged and duplicates removed, which resulted in a library containing 2,081,456 compounds. For these compounds a library of conformers was generated using OMEGA software (Release 2.5.1.4, OpenEye Scientific Software, Inc., Santa Fe, NM, USA; www.eyesopen.com (accessed on 20 June 2020)) [43] using default settings, which resulted in a maximum of 200 conformers per ligand.

#### 2.2.2. Virtual Screening

The Hsp90 CTD binding site (PDB entry: 5FWK) was created using MAKE RECEPTOR (Release 3.2.0.2, OpenEye Scientific Software, Inc., Santa Fe, NM, USA; www.eyesopen.com). The grid box (dimensions: 21.7 Å × 24. 7 Å × 16.0 Å; volume 8551 Å^3^) was automatically generated around the docked Hsp90 CTD inhibitor [40] and was not adjusted. For “cavity detection”, the “molecular” method was used. The outer and inner contours were automatically calculated with the “Balanced” settings; however, the inner contours were disabled. The OMEGA library of commercially available compounds was then rigidly docked to the prepared Hsp90 CTD binding site using FRED (OEDOCKING 3.3.0.2: OpenEye Scientific Software, Santa Fe, NM, USA. http://www.eyesopen.com (accessed on 20 June 2020)) [44,45] with the default settings. Docking poses (10 per compound) were scored and ranked using Chemgauss4 scoring function. The results were visualised and analysed with VIDA (version 4.3.0.4, OpenEye Scientific Software, Inc., Santa Fe, NM, USA; www.eyesopen.com (accessed on 20 June 2020)).

### 2.3. Molecular Dynamics Simulations

MD simulations of the Hsp90-**TVS23** and Hsp90-**7l** complexes were performed using NAMD package (version 2.9) [46] and the CHARMM36m [47] force field. Molecular mechanics parameters for compounds **TVS23** and **7l** were estimated using the ParamChem tool [48,49,50]. Removal of potential steric clashes and optimisation of the atomic coordinates of the Hsp90β-**TVS23** and Hsp90-**7l** docking complexes were first performed by steepest descent (10,000 steps) and adopted basis Newton-Raphson (10,000 steps) energy minimisations. The systems for MD simulation were prepared using psfgen in VMD (version 1.9.1.) [51]. Structures of the Hsp90-**TVS23** and Hsp90-**7l** complexes were first embedded in a box of TIP3P water molecules. Then the system was neutralised by addition of NaCl. The MD simulation was run in the NPT ensemble using the periodic boundary conditions. Temperature (300 K) and pressure (1 atm) were controlled using the Langevin dynamics and Langevin piston methods, respectively. Short-range and long-range forces were calculated every 1 and 2 timesteps, respectively, with a time step of 2.0 ps. The smooth particle mesh Ewald method was used to calculate the electrostatic interactions [52]. The short-range interactions were cut off at 12 Å. All of the chemical bonds between hydrogen and the heavy atoms were held fixed using the SHAKE algorithm [53]. The simulation consisted of the following three consecutive steps: (i) solvent equilibration for 1 ns with ligand and protein constrained harmonically around the initial structure; (ii) equilibration of the complete system for 1 ns with ligand and protein released; (iii) an unconstrained 1000 ns production run. For structure-based pharmacophore modelling, 5000 frames from the production run were saved separately and used for interaction analysis.

### 2.4. Structure-Based Pharmacophore Modeling

The 1000 ns MD trajectory of Hsp90β dimer (PDB Entry: 5FWK) in complex with compound **TVS23** or **7l** was used for pharmacophore feature analysis using LigandScout 4.4 Expert, which resulted in 5000 structure-based pharmacophore models.

### 2.5. MTS Assay

The compounds were evaluated for their antiproliferative activity against the MCF - 7 (ATCC HTB -22) breast cancer cell line and SK-N-MC Ewing sarcoma cell line (cells were a gift from Beat Schäfer) using an MTS (Promega, Madison, WI, USA) assay according to the manufacturer’s instructions. Independent experiments were repeated twice and performed in triplicate each time. Statistically significant differences (*p* < 0.05) between treated groups and DMSO were calculated using two-tailed Welch’s *t*-tests. IC_50_ values were determined using GraphPad Prism 9.1 software (San Diego, CA, USA) and represent the concentration at which an agent elicits a half-maximal response; they are expressed as the mean values of the independent measurements. Further details can be found in the Supporting Information.

#### Luciferase Refolding Assay

PC3MM2*luc* cells (cells were a gift from Brian Blagg) were cultured in Dulbecco’s modified Eagle’s medium, high glucose (Gibco, Thermo Fisher Scientific, Waltham, MA, USA) supplemented with 5 µg/mL puromycin (InvivoGen, San Diego, CA, USA), 100 U/mL penicillin (Sigma-Aldrich, St. Louis, MO, USA) and 100 µg/mL streptomycin (Sigma-Aldrich, St. Louis, MO, USA) and 10% foetal bovine serum (Gibco, Thermo Fisher Scientific, Waltham, MA, USA) at 37 °C and under 5% CO_2_. Cell pellets were suspended in prewarmed medium (50 °C) for 2 min to induce firefly luciferase unfolding. The cells were plated in 96-well plates at a density of 50.000 cells per well in the presence of selected compound or vehicle control (1% DMSO). The plates were incubated for 60 min at 37 °C to allow for luciferase refolding. After incubation, 100 µL of ONE-Glo™ Luciferase Assay System (Promega, Madison, WI, USA) was added to each well of the plate and incubated for another 5 min. Luciferase activity was determined by measuring luminescence with BioTek’s Synergy™ 4 Hybrid Microplate Reader (Winooski, VT, USA). Luciferase activity was calculated as a percentage relative to vehicle control.

## 3. Results and Discussion

### 3.1. Virtual Screening

**Figure 2:**
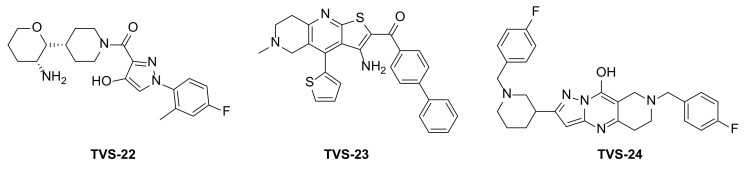
Virtual screening hits tested for antiproliferative activity in cancer cell lines.

In the absence of an experimental structure of Hsp90 in a complex with a CTD inhibitor, we used our recently published structure of the Hsp90β-coumarin-based inhibitor complex, obtained by a combination of molecular docking, MD simulation and structure-based pharmacophore modelling [40]. Specifically, the conformation of the complex used in the virtual screening experiment was derived from the MD trajectory time point from which the most commonly appearing structure-based pharmacophore model for the Hsp90 CTD inhibitor was obtained. A library of diverse commercially available compounds obtained from various vendors was docked in the Hsp90β CTD binding site using FRED (OEDOCKING 4.1.1.0: OpenEye Scientific Software, Inc., Santa Fe, NM. http://www.eyesopen.com (accessed on 20 June 2020)). Virtual screening hits were scored using the Chemgauss4 scoring function and the hundred highest-ranked compounds were further analysed (Chemgauss4 scores ranging from −14.491 to −13.0205). In the visual inspection of the predicted binding modes, a basic centre interacting with the Glu489A side chain and a hydrophobic moiety at the other end of the compound were considered a prerequisite for Hsp90 CTD binding based on our previously reported pharmacophore models. Virtual screening hits for biological evaluation (Figure 2) were selected based on the Chemgauss4 scoring function score and predicted binding mode.

**Figure 3:**
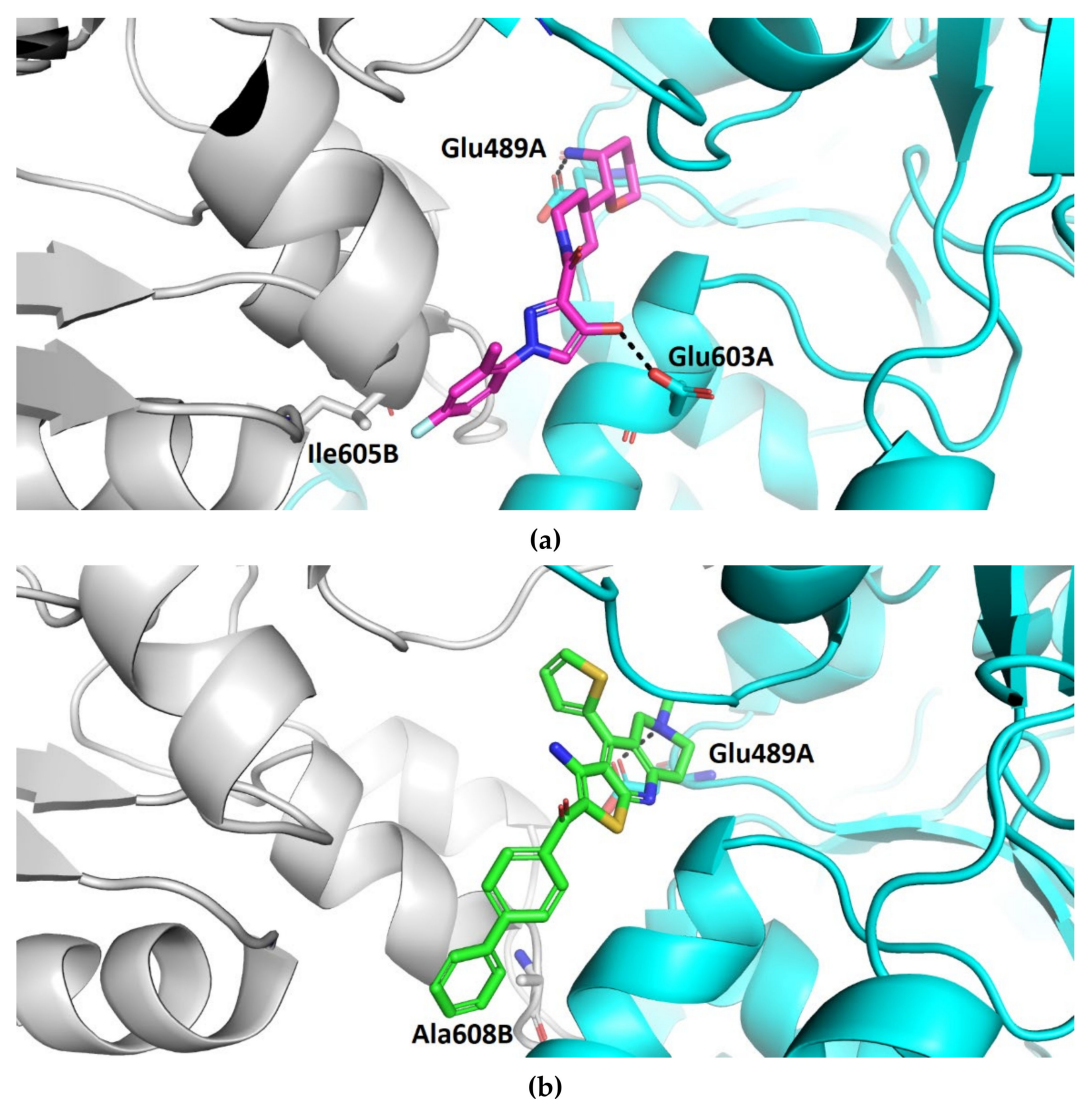

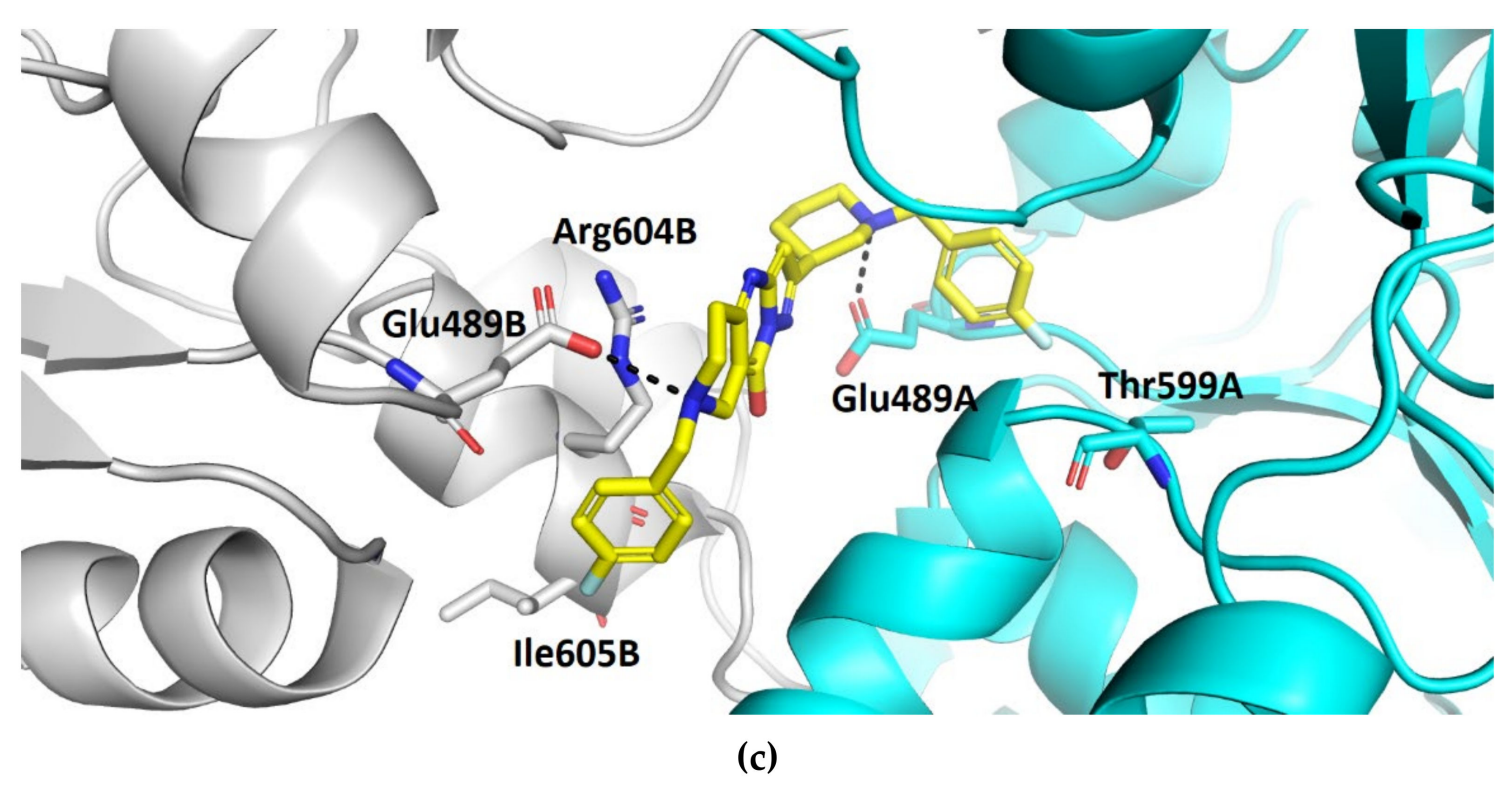
Binding modes of (**a**) **TVS-22** (in magenta sticks), (**b**) **TVS-23** (in green sticks) and (**c**) **TVS-24** (in yellow sticks) in the C-terminal domain binding site of Hsp90β dimer (protomers A and B are coloured cyan and grey, respectively). For clarity, only amino acids forming hydrogen bonds (black dashed lines), cation-π and hydrophobic interactions with inhibitors are shown as sticks.

Compound **TVS-22** (Chemgauss4 score: −13.0805) was predicted to form a hydrogen bond and ionic interaction between the amino group at position 3 of the oxane ring, and the Glu489A side chain, an additional hydrogen bond between the hydroxy group at position 3 of the pyrazole ring and the Glu603A side chain and hydrophobic interactions between the 4-fluorophenyl moiety and the Leu605B side chain (Figure 3a). Similarly, the basic amine of **TVS-23** (Chemgauss4 score: −13.4070) interacted with Glu489A and formed additional hydrophobic contacts between the biphenyl moiety and Ala608B (Figure 3b). The most extensive network of interactions was formed between the compound **TVS-24** (Chemgauss4 score: −14.1372) and the Hsp90 binding site residues. In detail, the piperidine rings of **TVS-24** formed hydrogen bonds and ionic interactions with Glu489A and Glu489B, while one of the 4-fluorophenyl rings formed hydrophobic interactions with Ile605B and the one on the opposite side of the molecule with Thr599A. The pyrazolo [1,5-*a*]pyrimidin-7-ol ring of the central scaffold formed an additional cation-π interaction with the guanidinium group of the Arg604B side chain (Figure 3c).

Compounds **TVS-22**, **TVS-23** and **TVS-24** were tested for their antiproliferative activity in the breast cancer cell line MCF-7 using the MTS assay. **TVS-23** and **TVS-24** showed activity in the micromolar range with IC_50_ values of 26.4 ± 1.1 µM and 47.9 ± 0.9, respectively, while **TVS-22** was found to be inactive at 50 µM. The optimisation strategy and synthesis of **TVS-23** analogues are presented below, while the optimisation of **TVS-24** will be described elsewhere.

### 3.2. Molecular Dynamics Simulation

**Figure 4:**
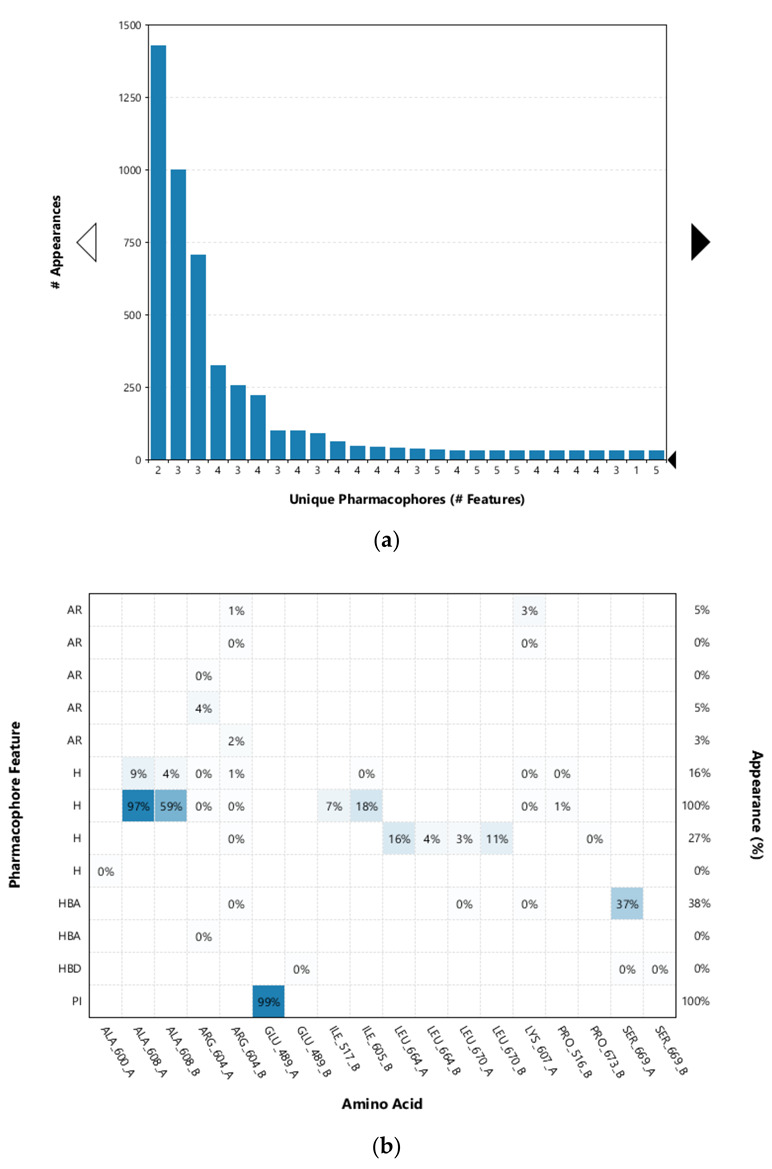
(**a**) Plot of the most frequent unique structure-based pharmacophore models derived from the molecular dynamics simulations of the Hsp90 C-terminal domain in complex with **TVS-23**. The numbers below the bars indicate the numbers of interaction features observed during molecular dynamics simulation for the pharmacophore models. (**b**) MD Interaction map obtained by analysing interactions of **TVS-23** with Hsp90 CTD binding site residues in the 1 µs MD simulation trajectory. Amino acid name and numbering is shown on *x*-axis, pharmacophore feature type on the left *y*-axis (H—hydrophobic, HBA—hydrogen bond acceptor, HBD—hydrogen bond donor, PI—positive ionizable, AR—aromatic), % appearance on the right *y*-axis.

**Figure 5:**
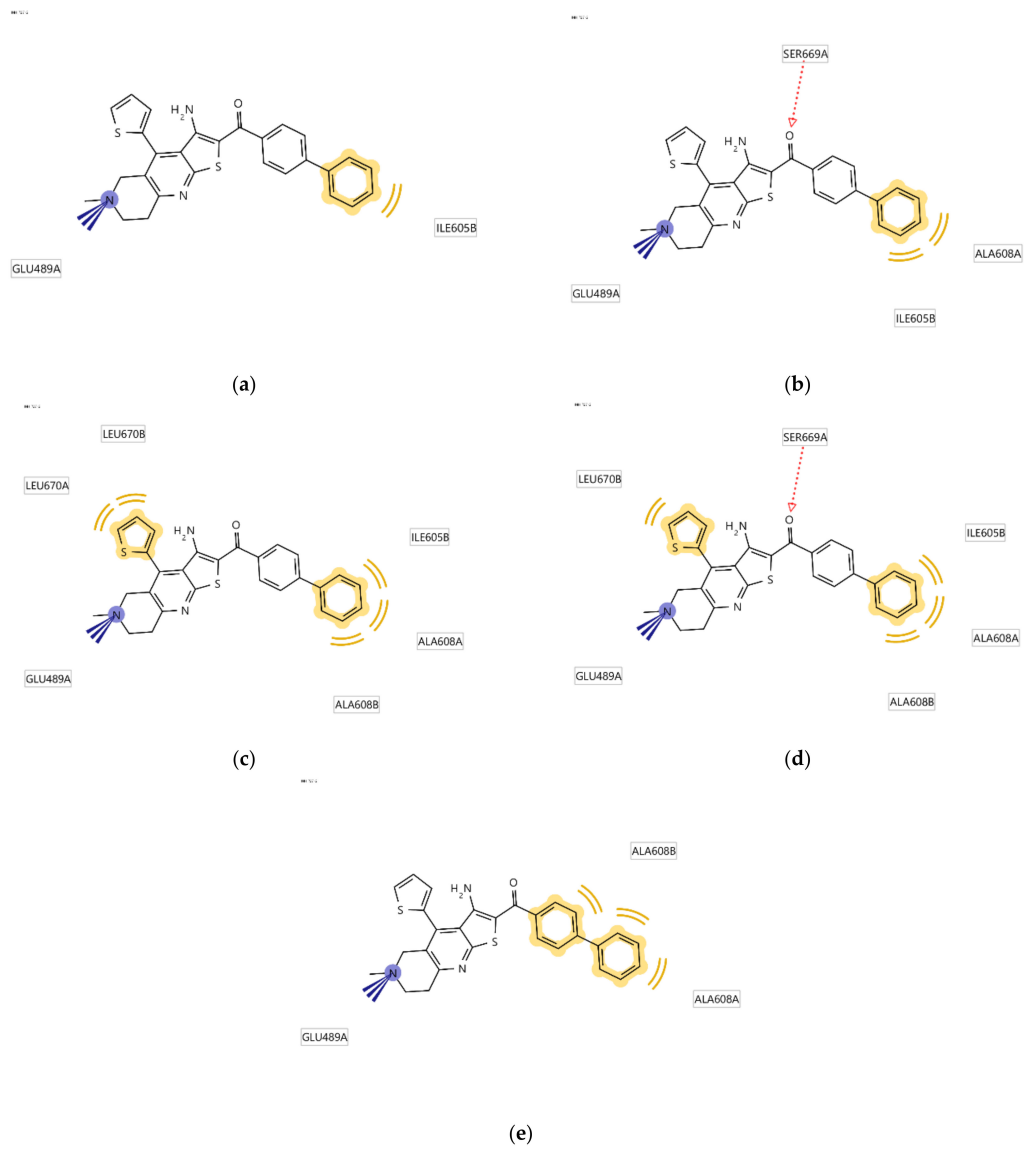
Schematic representation of the five most frequently appearing pharmacophore models during the MD trajectory (**a**–**e**).

Since the Hsp90 dimer is quite flexible and there is no crystal structure of the Hsp90-CTD inhibitor complex, the binding mode of **TVS-23** was further investigated using molecular dynamics simulation. The docking complex (Figure 3b) was used as a starting point for a 1 µs MD simulation. The interaction features between the allosteric Hsp90 CTD binding site residues and **TVS-23** during the MD trajectory (5000 frames) were analysed using the MD analysis tool in LigandScout 4.4 Expert. Figure 4a shows the plot of the most frequently occurring unique structure-based pharmacophore models versus the number of appearances. The most frequent model was seen more than 1400 times and has interactions consistent with those observed in the docking binding mode (Figure 3b and Figure 5a). The next four most frequent models, each appearing more than 250 times (Figure 4a), exhibit some additional pharmacophore features (Figure 5b–e), including hydrogen bonds between the carbonyl group and the Ser669A side chain, and additional hydrophobic interactions that highlight the importance of the thiophene and biphenyl structural elements. Analysis of the interactions reveals a permanently present ionic interaction between the piperidine ring of **TVS-23** and the Glu489A side chain (99% of simulation time). Other important interactions include hydrophobic contacts with Ala608A and Ala608B and a hydrogen bond with Ser669A (37% of simulation time) (Figure 4b).

### 3.3. Design and Synthesis

**Figure 6:**
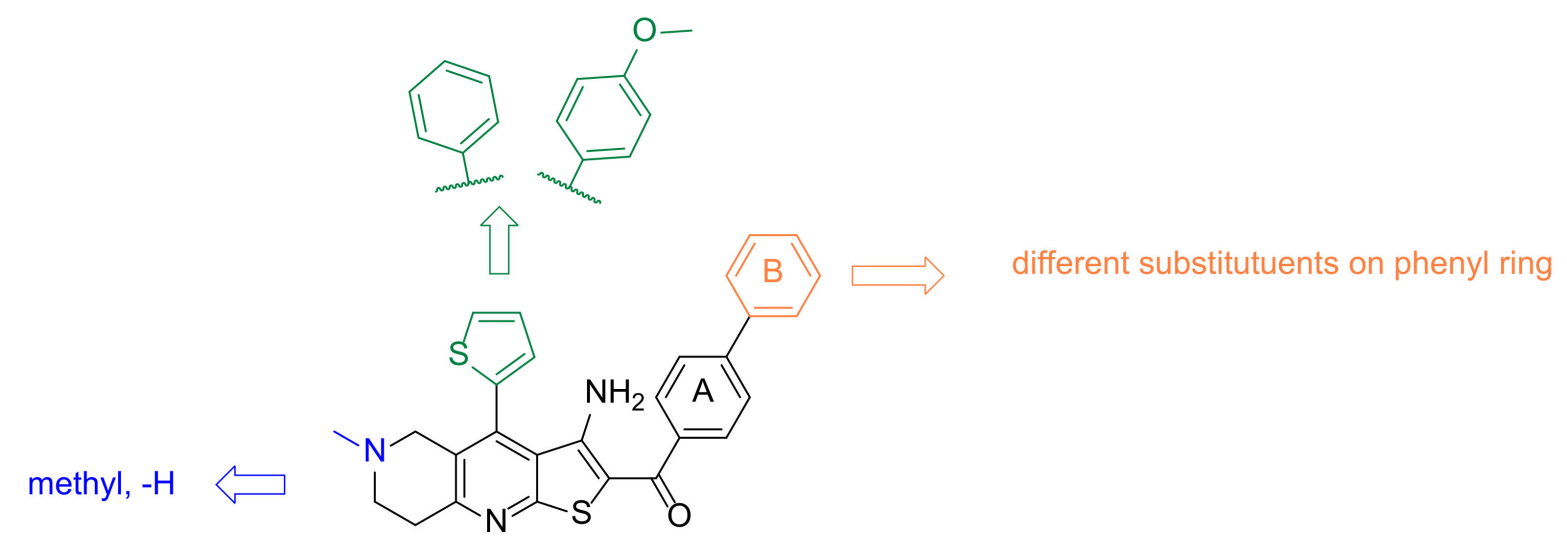
Design strategy for optimisation of compound **TVS-23**.

To further investigate the interaction features and optimise the screening hit **TVS-23**, several analogues were synthesised. The optimisation strategy is shown in Figure 6.

**Scheme 1:**
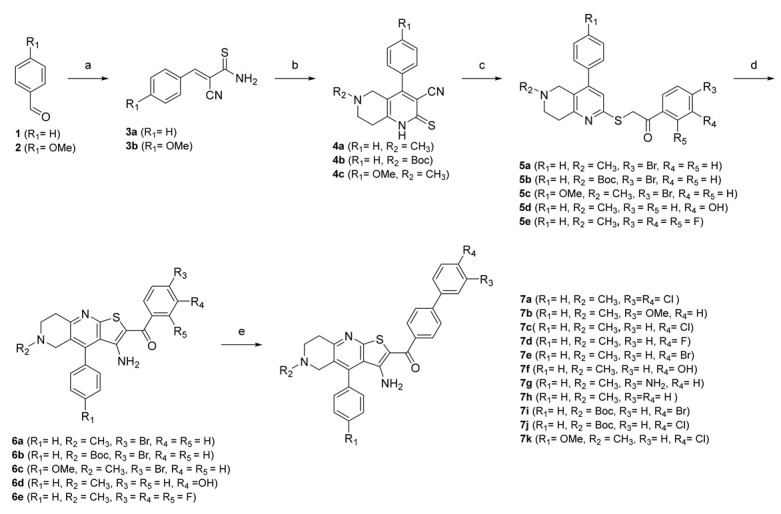
Reagents and conditions: (a) 2-cyanothioacetamide, triethylamine, absolute EtOH, 50 °C, 1 h; (b) for **4a** and **4c**: *N*-methylpiperidin-4-one, for **4b** *tert*-butyl 4-oxopiperidine-1-carboxylate, piperidine, EtOH, reflux, 2 h; (c) for **5a**–**c**: 2,4′-dibromoacetophenone, for **5d**: 2-bromo-3′-hydroxyacetophenone, for **5e**: 2-bromo-2′,3′,4′-trifluoroacetophenone, sodium acetate, EtOH, reflux, 1 h; (d) sodium methoxide, absolute EtOH, reflux, 30 min, (e) for **7a**: (3,4-dichlorophenyl)boronic acid, for **7b**: (3-methoxyphenyl)boronic acid), for **7c, 7j**–**k**: (4-chlorophenyl)boronic acid, for **7d**: (4-fluorophenyl)boronic acid, for **7e, 7i**: (4-bromophenyl)boronic acid, for **7f**: (4-hydroxyphenyl)boronic acid, for **7g**: (3-aminophenyl)boronic acid, for **7h**: phenylboronic acid, tetrakis(triphenylphosphine)palladium(0); K_2_CO_3_, mixture of water and 1,4-dioxane, reflux, overnight.

First, we replaced the thiophene ring with a phenyl ring (**7a**–**j**, Scheme 1), which provides similar hydrophobic interactions to those shown to be important in two of the most common pharmacophore models (Figure 5c,d). To further investigate these interactions, we synthesised an analogue with a substituted phenyl ring, which can also form hydrogen bonds (**7k**, Scheme 1). One of the most important interactions between **TVS-23** and the allosteric Hsp90 CTD binding site revealed in our MD simulation is an ionic interaction between the piperidine ring and Glu489A. Therefore, we retained this basic centre in all of our synthesised analogues and replaced the tertiary amine with a secondary one in some analogues. We have synthesised analogues with various substituents and different substitution patterns on phenyl ring B, which is important for hydrophobic interactions with Ala608A and Ala608B. Analogues of **TVS-23** were synthesised as shown in Scheme 1.

**Scheme 2:**
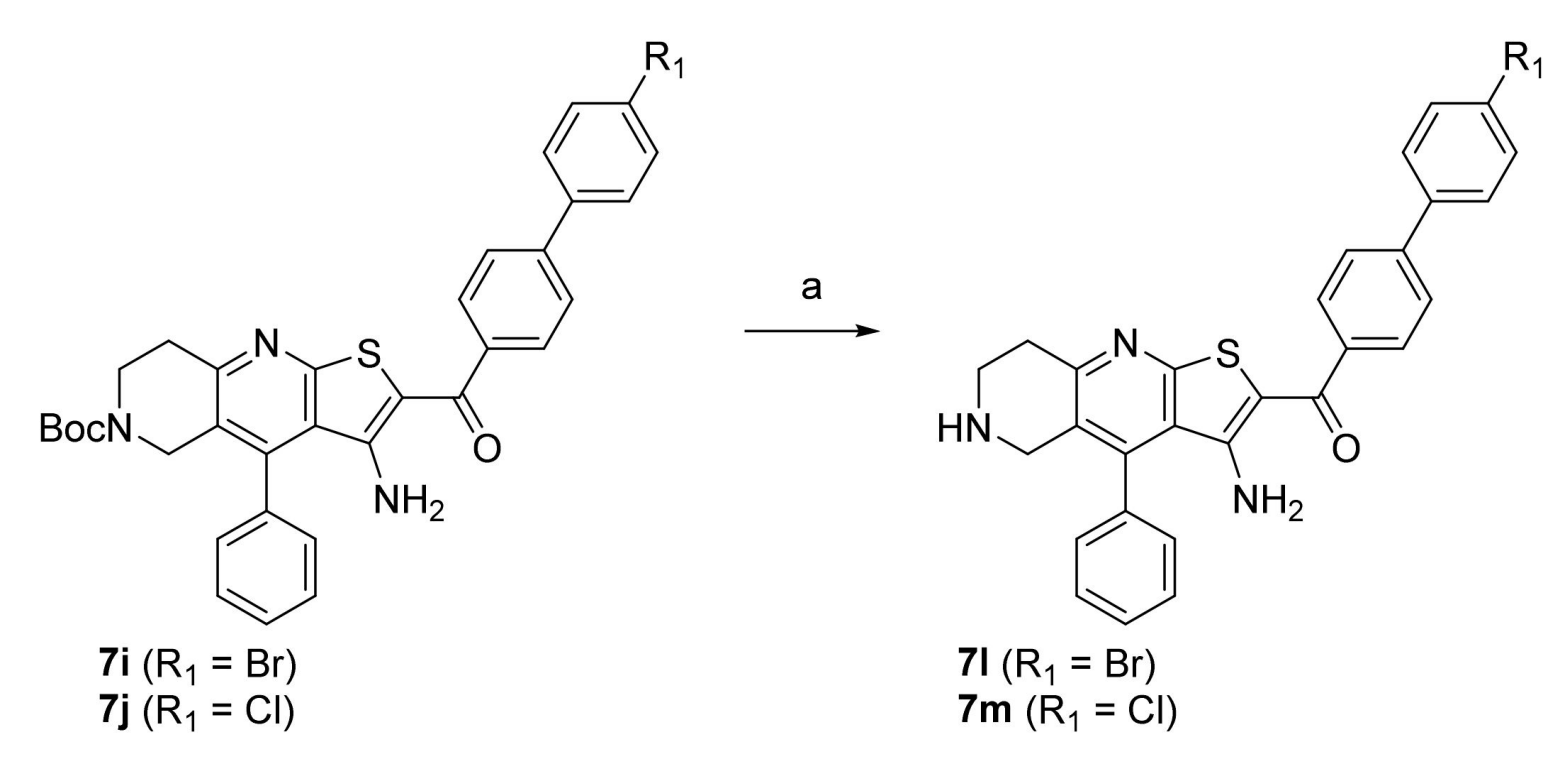
Reagents and conditions: (a) CF_3_COOH, dichloromethane, r.t., 72 h.

In the first step, a reaction between benzaldehydes (**1** or **2**) and 2-cyanothioacetamide in absolute ethanol gave benzylidenecyanothioacetamide (**3a**–**b**), followed by a reaction with piperidin-4-ones in the presence of a few drops of piperidine, which yielded 3-cyanopyridin-2(1*H*)-thiones (**4a**–**c**). In the next step, S-alkylation was carried out with bromoacetophenones to obtain intermediates **5a**–**e**, which undergo cyclisation in the presence of sodium methoxide to give thienopyridine heterocycles **6a**–**e**. In the last step, a Suzuki coupling was carried out with differently substituted phenylboronic acids to prepare the final compounds **7a**–**k**. The same steps were applied to the synthesis of Boc-protected compounds **7i** and **7j**, which were deprotected by acidolysis to prepare the final compounds **7l** and **7m** (Scheme 2).

### 3.4. Biological Evaluation

All final compounds were evaluated for their antiproliferative activity against the MCF-7 breast cancer line and the SK-N-MC Ewing sarcoma cell line using the MTS assay. Both cell lines were validated for their overexpression of Hsp90 using Western blot (Supporting Information, Figure S30). The results presented in Table 1 confirm the importance of phenyl ring B as a key feature for activity. Compounds **6e** and **6d**, which lack this structural element, were found to be inactive against both cancer cell lines (Table 1).

Replacing the thiophene with a phenyl ring preserved the antiproliferative effect against both cell lines, as the IC_50_ of compound **7h** is very similar to that of **TVS-23** (Table 2). Next, we examined the effects of phenyl ring B substitutions on the antiproliferative activity of compounds **7a**–**h** (Table 2). The results show that additional hydrophobic substituents at the *para* position of phenyl ring B, such as chlorine (**7c**), increase the antiproliferative activity, possibly due to stronger hydrophobic interactions with Ala608 in the proposed Hsp90 CTD binding site. We also explored the possibility of a halogen bond and therefore introduced fluorine (**7d**) and bromine (**7e**) at the *para* position of the phenyl ring B. The activities of compounds **7d**, **7c** and **7e** against both cancer cell lines are increasing, respectively, which may indicate that a halogen bond is present in addition to the hydrophobic interaction. An additional chlorine substituent, compound **7a**, at the *meta* position of the phenyl ring B lowered the activity compared to **7c**. The introduction of polar substituents such as hydroxyl (**7f**) or methoxy (**7b)** had a very limited effect on the activity compared with **TVS-23**. Interestingly, the introduction of a polar amino group (**7g**) at the *meta* position of the ring slightly improved the antiproliferative activity compared with **TVS-23**.

The two most potent compounds, **7c** and **7e,** were selected for further investigation of SAR. First, analogues of these two compounds without an *N*-methyl group on the piperidine ring were synthesised and tested. The results presented in Table 3 show, that removal of the *N*-methyl group resulted in more potent compounds **7l** and **7m** with respect to **7e** and **7c.** The introduction of a polar methoxy group on the phenyl ring (**7k**) has significantly decreased the potency compared to the analogue **7c**. Interestingly, the decrease in activity was much more pronounced in the MCF-7 breast cancer line.

To confirm that the antiproliferative effect was due to inhibition of Hsp90, a luciferase refolding assay was performed [54]. Representative compound **7l** inhibited Hsp90-dependent luciferase refolding (Supporting information, Figure S31), demonstrating that analogues of the virtual hit **TVS-23** inhibit Hsp90. Next, we performed an *in silico* screening using a set of ligand-based pharmacophore models for the identification of Hsp90 NTD inhibitors [55], which resulted in no hits. Because the observed SAR is consistent with the hypothesis for the binding to the proposed Hsp90 CTD binding site and **TVS-23** and its analogues do not have the necessary features of Hsp90 NTD inhibitors, we believe that they bind to the Hsp90 CTD.

**Figure 7:**
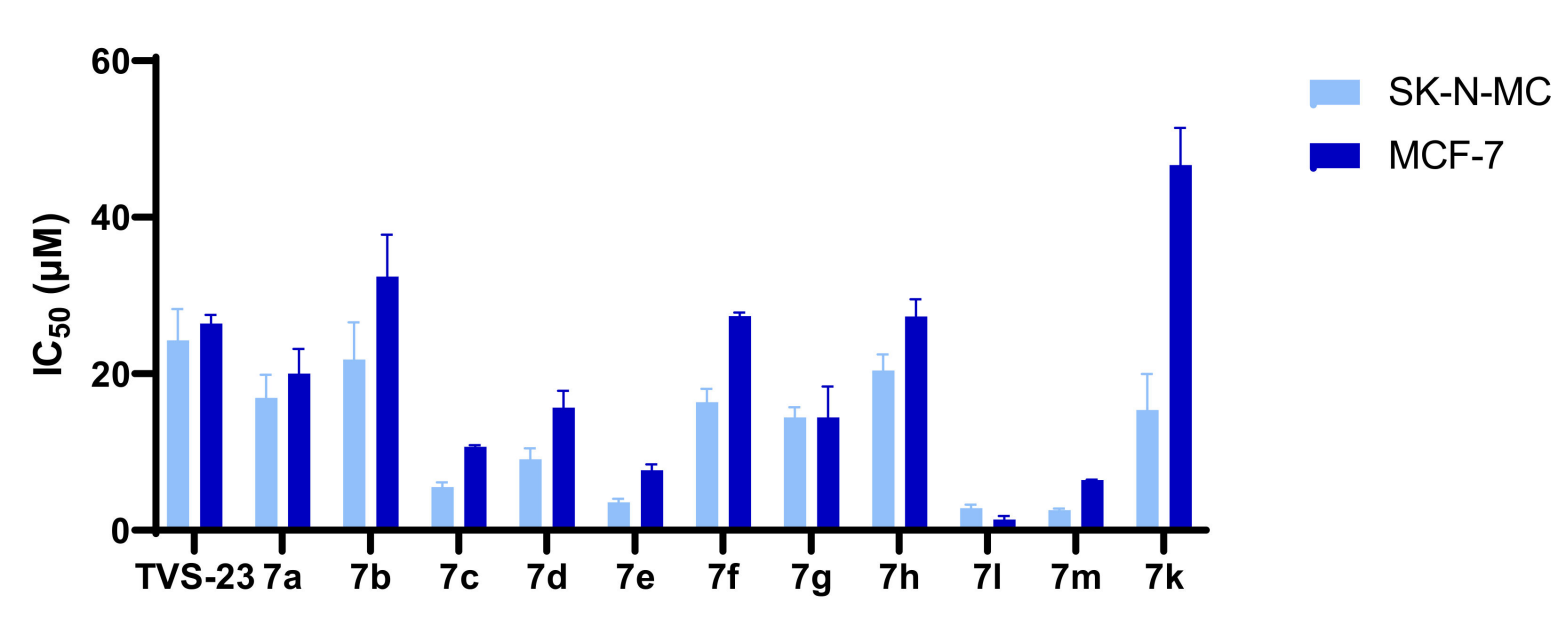
Antiproliferative activity in MCF-7 and SK-N-MC cancer cell lines in MTS assay after treatment with final compounds. Data are means ± SD of two independent experiments.

Overall, the results of biological evaluation of antiproliferative activities against the MCF-7 and SK-N-MC cell lines confirmed the importance of key pharmacophore features from most frequently appearing pharmacophore models in MD simulations, such as the presence of biphenyl moiety and cationic centre. Furthermore, we significantly improved the antiproliferative activity of virtual hit **TVS-23** by introducing an additional hydrophobic substituent on the *para* position of phenyl ring B and removal of *N*-methyl group on the piperidine ring (Figure 7). The results of the biological evaluation in two unrelated cancer cell lines demonstrate that Hsp90 is a widely usable therapeutic target for a number of different cancers, offering unique potential for the development of Hsp90 CTD inhibitors with a pan-cancer activity.

**Figure 8:**
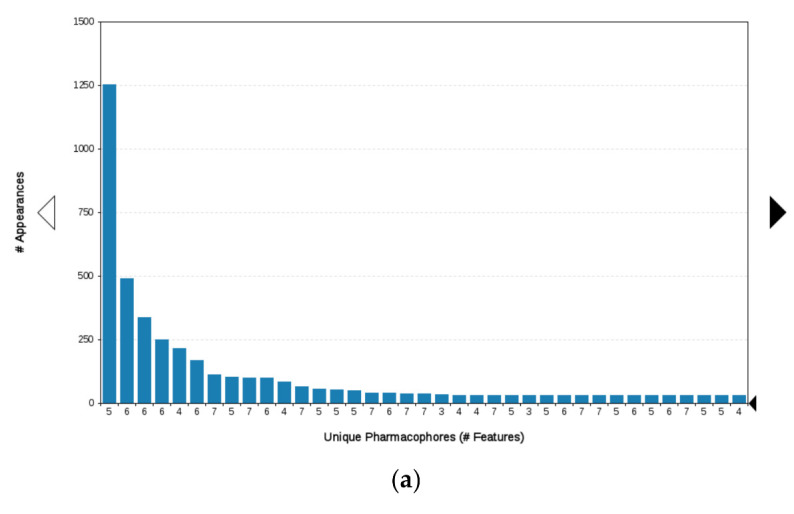

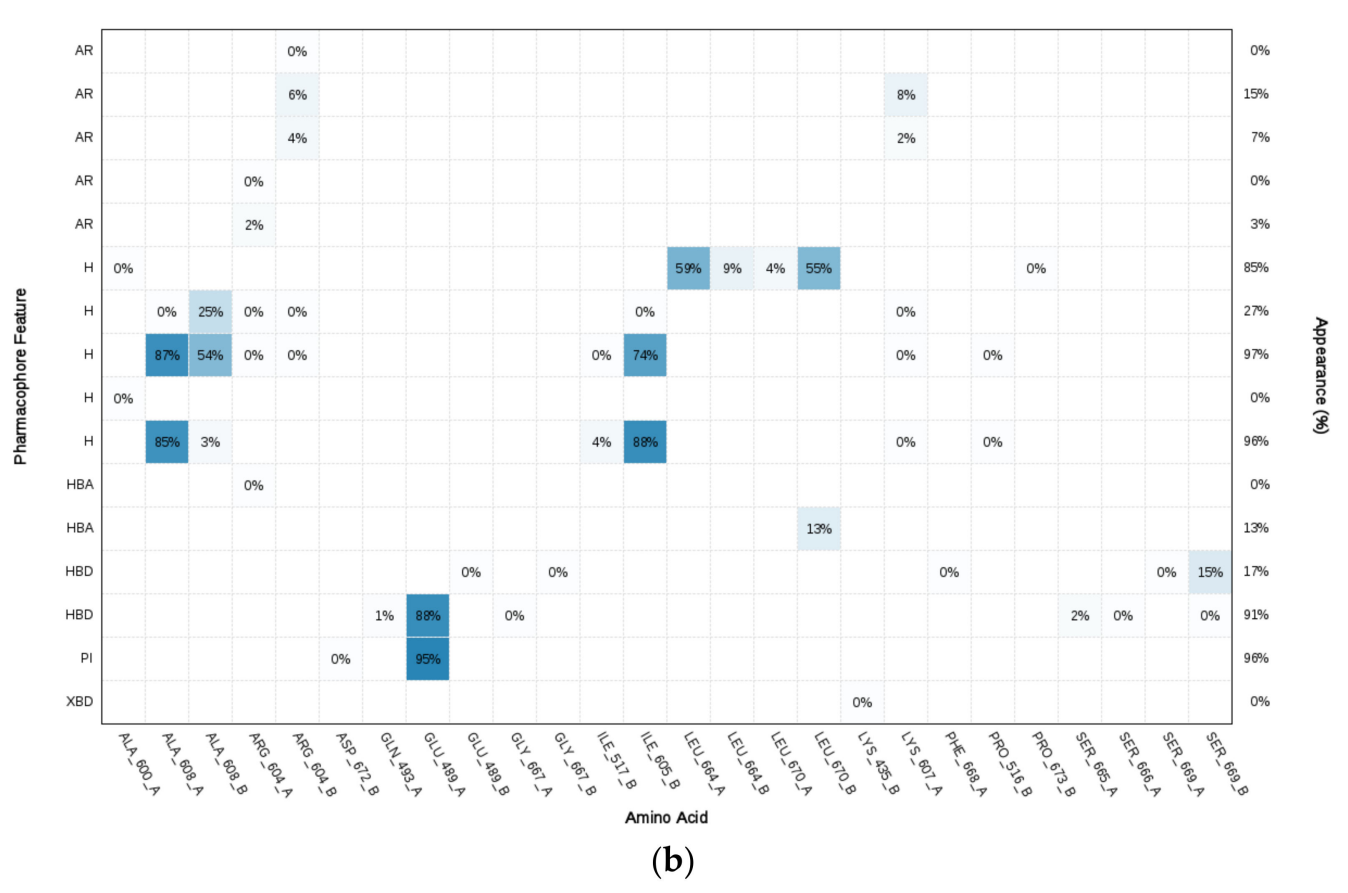
(**a**) Plot of the most frequent unique structure-based pharmacophore models derived from the molecular dynamics simulations of the Hsp90 C-terminal domain in complex with **7l**. The numbers below the bars indicate the numbers of interaction features observed during molecular dynamics simulation for the pharmacophore models. (**b**) MD Interaction map obtained by analysing interactions of **7l** with Hsp90 CTD binding site residues in the 1 µs MD simulation trajectory. Amino acid name and numbering is shown on *x*-axis, pharmacophore feature type on the left *y*-axis (H—hydrophobic, HBA—hydrogen bond acceptor, HBD—hydrogen bond donor, PI—positive ionizable, AR—aromatic), % appearance on the right *y*-axis.

**Figure 9:**
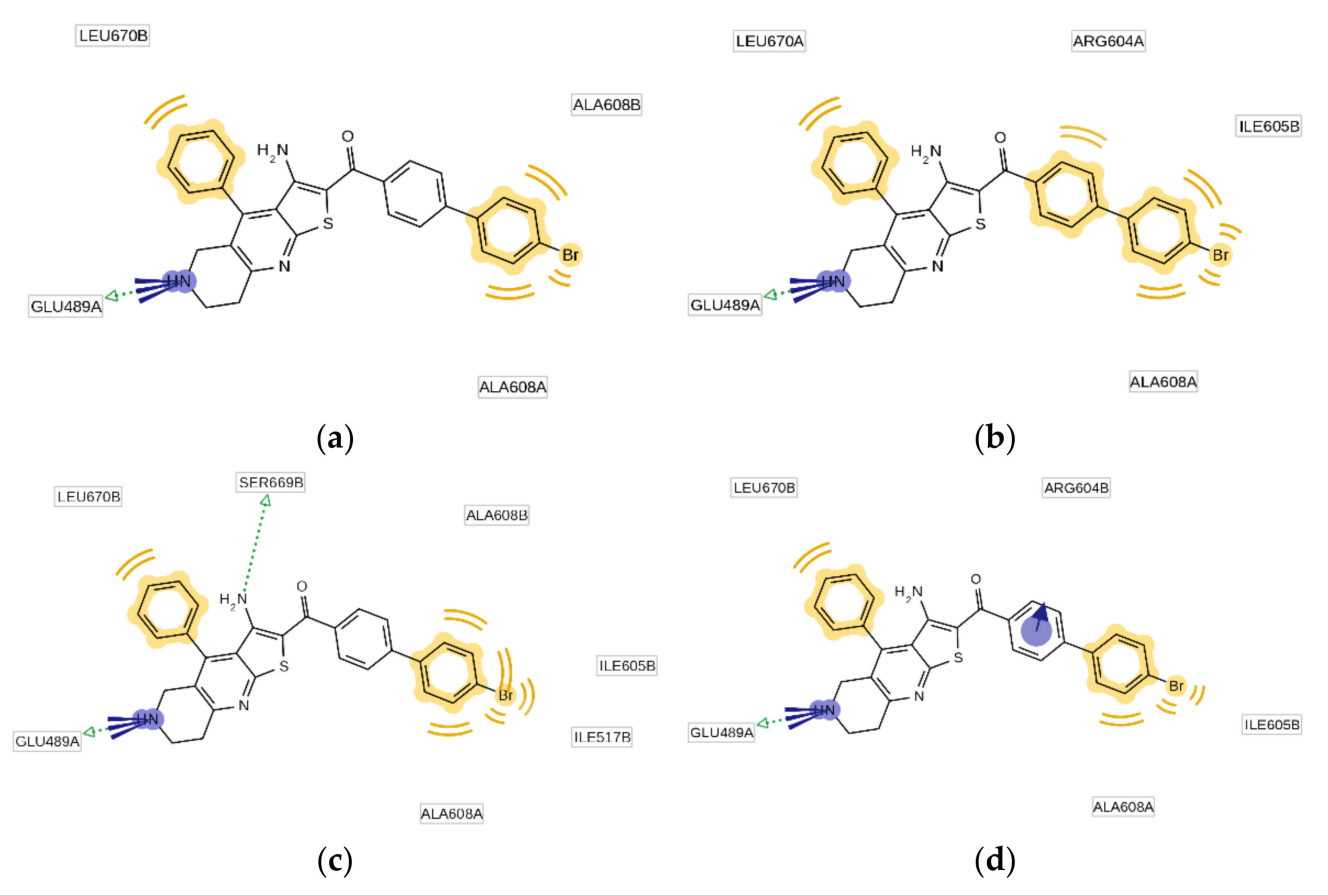
Schematic representation of the four most frequently appearing pharmacophore models during the MD trajectory (**a**–**d**).

The binding mode of the most potent **TVS-23** analogue, **7l,** was studied by a combination of docking, MD simulation and pharmacophore modelling as presented for the hit compound **TVS-23**. Analysis of the MD trajectory using the MD analysis tools in LigandScout Expert 4.4. revealed additional interactions in the Hsp90 CTD binding site (Figure 8 and Figure 9). The most common pharmacophore model occurred more than 1250 times and contains five pharmacophore features, namely, three hydrophobic features interacting with Ala608A, Ala608B and Leu670B, a positive ionisable group and a hydrogen bond donor interacting with Glu489A (Figure 9). These interactions are present during most of the simulation time. Additional interactions present in the next three most frequent models are the cation-π interaction with Arg604B and the hydrogen bond with Ser669B (Figure 9). Thus, the increased number of interactions between Hsp90 and **7l** is consistent with experimental data showing that **7l** is more potent than the hit compound **TVS-23**.

## 4. Conclusions

In the absence of a crystal structure of an Hsp90-CTD inhibitor complex, structure-based virtual screening was used to discover new classes of Hsp90 CTD inhibitors. The binding mode of the virtual hit **TVS-23** was investigated using molecular dynamics simulations. This revealed that the most important interactions with amino acid residues are an ionic interaction between piperidine and Glu489A and a hydrophobic interaction of biphenyl with Ala608A and Ala608B. Other interactions include a hydrogen bond between the carbonyl group and Ser669A and a hydrophobic interaction between thiophene and Leu670A. A series of **TVS-23** analogues were synthesised to further investigate SAR. It was found that the hydrophobic interaction between the biphenyl group and Ala608A is critical for the antiproliferative activity. Furthermore, we discovered that an additional hydrophobic substituent at the *para* position of the phenyl ring increased the antiproliferative activity to the low micromolar range. The discovery of **TVS-23** and its more potent analogues expands the diversity of Hsp90 CTD scaffolds and proves that structure-based virtual screening is a successful tool for discovering novel Hsp90 CTD inhibitors with antiproliferative activity in various cancer cell lines.

## Figures and Tables

**Table 1:** IC_50_ values for the antiproliferative activity of compounds **6e** and **6d** in the MCF-7 and SK-N-MC cell lines: exploration of importance of the phenyl ring B.

Compound	Structure	IC_50_ (µM)	
		MCF-7	SK-N-MC
**TVS-23**		26.4 ± 1.1	24.3 ± 4.0
17-DMAG *		0.9 ± 1	0.01 ± 0.007
**6e**	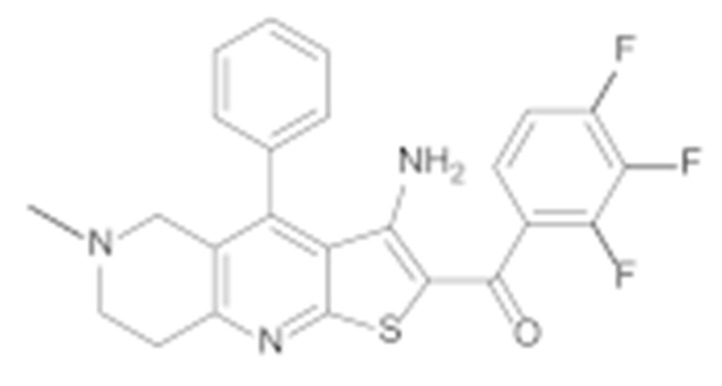	>50	>50
**6d**	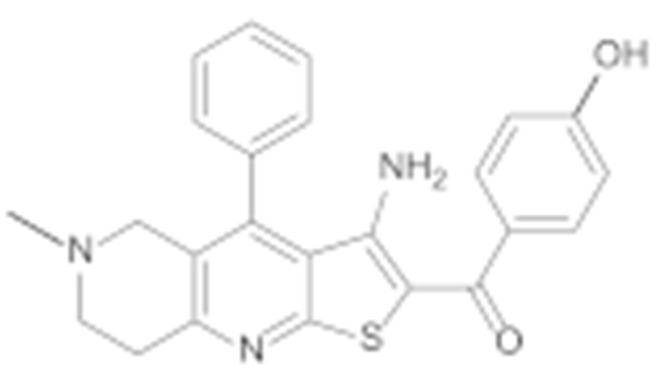	>50	>50

* 17-DMAG, 17-Dimethylaminoethylamino-17-demethoxygeldanamycin (Hsp90 NTD inhibitor, used as a positive control).

**Table 2:** IC_50_ values for the antiproliferative activity of compounds **7a**–**h** in the MCF-7 and SK-N-MC cell lines: exploration of SAR with modifications of the phenyl ring B.

Compound	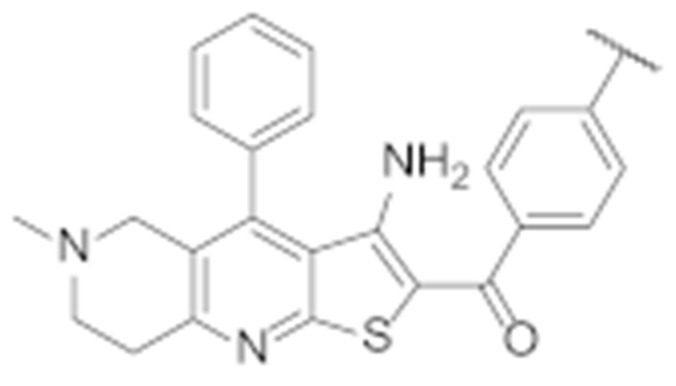	IC_50_ (µM)	
		MCF-7	SK-N-MC
**7a**	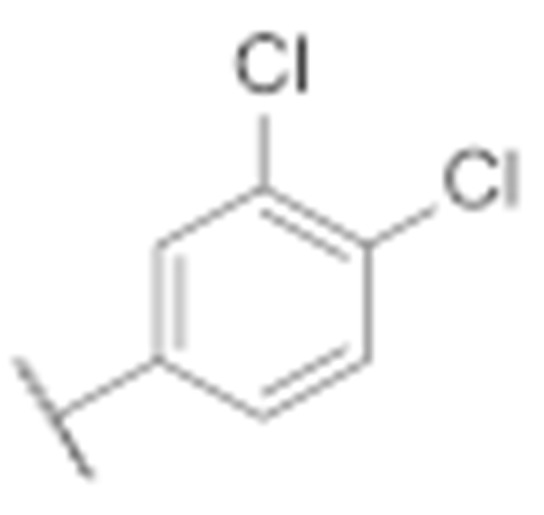	20.0 ± 3.1	16.9 ± 2.9
**7b**	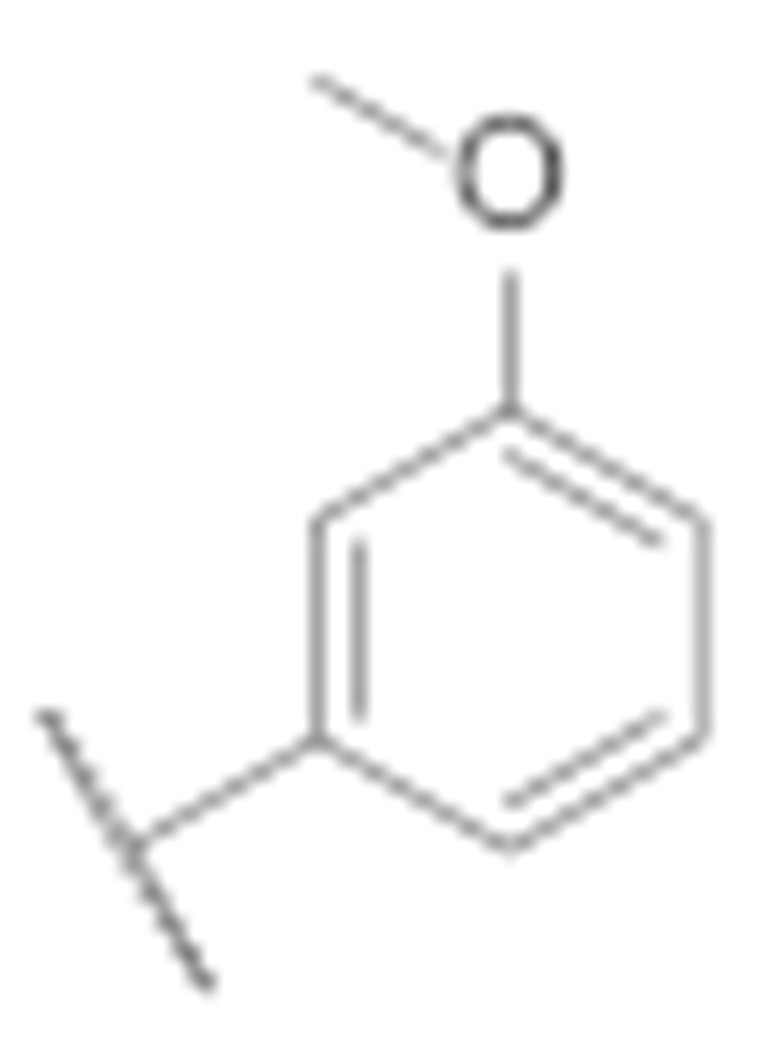	32.4 ± 5.4	21.8 ± 4.8
**7c**	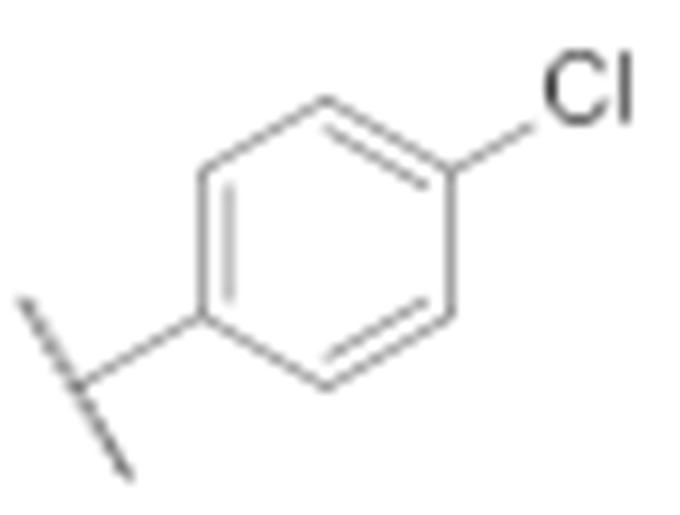	10.6 ± 0.3	5.5 ± 0.6
**7d**	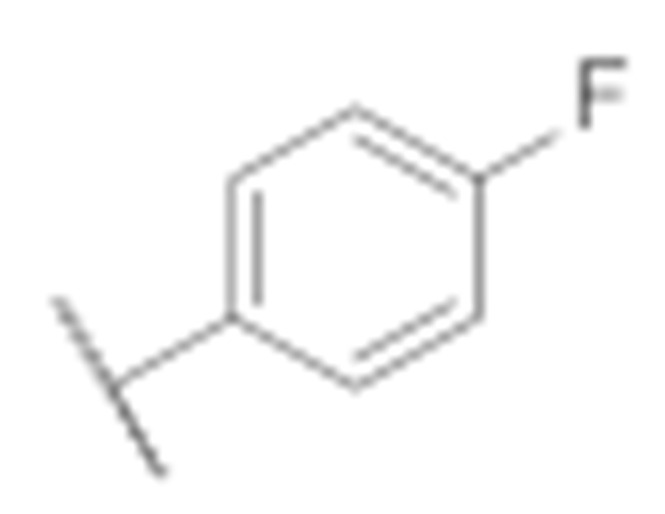	15.7 ± 2.2	9.1 ± 1.4
**7e**	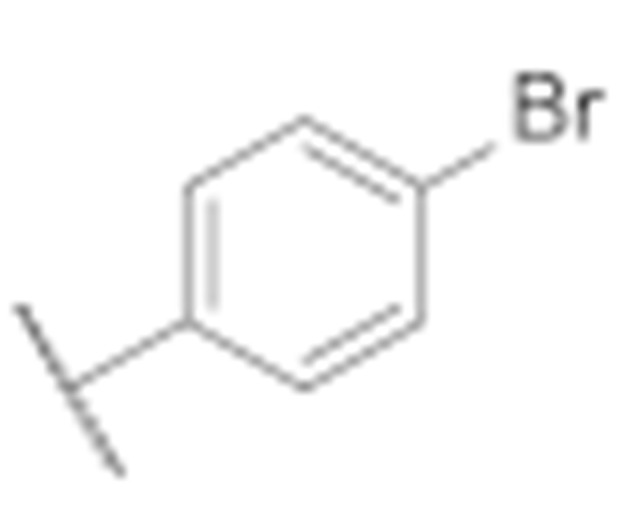	7.7 ± 0.8	3.6 ± 0.5
**7f**	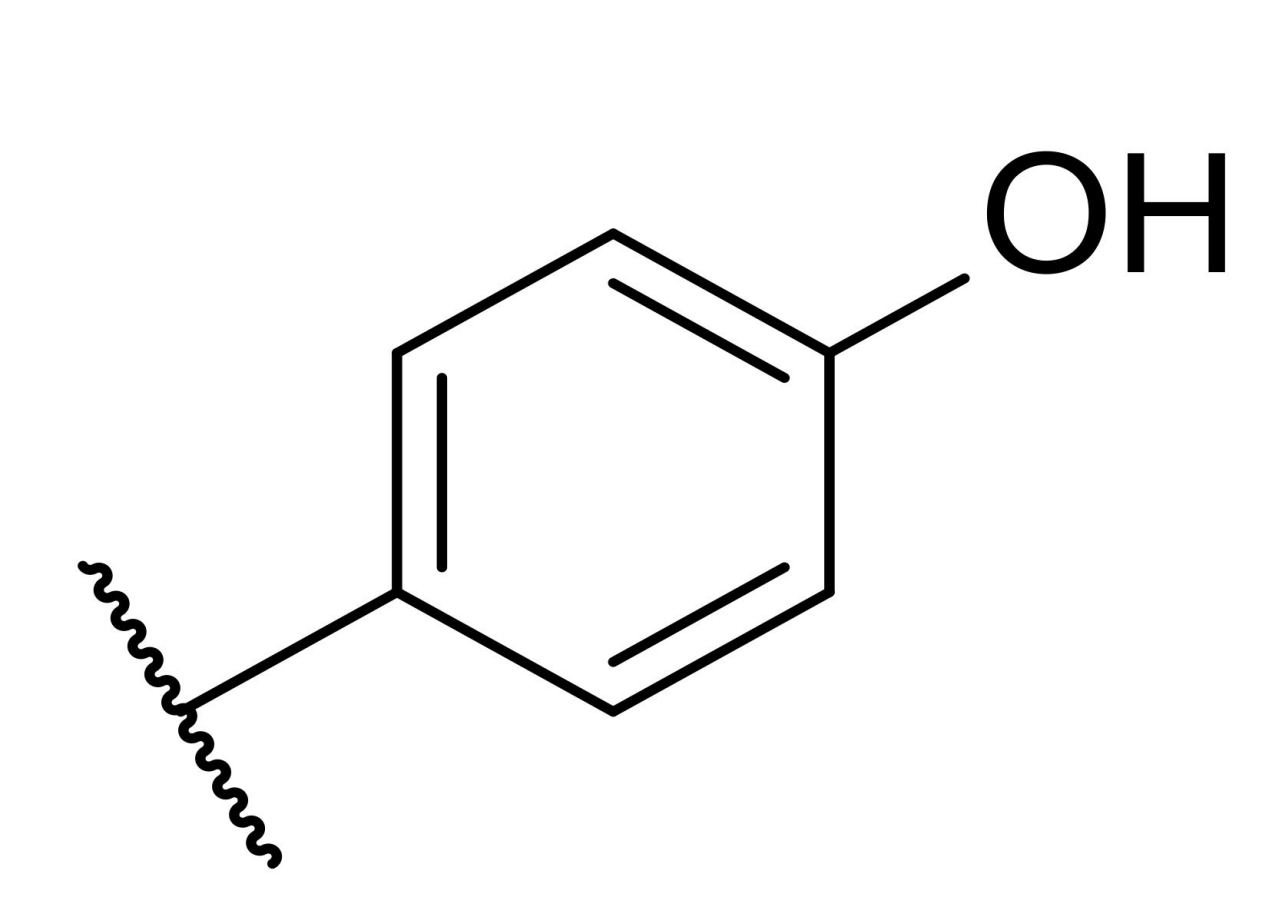	27.4 ± 0.5	16.4 ± 1.7
**7g**	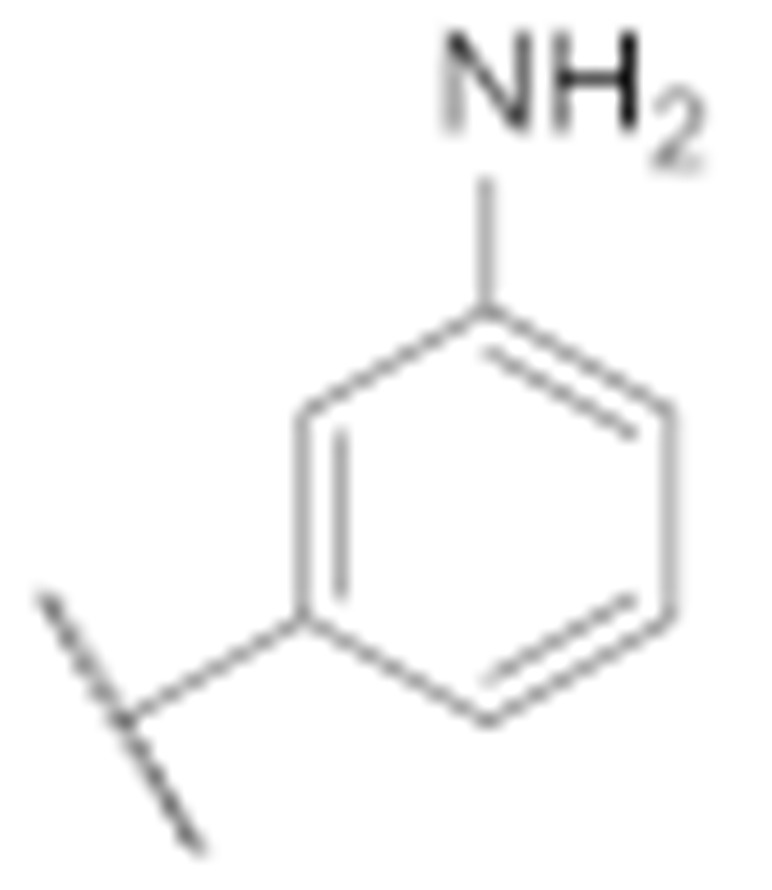	14.4 ± 3.9	14.4 ± 1.3
**7h**	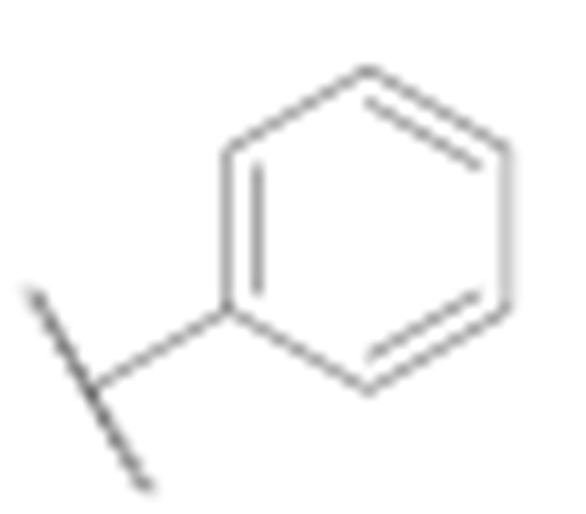	27.3 ± 2.2	20.5 ± 2.0

**Table 3:** IC_50_ values for the antiproliferative activity of compounds **7l–k** in the MCF-7 and SK-N-MC cell lines.

Compound	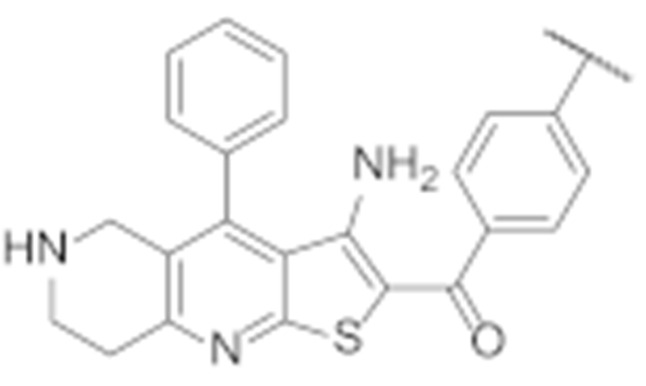	IC_50_ (µM)	
		MCF-7	SK-N-MC
**7l**	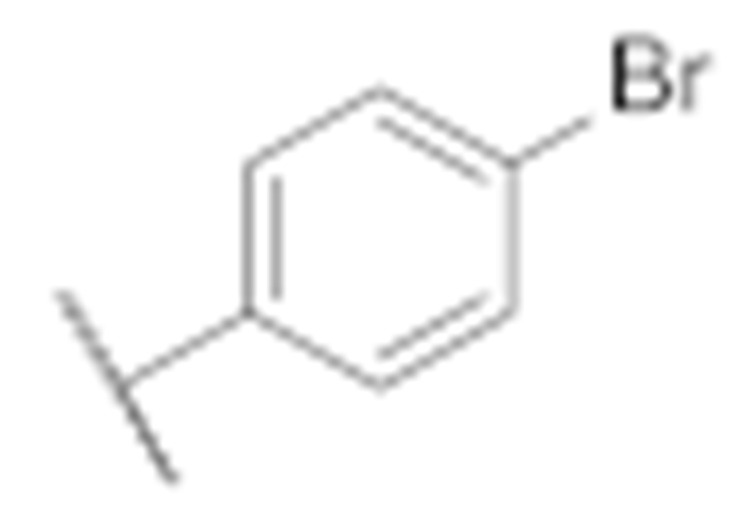	1.4 ± 0.4	2.8 ± 0.4
**7m**	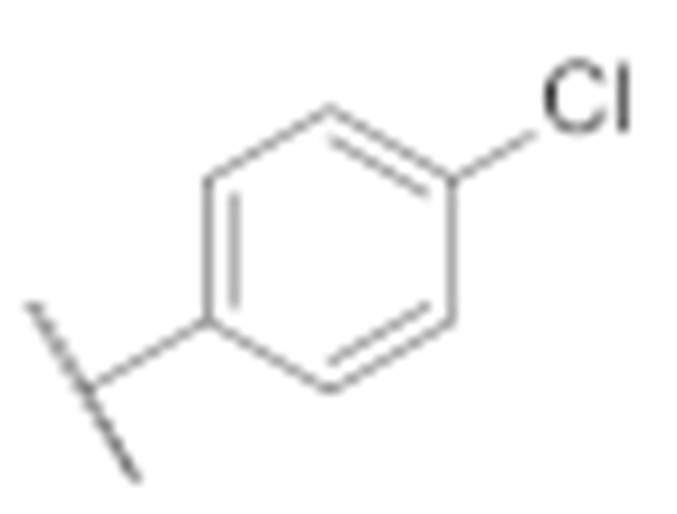	6.4 ± 0.1	2.6 ± 0.2
**7k**	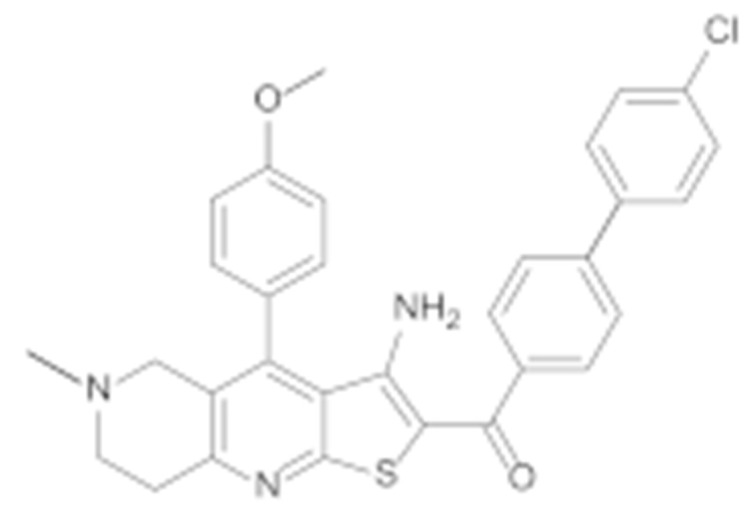	46.7 ± 4.8	15.4 ± 4.6

## Data Availability

The data presented in this study are available on request from the corresponding author.

## Supplementary Materials

The following supporting information can be downloaded at: https://www.mdpi.com/article/10.3390/biom12070884/s1, Figure S1: ^1^H NMR spectrum of compound **3a,** Figure S2: ^1^H NMR spectrum of compound **4a,** Figure S3: ^1^H NMR spectrum of compound **5a,** Figure S4: ^1^H NMR spectrum of compound **6a**, Figure S5: ^1^H NMR spectrum of compound **7a**, Figure S6: ^13^C NMR spectrum of compound **7a**, Figure S7: ^1^H NMR spectrum of compound **7b**, Figure S8: ^13^C NMR spectrum of compound **7b**, Figure S9: HPLC spectrum of **7b**, Figure S10: ^1^H NMR spectrum of compound **7c**, Figure S11: ^13^C NMR spectrum of compound **7c**, Figure S12: HPLC spectrum of compound **7c**, Figure S13: ^1^H NMR spectrum of compound **7d**, Figure S14:^13^C NMR spectrum of compound **7d**, Figure S15: HPLC spectrum of compound **7d**, Figure S16: ^1^H NMR spectrum of compound **7f**, Figure S17: ^13^C NMR spectrum of compound **7f**, Figure S18: ^1^H NMR spectrum of compound **7g**, Figure S19: ^1^H NMR spectrum of compound **7h**, Figure S20: ^13^C NMR spectrum of compound **7h**, Figure S21: HPLC spectrum of compound **7h**, Figure S22: ^1^H NMR spectrum of compound **7e**, Figure S23: ^13^C NMR spectrum of compound **7e**, Figure S24: HPLC spectrum of compound **7e**, Figure S25: ^1^H NMR spectrum of compound **7l**, Figure S26: ^1^H NMR spectrum of compound **7m**, Figure S27: ^1^H NMR spectrum of compound **7k**, Figure S28: IC_50_ curve of compound **7e** in MCF-7 cell line, Figure S29: IC_50_ curve of compound **7c** in SK-N-MC cell line, Figure S30: Western blot of validation of overexpression of Hsp90 in MCF-7 and SK-N-MC cell lines; Figure S31: Percentage of active luciferase in luciferase refolding assay after treatment with compound **7l**.

## Appendix A

**General procedure A**

The 2-Cyanothioacetamide (1 eq) and the corresponding benzaldehyde (1 eq) and a catalytic amount of Et_3_N were dissolved in absolute EtOH (1.5 mL/mmol). The reaction mixture was stirred at 50 °C for 1 h. The reaction mixture was cooled to room temperature, and the yellow precipitate was filtered off. The precipitate was triturated with hot EtOH and used in the next step without further purification.

**General procedure B**

Benzylidenecyanothioacetamide (1 eq), the corresponding piperidin-4-one (1 eq) and a catalytic amount of piperidine were dissolved in EtOH (3 mL/mmol) and refluxed for 1 h. The reaction mixture was allowed to cool to room temperature and the orange precipitate was filtered off.

**General procedure C**

Intermediates **4a–c** (1 eq), substituted 2-bromoacetophenone (1 eq) and anhydrous sodium acetate (2.5 mmol) were dissolved in EtOH and refluxed for 1 h. The reaction mixture was cooled to room temperature, and the orange precipitate was filtered off.

**General procedure D**

Intermediates **5a–e** (1 eq) were dissolved in absolute EtOH, a catalytic amount of 4 M NaOMe was added and the reaction mixture was refluxed for 30 min. The reaction mixture was allowed to cool to room temperature, and the orange precipitate was filtered off.

**General procedure E**

To a solution of intermediates **6a–c** (1 eq) in a mixture of 1,4-dioxane and H_2_O (2:1) respective boronic acid (1.2 eq) and K_2_CO_3_ (2.5 eq) were added. The reaction mixture was degassed in an ultrasonic bath, then tetrakis(triphenylphosphine)palladium(0) (5 mol%) was added. The reaction mixture was stirred under an argon atmosphere at 100 °C overnight. The solvent was evaporated in vacuo. The residue was dissolved in dichloromethane and washed with water. The organic phase was dried over Na_2_SO_4_, filtered and the solvent was evaporated in vacuo. The residue was purified by reverse-phase flash chromatography.

**General procedure F**

Trifluoroacetic acid (10 eq) was added to a solution of Boc-protected intermediates (1 eq) in dichloromethane. The reaction mixture was stirred at r.t. for 2 days. The organic phase was washed with 1 M NaOH, dried over Na_2_SO_4_, filtered and the solvent evaporated in vacuo.

**2-Cyano-3-phenylprop-2-enethioamide (3a)**

Synthesised according to general procedure A, using benzaldehyde (3.78 g, 35.71 mmol) as reagent. Yield: 4.87 g (72.7%); yellow amorphous solid; ^1^H NMR (400 MHz, DMSO-*d*_6_) δ 7.66–7.49 (m, 1H, Ar-H), 7.44–7.25 (m, 4H, 4 × Ar-H), 6.84 (s, 1H, CH); MS (ESI-) *m*/*z* = 186.9 ([M-H]^−^).

**2-Cyano-3-(4-methoxyphenyl)prop-2-enethioamide (3b)**

Synthesised according to general procedure A using 4-methoxybenzaldehyde (1.53 g, 11.25 mmol) as reagent. Yield: 1.54 g (63.1%); yellow amorphous solid; ^1^H NMR (400 MHz, DMSO-*d*_6_) δ 8.07 (s, 1H, Ar-H), 7.99–7.95 (m, 2H, 2 × Ar-H), 7.14 (d, *J* = 8.9 Hz, 2H, 2 × Ar-H), 3.86 (s, 3H, O-CH_3_); MS (ESI-) *m*/*z* = 217.0 ([M-H]^−^).

**6-Methyl-4-phenyl-2-thioxo-2,4a,5,6,7,8-hexahydro-1,6-naphthyridine-3-carbonitrile (4a)**

Synthesised according to general procedure B using compound 3a (1.87 g, 10 mmol) and *N*-methylpiperidin-4-one (1.13 g, 10 mmol) as reagents. Yield: 750 mg (26.7%); orange amorphous solid; ^1^H NMR (400 MHz, DMSO-*d*_6_) δ 7.92–7.46 (m, 3H, 3 × Ar-H), 7.45–7.15 (m, 2H, 2 × Ar-H), 2.88 (d, *J* = 8.4 Hz, 4H, 2 × piperidine-CH_2_), 2.61 (t, *J* = 5.7 Hz, 2H, piperidine-CH_2_), 2.20 (s, 3H, N-CH_3_); MS (ESI+) *m*/*z* = 282.0 ([M+H]^+^).

***tert*-Butyl 3-cyano-4-phenyl-2-thioxo-4a,5,7,8-tetrahydro-1,6-naphthyridine-6(2*H*)-carboxylate (4b)**

Synthesised according to general procedure B using compound **3a** (1.5 g, 7.98 mmol) and *tert*-butyl 4-oxopiperidine-1-carboxylate (1.59 g, 7.98 mmol) as reagents. Yield: 430 mg (14.4%); orange amorphous solid; ^1^H NMR (400 MHz, DMSO-*d*_6_) δ 14.21 (s, 1H, Ar-H), 7.60–7.52 (m, 3H, 3 × Ar-H), 7.44–7.34 (m, 2H, 2 × Ar-H), 3.90 (s, 2H, piperidine-CH_2_), 3.56 (t, *J* = 6.0 Hz, 2H, piperidine-CH_2_), 2.85 (t, *J* = 5.9 Hz, 2H, piperidine-CH_2_), 1.34 (s, 9H, 3 × CH_3_); MS (ESI+) *m*/*z* = 368.1 ([M+H]^+^).

**4-(4-Methoxyphenyl)-6-methyl-2-thioxo-2,4a,5,6,7,8-hexahydro-1,6-naphthyridine-3-carbonitrile (4c)**

Synthesised according to general procedure B using compound **3b** and *N*-methylpiperidin-4-one as reagents. Yield: 380 mg (10.8%); orange amorphous solid; ^1^H NMR (400 MHz, DMSO-*d*_6_) δ 7.32 (d, *J* = 8.7 Hz, 2H, 2 × Ar-H), 7.08 (d, *J* = 8.8 Hz, 2H, 2 × Ar-H), 3.83 (s, 3H, O-CH_3_), 2.94 (s, 2H, piperidine-CH_2_), 2.86 (t, *J* = 5.9 Hz, 2H, piperidine-CH_2_), 2.64–2.58 (m, 2H, piperidine-CH_2_), 2.22 (s, 3H, N-CH_3_); MS (ESI+) *m*/*z* = 312.1 ([M+H]^+^).

**2-((2-(4-Bromophenyl)-2-oxoethyl)thio)-6-methyl-4-phenyl-2,4a,5,6,7,8-hexahydro-1,6-naphthyridine-3-carbonitrile (5a)**

Synthesised according to general procedure C using compound **4a** (480 mg, 1.71 mmol) and 2,4′-dibromoacetophenone (474 mg, 1.71 mmol) as reagents. Yield: 718 mg (88.0%); yellow amorphous solid; ^1^H NMR (400 MHz, DMSO-*d*_6_) δ 8.06–8.00 (m, 2H, 2 × Ar-H), 7.81 (d, *J* = 8.6 Hz, 2H, 2 × Ar-H), 7.59–7.50 (m, 3H, 3 × Ar-H), 7.45–7.34 (m, 2H, 2 × Ar-H), 4.83 (s, 2H, S-CH_2_), 3.09 (s, 2H, piperidine-CH_2_), 2.61 (d, *J* = 5.1 Hz, 2H, piperidine-CH_2_), 2.58 (d, *J* = 4.9 Hz, 2H, piperidine-CH_2_), 2.19 (s, 3H, N-CH_3_); MS (ESI+) *m*/*z* = 478.0 [M+H]^+^).

***tert*-Butyl 2-((2-(4-bromophenyl)-2-oxoethyl)thio)-3-cyano-4-phenyl-4a,5,7,8-tetrahydro-1,6-naphthyridine-6(2*H*)-carboxylate (5b)**

Synthesised according to general procedure C using compound **4b** (432 mg, 1.21 mmol) and 2,4′-dibromoacetophenone (336 mg, 1.21 mmol) as reagents. Yield: 413 mg (59.2%); yellow amorphous solid; ^1^H NMR (400 MHz, DMSO-*d*_6_) δ 8.06–7.99 (m, 2H, 2 × Ar-H), 7.84–7.79 (m, 2H, 2 × Ar-H), 7.58 (dd, *J* = 5.8, 1.6 Hz, 3H, 3 × Ar-H), 7.41 (dd, *J* = 7.3, 2.3 Hz, 2H, 2 × Ar-H), 4.85 (s, 2H, S-CH_2_), 4.13 (s, 2H, piperidine-CH_2_), 3.54 (t, *J* = 6.1 Hz, 2H, piperidine-CH_2_), 2.61 (t, *J* = 6.1 Hz, 2H, piperidine-CH_2_), 1.32 (s, 9H, 3 × CH_3_); MS (ESI+) *m*/*z* = 586.0 ([M+Na]^+^).

**2-((2-(4-Bromophenyl)-2-oxoethyl)thio)-4-(4-methoxyphenyl)-6-methyl-2,4a,5,6,7,8-hexahydro-1,6-naphthyridine-3-carbonitrile (5c)**

Synthesised according to general procedure C using compound **4c** and 2,4′-dibromoacetophenone as reagents. Yield: 435 mg (65.0%); yellow amorphous solid; ^1^H NMR (400 MHz, *Chloroform-d*) δ 8.00–7.90 (m, 2H, 2 × Ar-H), 7.70–7.61 (m, 2H, 2 × Ar-H), 7.21–7.11 (m, 2H, 2 × Ar-H), 7.04–6.94 (m, 2H, 2 × Ar-H), 4.58 (s, 2H, S-CH_2_), 3.86 (s, 3H, O-CH_3_), 3.22 (s, 2H, piperidine-CH_2_), 2.77 (t, *J* = 6.0 Hz, 2H, piperidine-CH_2_), 2.65 (t, *J* = 5.9 Hz, 2H, piperidine-CH_2_), 2.33 (s, 3H, N-CH_3_); MS (ESI+) *m*/*z* = 510.0 ([M+H]^+^).

**2-((2-(3-Hydroxyphenyl)-2-oxoethyl)thio)-6-methyl-4-phenyl-2,4a,5,6,7,8-hexahydro-1,6-naphthyridine-3-carbonitrile (5d)**

Synthesised according to general procedure C using compound **4a** (100 mg, 0.355 mmol) and 2-bromo-3′-hydroxyacetophenone (76 mg, 0.355 mmol) as reagents. Yield: 60 mg (47.5%); yellow amorphous solid; ^1^H NMR (400 MHz, DMSO-*d*_6_) δ 7.59–7.51 (m, 4H, 4 × Ar-H), 7.43–7.37 (m, 4H, 4 × Ar-H), 7.08 (ddd, *J* = 8.1, 2.5, 1.0 Hz, 1H, Ar-H), 4.81 (s, 2H, S-CH_2_), 3.10 (s, 2H, piperidine-CH_2_), 2.73–2.66 (m, 2H, piperidine-CH_2_), 2.59 (t, *J* = 5.9 Hz, 2H, piperidine-CH_2_), 2.20 (s, 3H, N-CH_3_), MS (ESI+) *m*/*z* = 418.0 ([M+H]^+^).

**6-Methyl-2-((2-oxo-2-(2,3,4-trifluorophenyl)ethyl)thio)-4-phenyl-2,4a,5,6,7,8-hexahydro-1,6-naphthyridine-3-carbonitrile (5e)**

Synthesised according to general procedure C using compound **4a** (70 mg, 0.249 mmol) and 2-bromo-2′,3′,4′-trifluoroacetophenone (63 mg, 0.249 mmol) as reagents. Yield: 12 mg (8.3%); yellow amorphous solid; ^1^H NMR (400 MHz, DMSO-*d*_6_) δ 8.03 (ddd, *J* = 10.8, 9.0, 6.5 Hz, 1H, Ar-H), 7.83 (td, *J* = 10.6, 6.4 Hz, 1H, Ar-H), 7.60–7.50 (m, 3H, 3 × Ar-H), 7.39 (dd, *J* = 7.2, 2.3 Hz, 2H, 2 × Ar-H), 4.73 (s, 2H, S-CH_2_), 3.11 (s, 2H, piperidine-CH_2_), 2.73–2.66 (m, 2H, piperidine-CH_2_), 2.61 (t, *J* = 5.8 Hz, 2H, piperidine-CH_2_), 2.21 (s, 3H, N-CH_3_); MS (ESI+) *m*/*z* = 456.0 ([M+H]^+^).

**(3-Amino-6-methyl-4-phenyl-5,6,7,8-tetrahydrothieno [2,3-*b*][1,6]naphthyridin-2-yl)(4-bromophenyl)methanone (6a)**

Synthesised according to general procedure D using compound **5a** (718 mg, 1.5 mmol) as a reagent. Yield: 358 mg (86.9%); yellow amorphous solid; 1H NMR (400 MHz, DMSO-*d*_6_) δ 7.79–7.73 (m, 2H, 2 × Ar-H), 7.71–7.61 (m, 5H, 5 × Ar-H), 7.49–7.42 (m, 2H, 2 × Ar-H), 3.14 (s, 2H, piperidine-CH_2_), 3.10 (t, *J* = 5.9 Hz, 2H, piperidine-CH_2_), 2.71 (t, *J* = 5.9 Hz, 2H, piperidine-CH_2_), 2.24 (s, 3H, N-CH_3_); ^13^C NMR (101 MHz, Chloroform-*d*) δ 189.03, 160.70, 158.13, 151.22, 144.91, 139.73, 134.21, 131.52, 129.55, 129.45, 127.83, 125.67, 124.90, 119.93, 104.33, 55.46, 52.39, 46.04, 33.47; MS (ESI+) *m*/*z* = 478.0 ([M+H]^+^).

***tert*-Butyl 3-amino-2-(4-bromobenzoyl)-4-phenyl-7,8-dihydrothieno [2,3-*b*][1,6]naphthyridine-6(5*H*)-carboxylate (6b)**

Synthesised according to general procedure D using compound **5b** (413 mg, 0.715 mmol) as a reagent. Yield: 358 mg (86.9%); yellow amorphous solid; ^1^H NMR (400 MHz, Chloroform-*d*) δ 7.73–7.68 (m, 2H, 2 × Ar-H), 7.64–7.54 (m, 5H, 5 × Ar-H), 7.40–7.33 (m, 2H, 2 × Ar-H), 4.30 (s, 2H, piperidine-CH_2_), 3.77 (t, *J* = 6.1 Hz, 2H, piperidine-CH_2_), 3.17 (t, *J* = 6.1 Hz, 2H, piperidine-CH_2_), 1.44 (s, 9H, 3 × CH_3_); MS (ESI+) *m*/*z* = 564.0 ([M+H]^+^).

**(3-Amino-4-(4-methoxyphenyl)-6-methyl-5,6,7,8-tetrahydrothieno [2,3-*b*][1,6]naphthyridin-2-yl)(4-bromophenyl)methanone (6c)**

Synthesised according to general procedure D using compound **5c** (435 mg, 0.85 mmol) as a reagent. Yield: 367 mg (85.2%); yellow amorphous solid; ^1^H NMR (400 MHz, Chloroform-*d*) δ 7.73–7.67 (m, 2H, 2 × Ar-H), 7.62–7.57 (m, 2H, 2 × Ar-H), 7.26–7.21 (m, 2H, 2 × Ar-H), 7.13–7.08 (m, 2H, 2 × Ar-H), 3.93 (s, 3H, O-CH_3_), 3.27 (s, 2H, piperidine-CH_2_), 3.22 (d, *J* = 6.0 Hz, 2H, piperidine-CH_2_), 2.79 (t, *J* = 6.0 Hz, 2H, piperidine-CH_2_), 2.38 (s, 3H, N-CH_3_); MS (ESI+) *m*/*z* = 508.0 ([M+H]^+^).

**(3-Amino-6-methyl-4-phenyl-5,6,7,8-tetrahydrothieno [2,3-*b*][1,6]naphthyridin-2-yl)(3-hydroxyphenyl)methanone (6d)**

Synthesised according to general procedure D using compound **5d** (60 mg, 0.144 mmol) as a reagent. Yield: 11 mg (18.6%); yellow amorphous solid; ^1^H NMR (400 MHz, DMSO-*d*_6_) δ 7.68–7.59 (m, 3H, 3 × Ar-H), 7.47–7.42 (m, 2H, 2 × Ar-H), 7.28 (t, *J* = 7.8 Hz, 1H, Ar-H), 7.11–7.04 (m, 2H, 2 × Ar-H), 6.94–6.88 (m, 1H, Ar-H), 3.14 (s, 2H, piperidine-CH_2_), 3.09 (t, *J* = 6.0 Hz, 2H, piperidine-CH_2_), 2.71 (t, *J* = 5.9 Hz, 2H, piperidine-CH_2_), 2.24 (s, 3H, N-CH_3_); MS (ESI+) *m*/*z* = 416.0 ([M+H]^+^).

**(3-Amino-6-methyl-4-phenyl-5,6,7,8-tetrahydrothieno [2,3-*b*][1,6]naphthyridin-2-yl)(2,3,4-trifluorophenyl)methanone (6e)**

Synthesised according to general procedure D using compound **5e** (60 mg, 0.144 mmol) as a reagent. Yield: 5 mg (41.6%); yellow amorphous solid; ^1^H NMR (400 MHz, DMSO-*d*_6_) δ 7.88–7.78 (m, 1H, Ar-H), 7.52–7.42 (m, 3H, 3 × Ar-H), 7.27 (dd, *J* = 6.6, 3.0 Hz, 2H, 2 × Ar-H), 6.61 (dd, *J* = 13.0, 7.7 Hz, 1H, Ar-H), 3.25 (s, 2H, piperidine-CH_2_), 3.09 (d, *J* = 6.5 Hz, 2H, piperidine-CH_2_), 2.73 (t, *J* = 6.0 Hz, 2H, piperidine-CH_2_), 2.27 (s, 3H, N-CH_3_); MS (ESI+) *m*/*z* = 454.0 ([M+H]^+^).

**(3-Amino-6-methyl-4-phenyl-5,6,7,8-tetrahydrothieno [2,3-*b*][1,6]naphthyridin-2-yl)(3′,4′-dichloro-[1,1′-biphenyl]-4-yl)methanone (7a)**

Synthesised according to general procedure E using compound **6a** (100 mg, 0.205 mmol) and (3,4-dichlorophenyl)boronic acid (63 mg, 0.246 mmol) as reagents. Yield: 42 mg (37.1%); orange amorphous solid; ^1^H NMR (400 MHz, DMSO-*d*_6_) δ 8.07 (d, *J* = 1.8 Hz, 1H, Ar-H), 7.90 (d, *J* = 8.5 Hz, 2H, 2 × Ar-H), 7.84 (d, *J* = 8.4 Hz, 2H, 2 × Ar-H), 7.79–7.76 (m, 1H, Ar-H), 7.65 (dt, *J* = 5.9, 2.0 Hz, 3H, 3 × Ar-H), 7.49–7.43 (m, 2H, 2 × Ar-H), 3.15 (s, 2H, piperidine-CH_2_), 3.10 (t, *J* = 5.9 Hz, 2H, piperidine-CH_2_), 2.72 (t, *J* = 6.0 Hz, 2H, piperidine-CH_2_), 2.25 (s, 3H, N-CH_3_); ^13^C NMR (101 MHz, Chloroform-*d*) δ 189.63, 160.79, 158.05, 151.11, 144.93, 141.23, 140.57, 140.27, 134.30, 133.05, 132.12, 131.55, 130.84, 129.58, 129.48, 129.10, 128.63, 127.88, 126.87, 126.49, 124.87, 120.03, 104.68, 55.50, 52.43, 46.07, 33.50; HRMS (ESI+) for C_30_H_23_Cl_2_N_3_OS (M+H^+^): calculated 554.10117; measured 554.09945; HPLC: *t*_R_ = 5.95 min (95.3% at 254 nm).

**(3-Amino-6-methyl-4-phenyl-5,6,7,8-tetrahydrothieno [2,3-*b*][1,6]naphthyridin-2-yl)(3′-methoxy-[1,1′-biphenyl]-4-yl)methanone (7b)**

Synthesised according to general procedure E using compound **6a** (50 mg, 0.1 mmol) and (3-methoxyphenyl)boronic acid (19 mg, 0.12 mmol) as reagents. Yield: 10 mg (19.8%); orange amorphous solid; ^1^H NMR (400 MHz, DMSO-*d*_6_) δ 7.84 (s, 4H, 4 × Ar-H), 7.65 (d, *J* = 6.5 Hz, 3H, 3 × Ar-H), 7.54–7.39 (m, 3H, 3 × Ar-H), 7.38–7.27 (m, 2H, 2 × Ar-H), 7.00 (dd, *J* = 8.4, 2.5 Hz, 1H, Ar-H), 3.85 (s, 3H, O-CH_3_), 3.16 (s, 2H, piperidine-CH_2_), 3.11 (t, *J* = 6.2 Hz, 2H, piperidine-CH_2_), 2.72 (t, *J* = 5.6 Hz, 2H, piperidine-CH_2_), 2.25 (s, 3H, N-CH_3_); ^13^C NMR (101 MHz, Chloroform-*d*) δ 189.99, 160.79, 160.02, 157.88, 150.89, 144.86, 143.71, 141.78, 139.92, 134.37, 129.91, 129.55, 129.44, 128.44, 127.90, 127.06, 124.78, 120.08, 119.79, 113.40, 112.90, 104.84, 67.11, 55.38, 52.45, 46.07, 33.49; HRMS (ESI+) for C_31_H_27_N_3_O_2_S (M+H^+^): calculated 506.18826; measured 506.18967; HPLC: *t*_R_ = 5.42 min (95.2% at 254 nm).

**(3-Amino-6-methyl-4-phenyl-5,6,7,8-tetrahydrothieno [2,3-*b*][1,6]naphthyridin-2-yl)(4′-chloro-[1,1′-biphenyl]-4-yl)methanone (7c)**

Synthesised according to general procedure E using compound **6a** (80 mg, 0.167 mmol) and (4-chlorophenyl)boronic acid (31 mg, 0.2 mmol) as reagents. Yield: 18 mg (21.1%); orange amorphous solid; ^1^H NMR (400 MHz, DMSO-*d*_6_) δ 7.85 (s, 4H, 4 × Ar-H), 7.80 (d, *J* = 8.3 Hz, 2H, 2 × Ar-H), 7.65 (d, *J* = 6.4 Hz, 3H, 2 × Ar-H), 7.57 (d, *J* = 8.2 Hz, 2H, 2 × Ar-H), 7.46 (d, *J* = 6.7 Hz, 2H, 2 × Ar-H), 6.60 (s, 1H, Ar-H), 3.15 (s, 2H, piperidine-CH_2_), 3.10 (t, *J* = 6.0 Hz, 2H, piperidine-CH_2_), 2.71 (t, *J* = 5.9 Hz, 2H, piperidine-CH_2_), 2.25 (s, 3H, N-CH_3_); ^13^C NMR (101 MHz, Chloroform-*d*) δ 189.82, 160.77, 157.95, 150.99, 144.91, 142.54, 140.07, 138.70, 134.32, 134.07, 129.56, 129.47, 129.07, 128.56, 128.53, 127.89, 126.85, 124.81, 120.06, 104.76, 55.49, 52.43, 46.06, 33.47; HRMS (ESI+) for C_30_H_24_ClN_3_OS (M+H^+^): calculated 510.13849; measured 510.14014; HPLC: *t*_R_ = 5.73 min (96.1% at 254 nm).

**(3-Amino-6-methyl-4-phenyl-5,6,7,8-tetrahydrothieno [2,3-*b*][1,6]naphthyridin-2-yl)(4′-fluoro-[1,1′-biphenyl]-4-yl)methanone (7d)**

Synthesised according to general procedure E using compound **6a** (100 mg, 0.205 mmol) and (4-fluorophenyl)boronic acid (36 mg, 0.246 mmol) as reagents. Yield: 38 mg (39.0%); orange amorphous solid; ^1^H NMR (400 MHz, Chloroform-*d*) δ 7.97–7.87 (m, 2H, 2 × Ar-H), 7.70–7.59 (m, 7H, 7 × Ar-H), 7.41–7.32 (m, 2H, 2 × Ar-H), 7.16 (t, *J* = 8.7 Hz, 2H, 2 × Ar-H), 6.55 (s, 2H, NH_2_), 3.94 (s, 2H, piperidine-CH_2_), 3.49 (s, 4H, 2 × piperidine-CH_2_), 2.81 (s, 3H, N-CH_3_); ^13^C NMR (101 MHz, Chloroform-*d*) δ 190.01, 164.09, 162.80, 162.07, 161.63, 150.05, 146.02, 143.08, 139.40, 136.23, 132.75, 131.65, 130.27, 130.14, 129.42, 128.93, 128.85, 128.50, 127.64, 126.92, 120.97, 115.96, 115.75, 115.04, 105.53, 52.52, 50.76, 42.84, 29.55; HRMS (ESI+) for C_30_H_24_FN_3_OS (M+H^+^): calculated 494.16785; measured 494.16969; HPLC: *t*_R_ = 5.79 min (95.1% at 254 nm).

**(3-Amino-6-methyl-4-phenyl-5,6,7,8-tetrahydrothieno [2,3-b][1,6]naphthyridin-2-yl)(4′-bromo-[1,1′-biphenyl]-4-yl)methanone (7e)**

Synthesised according to general procedure E using compound **6a** (100 mg, 0.205 mmol) and (4-bromophenyl)boronic acid (49 mg, 0.246 mmol) as reagents. Yield: 50 mg (43.1%); orange amorphous solid; ^1^H NMR (400 MHz, DMSO-*d*_6_) δ 7.78–7.61 (m, 7H, 7 × Ar-H), 7.50–7.43 (m, 2H, 2 × Ar-H), 3.15 (s, 2H, piperidine-CH_2_), 3.09 (d, *J* = 5.9 Hz, 2H, piperidine-CH_2_), 2.72 (t, *J* = 5.9 Hz, 2H, piperidine-CH_2_), 2.25 (s, 3H, N-CH_3_); ^13^C NMR (101 MHz, Chloroform-*d*) δ 189.78, 160.78, 157.88, 150.97, 144.91, 142.53, 140.10, 139.15, 134.28, 132.01, 129.55, 129.46, 128.84, 128.56, 127.87, 126.79, 124.71, 122.26, 120.05, 104.74, 55.42, 52.38, 45.98, 33.39; HRMS (ESI+) for C_30_H_24_BrN_3_OS (M+H^+^): calculated 554.08962; measured 554.08857; *t*_R_ = 6.14 min (97.4% at 254 nm).

**(3-Amino-6-methyl-4-phenyl-5,6,7,8-tetrahydrothieno [2,3-*b*][1,6]naphthyridin-2-yl)(4′-hydroxy-[1,1′-biphenyl]-4-yl)methanone (7f)**

Synthesised according to general procedure E using compound **6a** (100 mg, 0.205 mmol) and (4-hydroxyphenyl)boronic acid (33 mg, 0.246 mmol) as reagents. Yield: 38 mg (38.6%); orange amorphous solid; ^1^H NMR (400 MHz, Chloroform-*d*) δ 7.93–7.87 (m, 2H, 2 × Ar-H), 7.68–7.63 (m, 2H, 2 × Ar-H), 7.60 (dd, *J* = 5.2, 2.0 Hz, 3H, 3 × Ar-H), 7.35 (dd, *J* = 7.1, 2.5 Hz, 2H, 2 × Ar-H), 7.24 (d, *J* = 7.7 Hz, 1H, Ar-H), 7.04 (d, *J* = 7.7 Hz, 1H, Ar-H), 6.96 (t, *J* = 2.0 Hz, 1H, Ar-H), 6.72 (d, *J* = 7.7 Hz, 1H, Ar-H), 3.78 (s, 2H, NH_2_), 3.26 (d, *J* = 8.1 Hz, 4H, 2 × piperidine-CH_2_), 2.80 (t, *J* = 6.0 Hz, 2H, piperidine-CH_2_), 2.38 (s, 3H, N-CH_3_); ^13^C NMR (101 MHz, Chloroform-*d*) δ 190.07, 160.78, 157.82, 150.84, 146.85, 144.88, 144.05, 141.44, 139.71, 134.35, 129.82, 129.54, 129.44, 128.37, 127.90, 126.95, 124.73, 120.11, 117.75, 114.68, 113.90, 104.87, 55.49, 52.44, 46.05, 33.44; HRMS (ESI+) for C_30_H_25_N_3_O_2_S calculated 492.17245; measured 492.17402; HPLC: *t*_R_ = 4.87 min (95.0% at 254 nm).

**(3-Amino-6-methyl-4-phenyl-5,6,7,8-tetrahydrothieno [2,3-*b*][1,6]naphthyridin-2-yl)(3′-amino-[1,1′-biphenyl]-4-yl)methanone (7g)**

Synthesised according to general procedure E using compound **6a** (100 mg, 0.205 mmol) and (3-aminophenyl)boronic acid (42 mg, 0.246 mmol) as reagents. Yield: 60 mg (61.2%); orange amorphous solid; ^1^H NMR (400 MHz, Chloroform-*d*) δ 7.94–7.87 (m, 2H, 2 × Ar-H), 7.66–7.57 (m, 5H, 5 × Ar-H), 7.52 (d, *J* = 8.5 Hz, 2H, 2 × Ar-H), 7.37–7.34 (m, 2H, 2 × Ar-H), 6.92 (d, *J* = 8.6 Hz, 2H, 2 × Ar-H), 3.27 (d, *J* = 7.9 Hz, 4H, 2 × piperidine-CH_2_), 2.81 (t, *J* = 5.8 Hz, 2H, piperidine-CH_2_), 2.38 (s, 3H, N-CH_3_); HRMS (ESI+) for C_30_H_26_N_4_O_2_S calculated 491.18832; measured 491.19001; HPLC: *t*_R_ = 4.97 min (94.2% at 254 nm).

**[1,1′-Biphenyl]-4-yl(3-amino-6-methyl-4-phenyl-5,6,7,8-tetrahydrothieno [2,3-*b*][1,6]naphthyridin-2-yl)methanone (7h)**

Synthesised according to general procedure E using compound **6a** (30 mg, 0.063 mmol) and phenylboronic acid (9 mg, 0.075 mmol) as reagents. Yield: 11 mg (36.7%); orange amorphous solid; ^1^H NMR (400 MHz, Chloroform-*d*) δ 7.96–7.86 (m, 2H, 2 × Ar-H), 7.76–7.56 (m, 7H, 7 × Ar-H), 7.52–7.35 (m, 4H, 4 × Ar-H), 3.87 (s, 2H, piperidine-CH_2_), 3.67 (s, 2H, piperidine-CH_2_), 3.37 (s, 2H, piperidine-CH_2_),2.89 (s, 3H, N-CH_3_); ^13^C NMR (101 MHz, Chloroform-d) δ 190.20, 162.30, 152.49, 149.88, 146.19, 144.20, 140.09, 139.33, 132.55, 130.38, 128.93, 128.45, 128.02, 127.27, 127.11, 121.14, 117.57, 105.75, 77.23, 52.31, 50.72, 42.52, 29.03; HRMS (ESI+) for C_30_H_25_N_3_OS calculated 476.17766; measured 476.17911; HPLC: *t*_R_ = 5.51 min (96.8% at 254 nm).

***tert*-Butyl-3-amino-2-(4′-bromo-[1,1′-biphenyl]-4-carbonyl)-4-phenyl-7,8-dihydrothieno [2,3-*b*][1,6]naphthyridine-6(5*H*)-carboxylate (7i)**

Synthesised according to general procedure E using compound **6b** (150mg, 0.266 mmol) and (4-bromophenyl)boronic acid (64 mg, 0.319 mmol) as reagents. Crude product was used in the next step.

***tert*-Butyl-3-amino-2-(4′-chloro-[1,1′-biphenyl]-4-carbonyl)-4-phenyl-7,8-dihydrothieno [2,3-*b*][1,6]naphthyridine-6(5*H*)-carboxylate (7j)**

Synthesised according to general procedure E using compound **6b** (150 mg, 0.266 mmol) and (4-chlorophenyl)boronic acid (50 mg, 0.319 mmol) as reagents. Crude product was used in the next step.

**(3-Amino-4-(4-methoxyphenyl)-6-methyl-5,6,7,8-tetrahydrothieno [2,3-*b*][1,6]naphthyridin-2-yl)(4′-chloro-[1,1′-biphenyl]-4-yl)methanone (7k)**

Synthesised according to general procedure E using compound **6c** (150 mg, 0.295 mmol) and (4-chlorophenyl)boronic acid (55 mg, 0.355 mmol) as reagents. Yield: 25 mg (15.6%); orange amorphous solid; ^1^H NMR (400 MHz, Chloroform-*d*) δ 7.95–7.89 (m, 2H, 2 × Ar-H), 7.65 (d, *J* = 8.3 Hz, 2H, 2 × Ar-H), 7.58 (d, *J* = 8.6 Hz, 2H, 2 × Ar-H), 7.44 (d, *J* = 8.5 Hz, 2H, 2 × Ar-H), 7.25 (s, 2H, 2 × Ar-H), 7.11 (d, *J* = 8.8 Hz, 2H, 2 × Ar-H), 3.93 (s, 3H, O-CH_3_), 3.28 (s, 2H, piperidine-CH_2_), 3.24 (t, *J* = 6.0 Hz, 2H, piperidine-CH_2_), 2.80 (t, *J* = 6.0 Hz, 2H, piperidine-CH_2_), 2.39 (s, 3H, N-CH_3_); HRMS (ESI+) for C_31_H_26_ClN_3_O_2_S (M+H^+^): calculated 540.15070; measured 540.14971; HPLC: *t*_R_ = 5.85 min (94.6% at 254 nm).

**(3-Amino-4-phenyl-5,6,7,8-tetrahydrothieno [2,3-*b*][1,6]naphthyridin-2-yl)(4′-chloro-[1,1′-biphenyl]-4-yl)methanone (7l)**

Synthesised according to general procedure F using compound **7j** as a reagent. Yield: 32 mg (24.5%); orange amorphous solid; ^1^H NMR (400 MHz, Chloroform-*d*) δ 7.97–7.90 (m, 2H, 2 × Ar-H), 7.67–7.63 (m, 2H, 2 × Ar-H), 7.62–7.56 (m, 5H, 5 × Ar-H), 7.52 (d, *J* = 8.5 Hz, 2H, 2 × Ar-H), 7.34 (dd, *J* = 7.4, 2.2 Hz, 2H, 2 × Ar-H), 3.72 (s, 2H, piperidine-CH_2_), 3.23 (d, *J* = 5.7 Hz, 2H, piperidine-CH_2_), 3.15 (t, *J* = 5.8 Hz, 2H, piperidine-CH_2_); HRMS (ESI+) for C_29_H_22_BrN_3_OS (M+H^+^): calculated 496.12449; measured 496.12352.

**(3-Amino-4-phenyl-5,6,7,8-tetrahydrothieno [2,3-*b*][1,6]naphthyridin-2-yl)(4′-bromo-[1,1′-biphenyl]-4-yl)methanone (7m)**

Synthesised according to general procedure F using compound **7i** as a reagent. Yield: 20 mg (12.4%); orange amorphous solid; ^1^H NMR (400 MHz, Chloroform-*d*) δ 7.95–7.89 (m, 2H, 2 × Ar-H), 7.69–7.63 (m, 2H, 2 × Ar-H), 7.63–7.54 (m, 5H, 5 × Ar-H), 7.48–7.41 (m, 2H, 2 × Ar-H), 7.38–7.32 (m, 2H, 2 × Ar-H), 3.72 (s, 2H, piperidine-CH_2_), 3.24 (t, *J* = 5.9 Hz, 2H, piperidine-CH_2_), 3.15 (t, *J* = 5.9 Hz, 2H, piperidine-CH_2_); HRMS (ESI+) for C_29_H_22_ClN_3_OS (M+H^+^): calculated 540.07397; measured 540.07316.
