# Supplementary material for: In Silico Discovery and Optimisation of a Novel Structural Class of Hsp90 C-Terminal Domain Inhibitors
Source: Biomolecules. 2022 Jun 24;12(7):884. doi: 10.3390/biom12070884 (PMC9312846; doi:10.3390/biom12070884)
Supplement: Supplementary file 1 [file biomolecules-12-00884-s001.zip › biomolecules-1762543-supplementary.pdf]

In Silico Discovery and Optimisation of a Novel Structural Class of Hsp90 C-Terminal Domain Inhibitors

Živa Zajec, Jaka Dernovšek, Martina Gobec and Tihomir Tomašič *

Representative ^{1}H NMR, ^{13}C NMR spectra and HPLC chromatograms

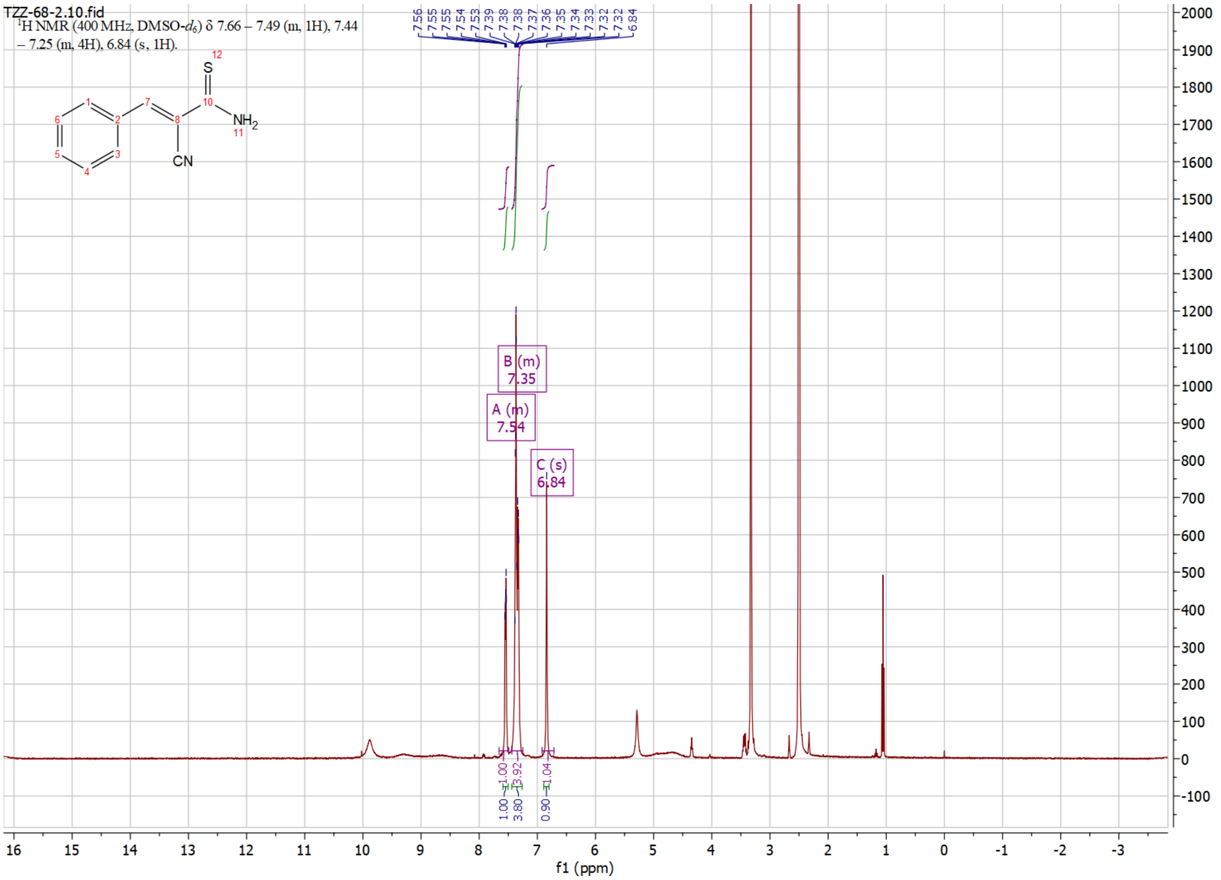

Figure S1: ^{1}H NMR spectrum of compound **3a**

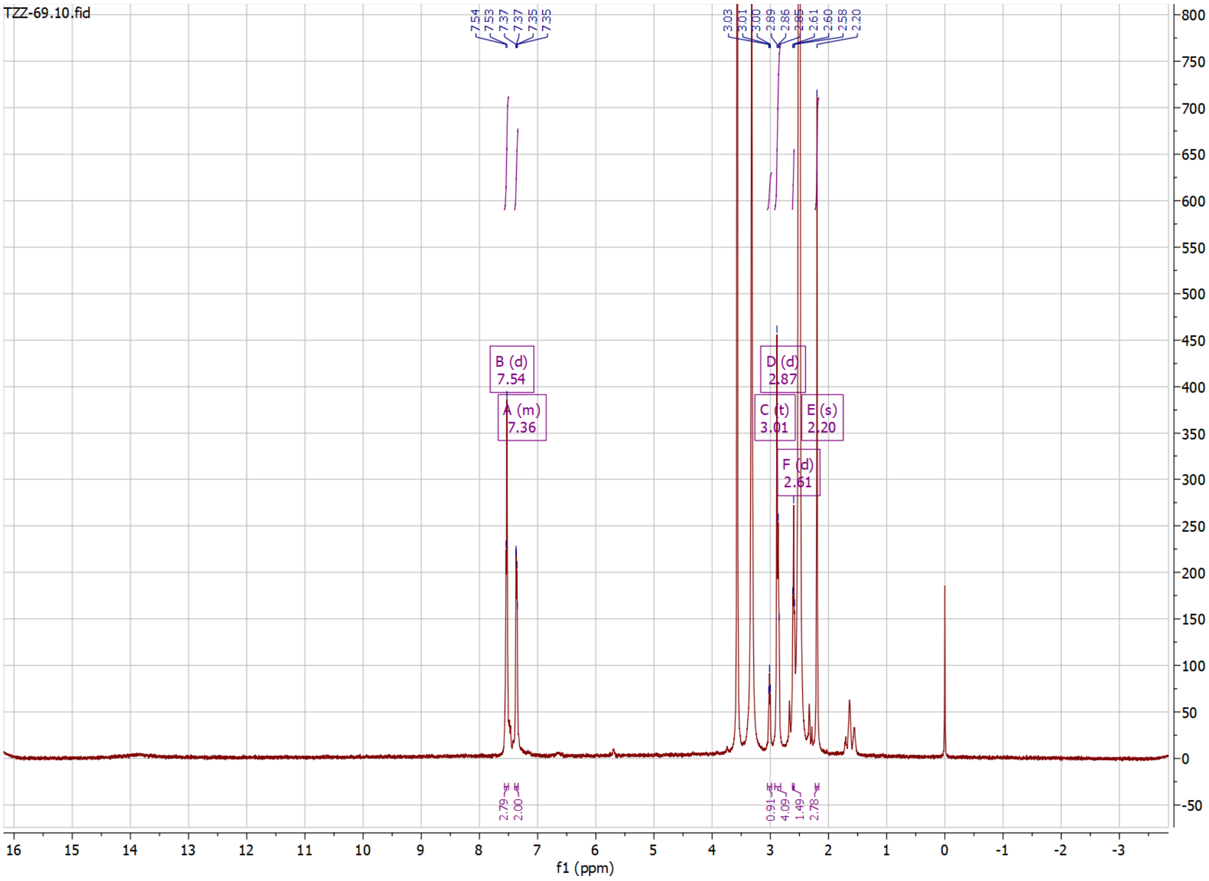

Figure S2: ^{1}H NMR spectrum of compound **4a**

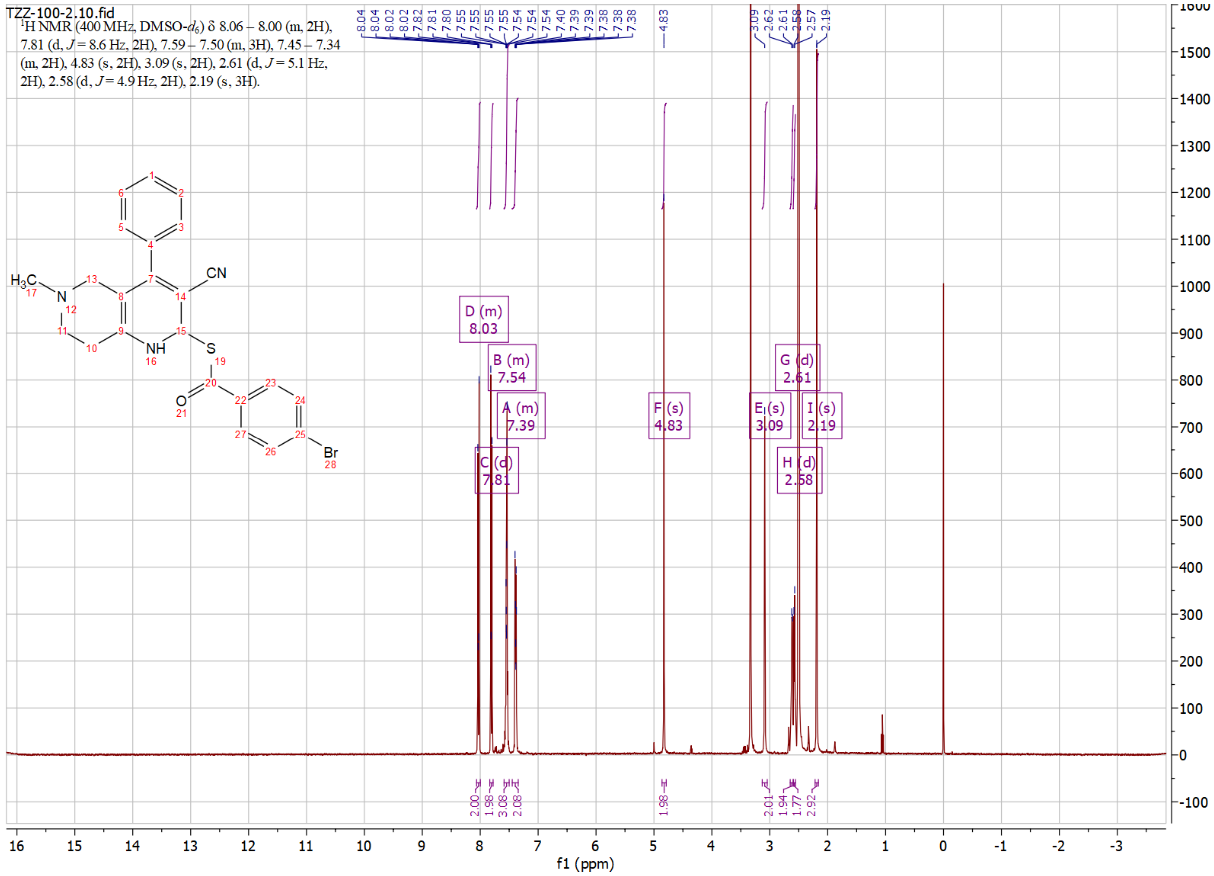

Figure S3: ^{1}H NMR spectrum of compound **5a**

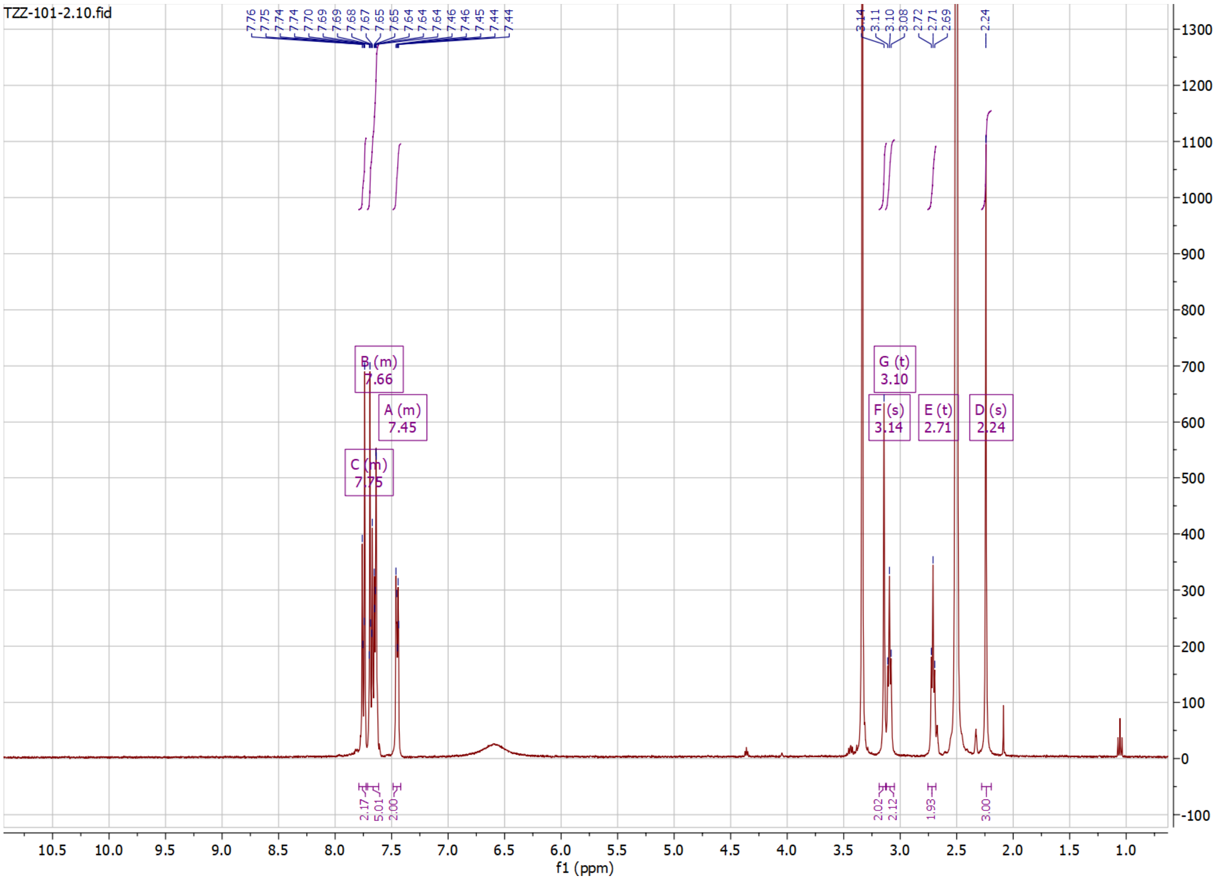

Figure S4: ^{1}H NMR spectrum of compound **6a**

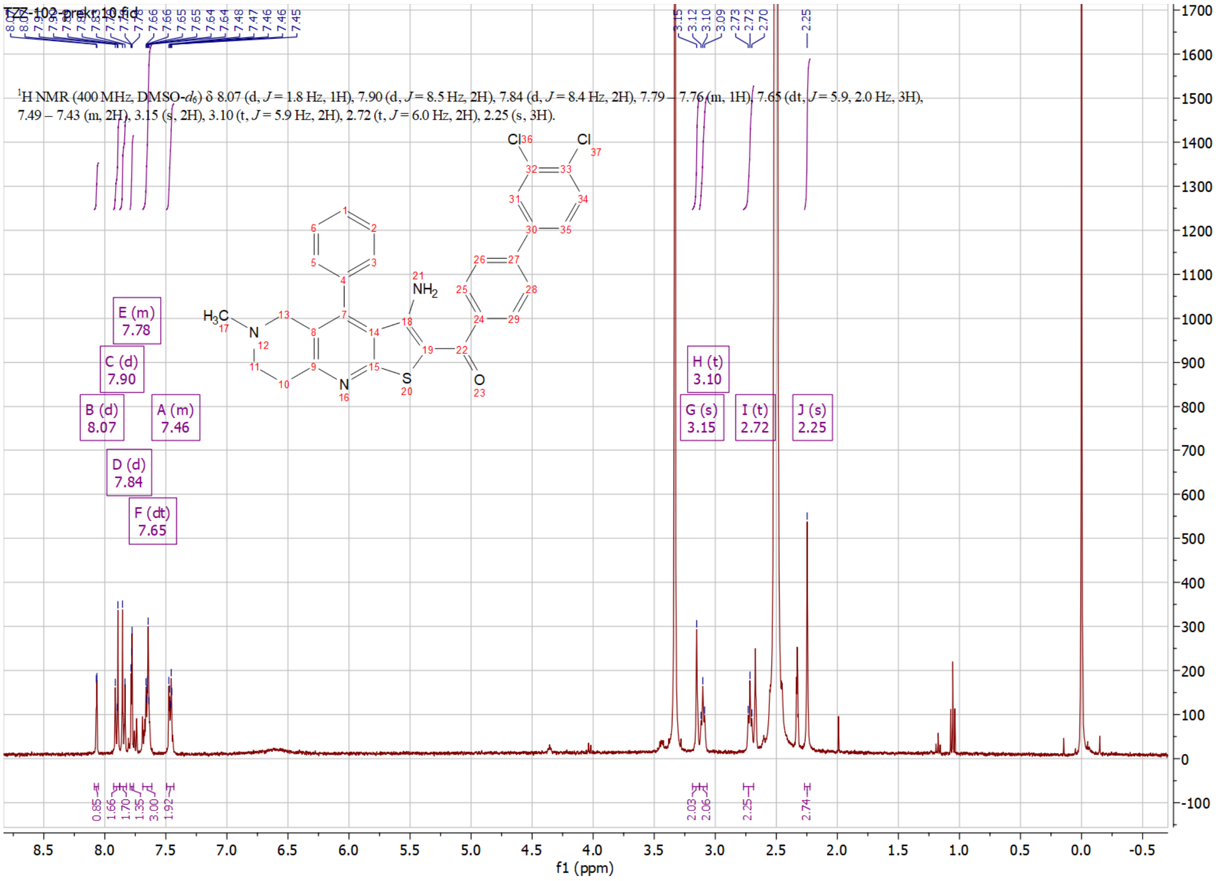

Figure S5: ^{1}H NMR spectrum of compound **7a**

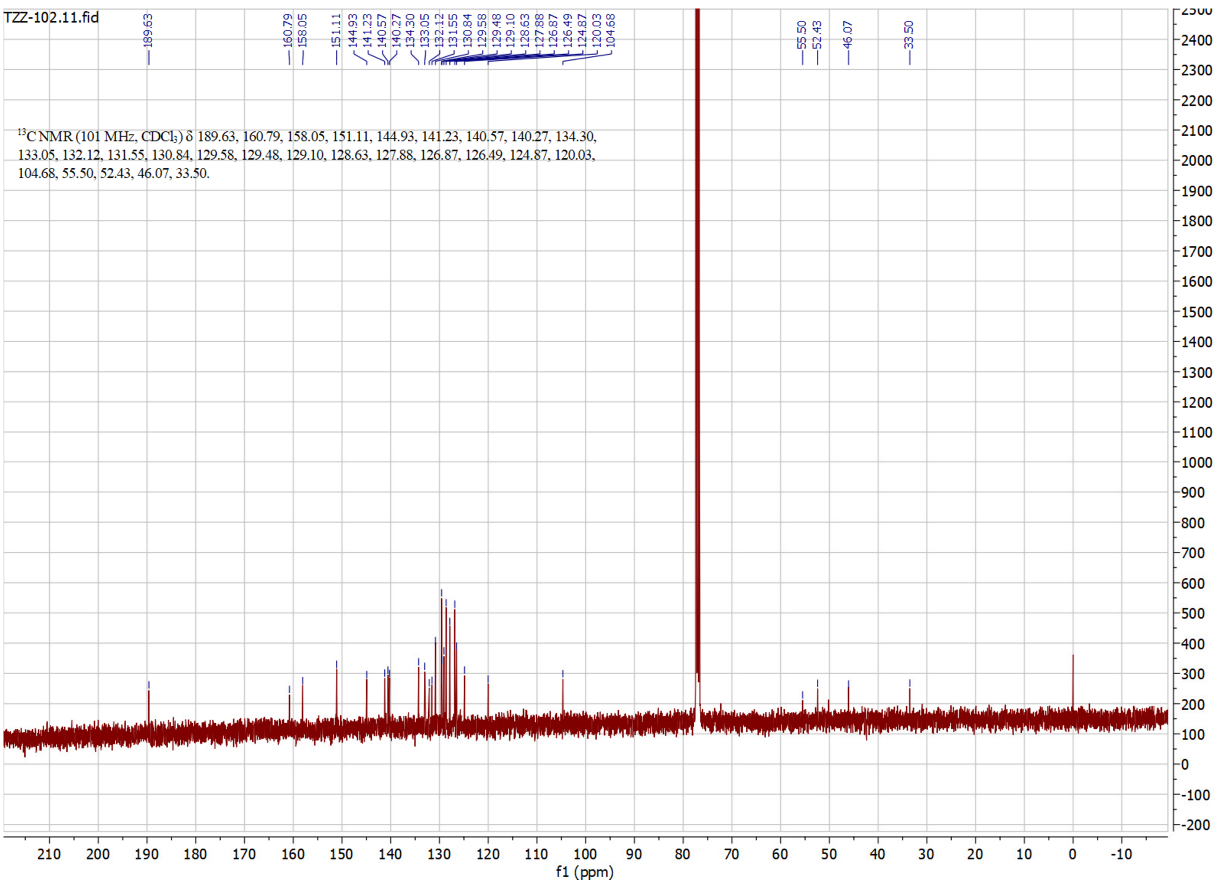

Figure S6: ^{13}C NMR spectrum of compound **7a**

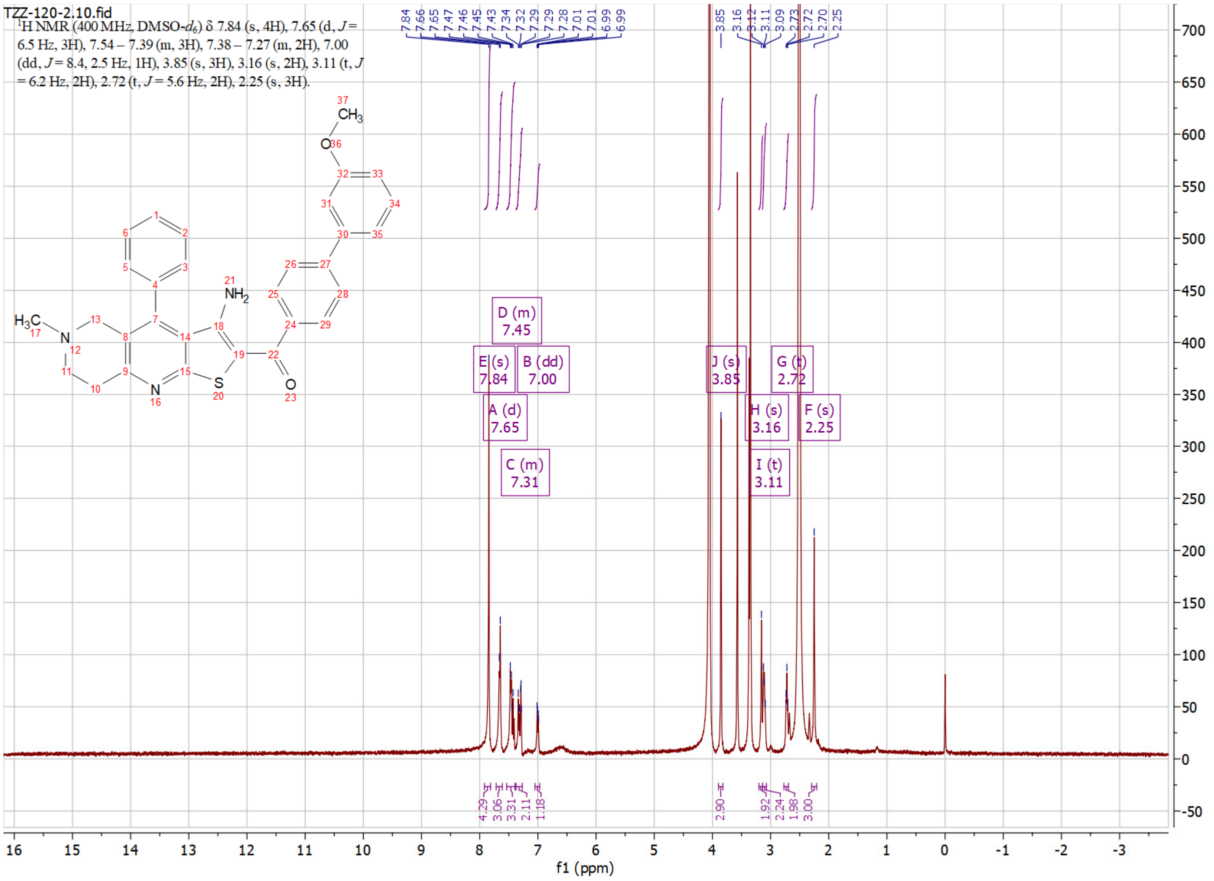

Figure S7: 1H NMR spectrum of compound **7b**

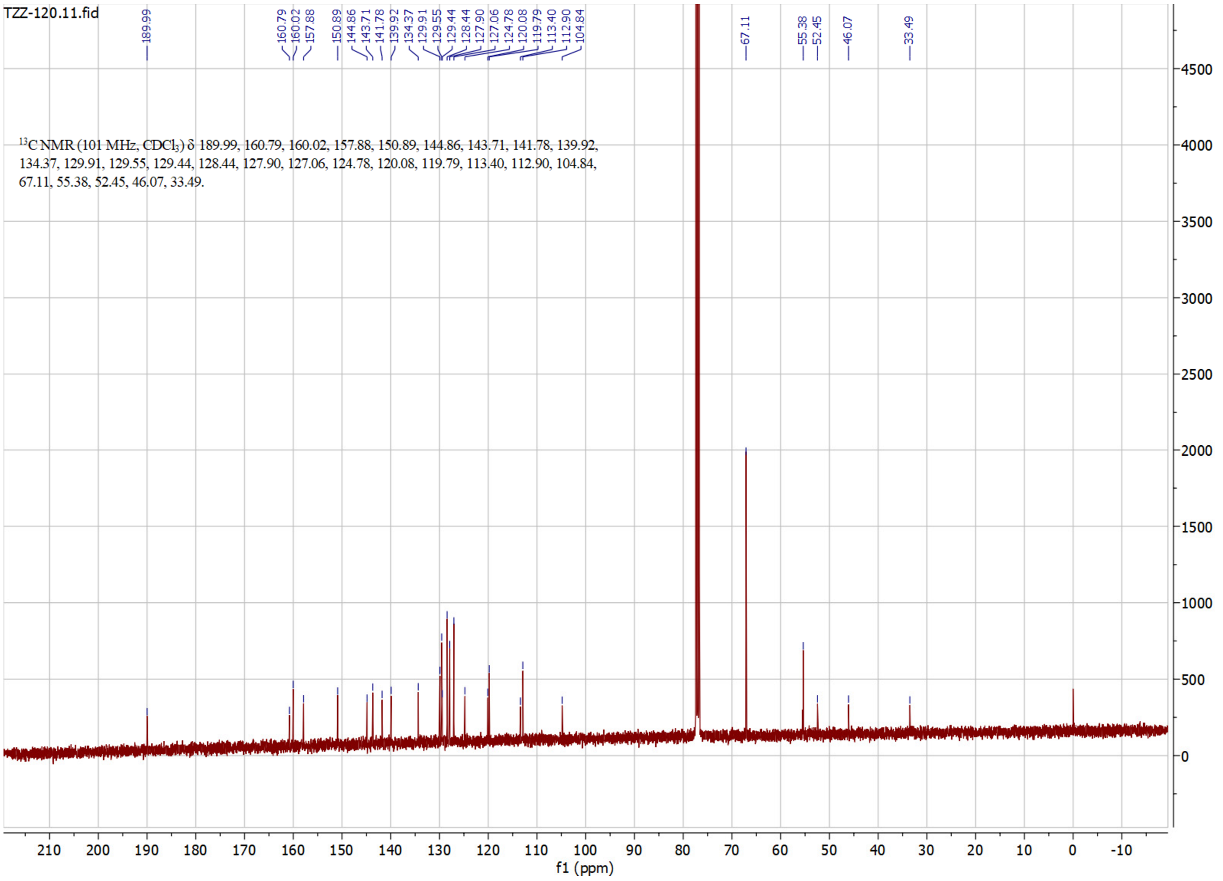

Figure S8: ^{13}C NMR spectrum of compound **7b**

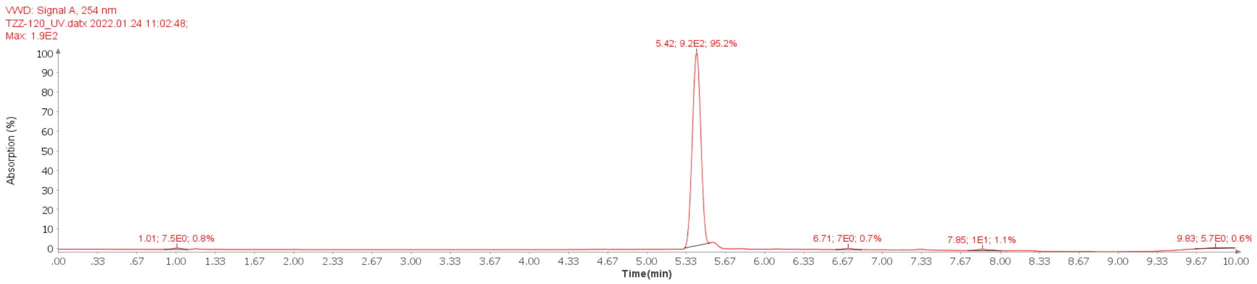

Figure S9: HPLC spectrum of **7b**

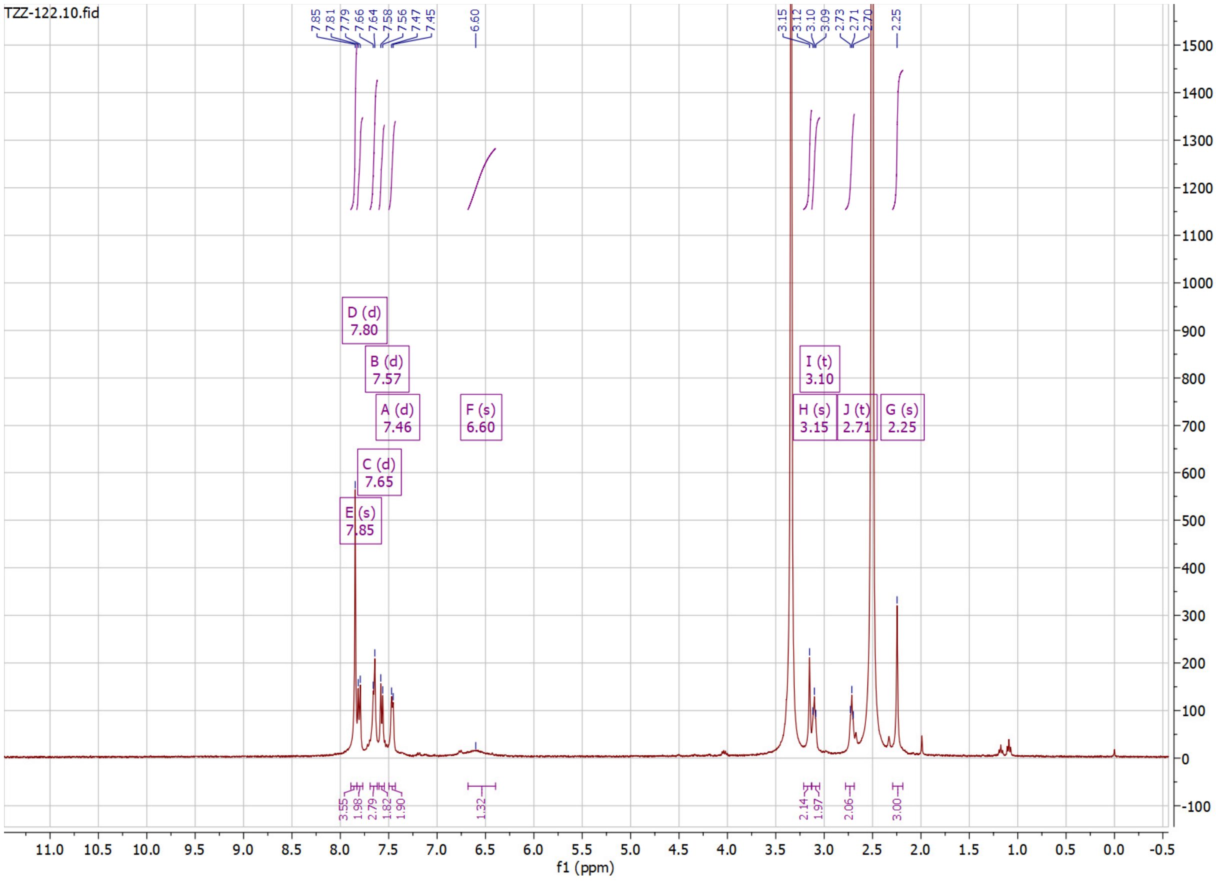

Figure S10: ^{1}H NMR spectrum of compound **7c**

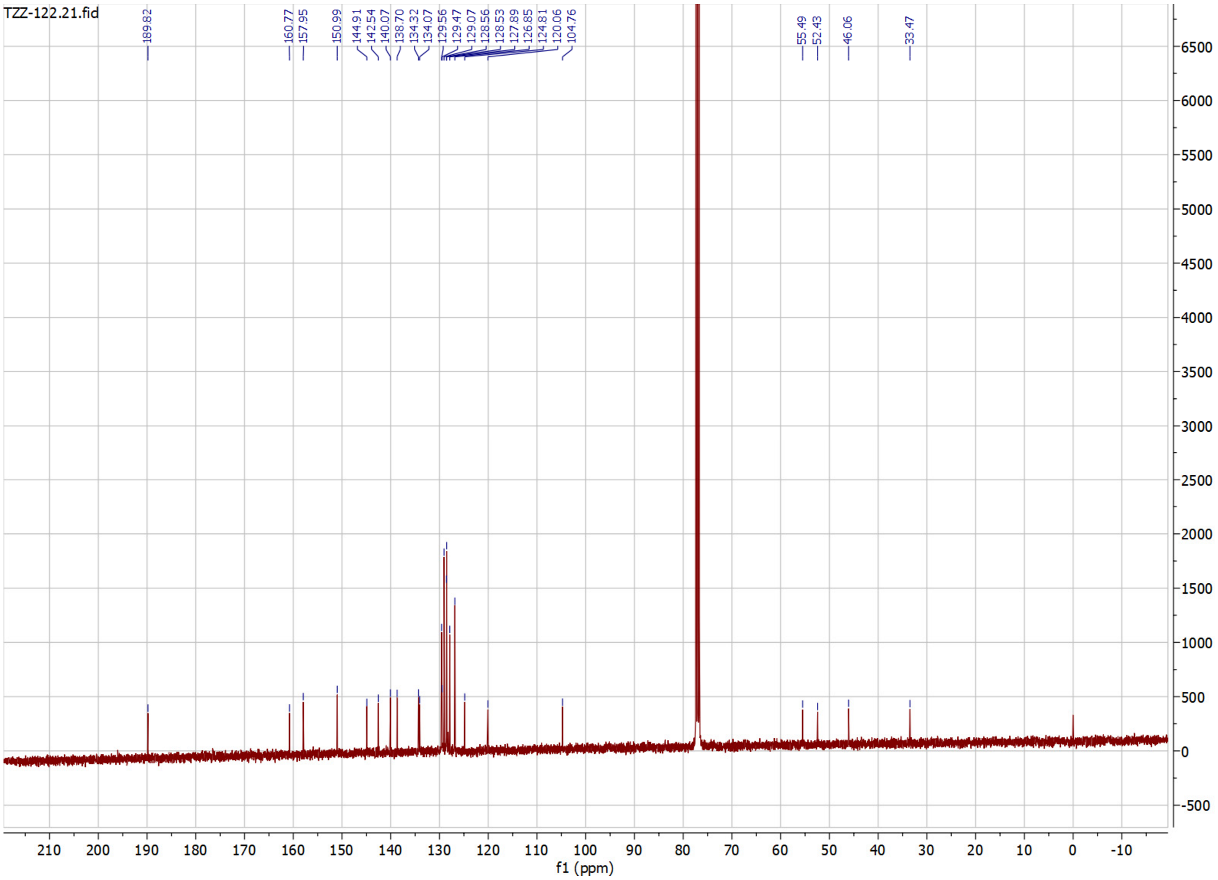

Figure S11: ^{13}C NMR spectrum of compound **7c**

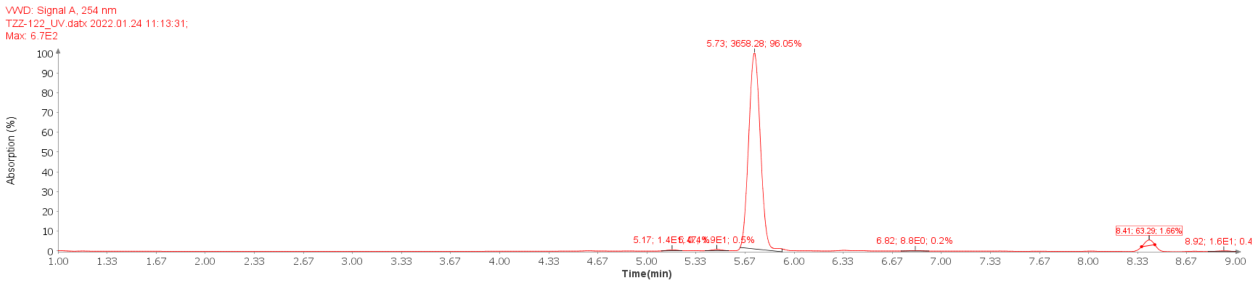

Figure S12: HPLC spectrum of compound **7c**

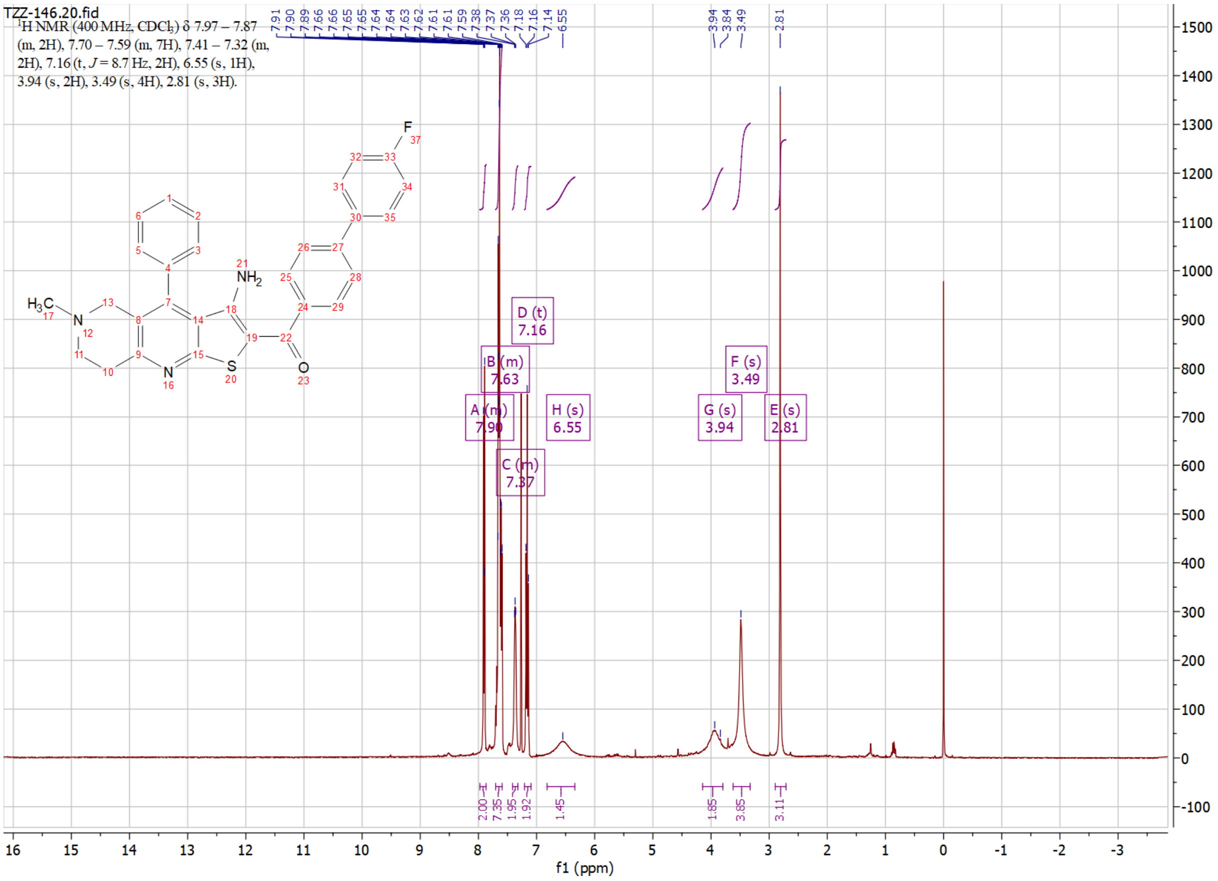

Figure S13: ¹H NMR spectrum of compound **7d**

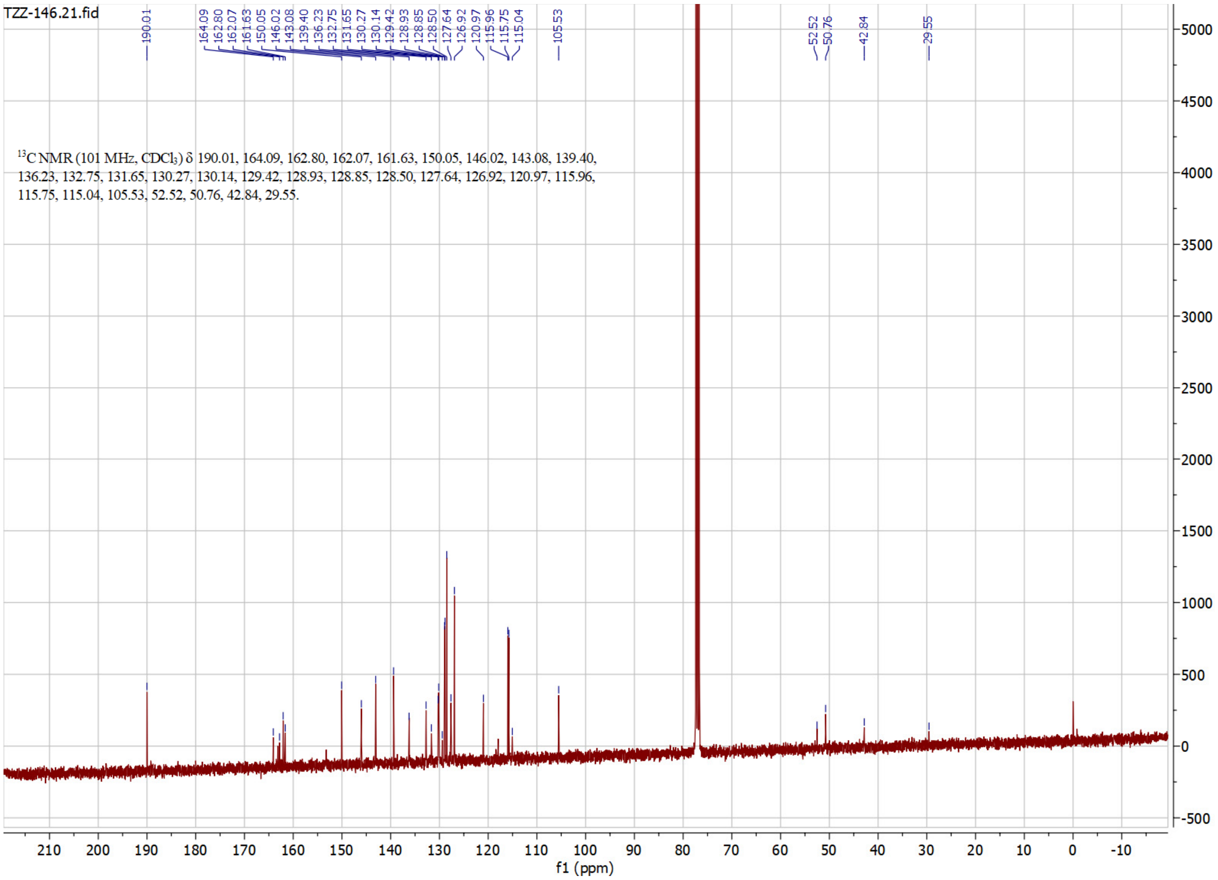

Figure S14:^{13}C NMR spectrum of compound **7d**

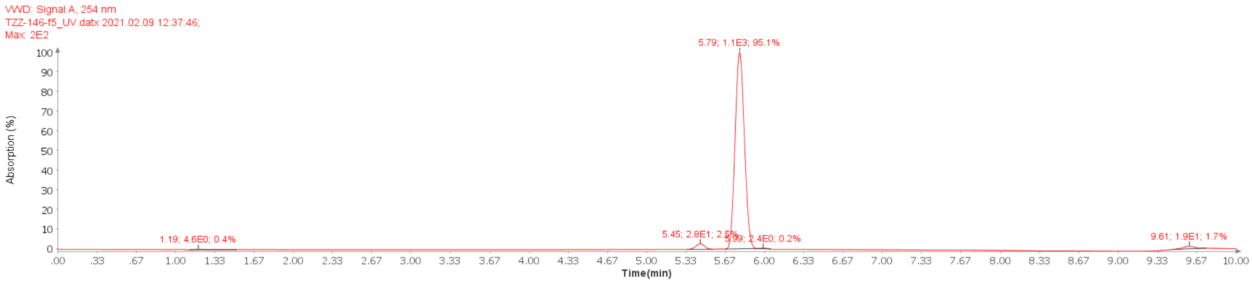

Figure S15: HPLC spectrum of compound **7d**

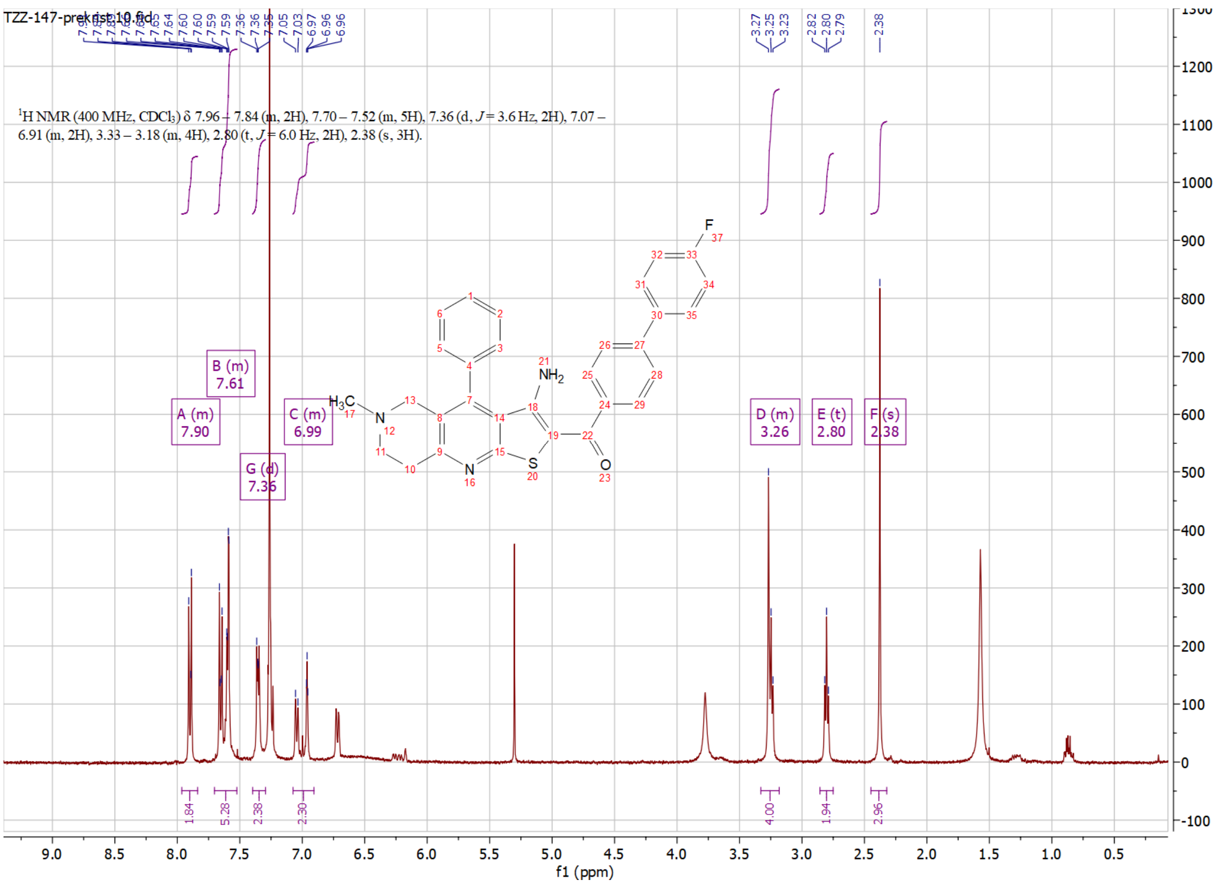

Figure S16: ^{1}H NMR spectrum of compound **7f**

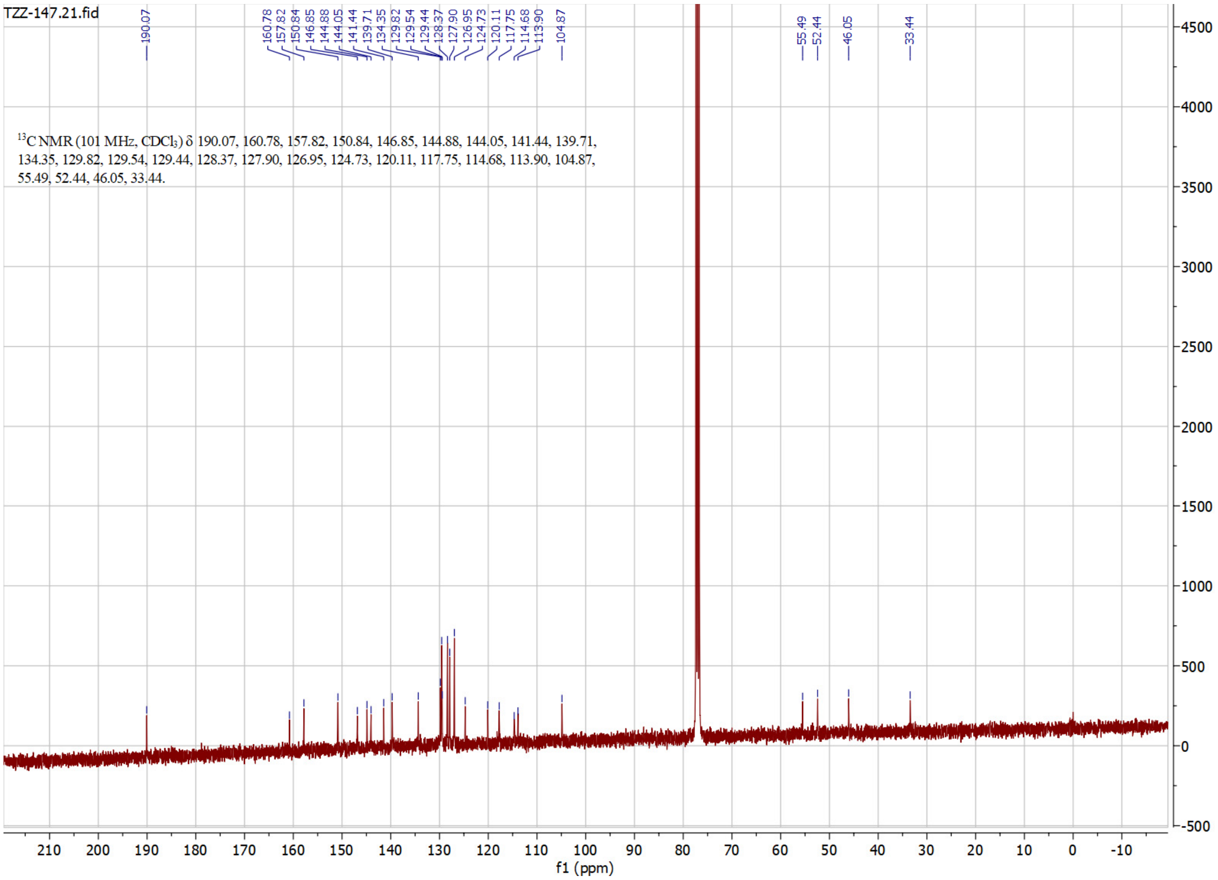

Figure S17: ^{13}C NMR spectrum of compound **7f**

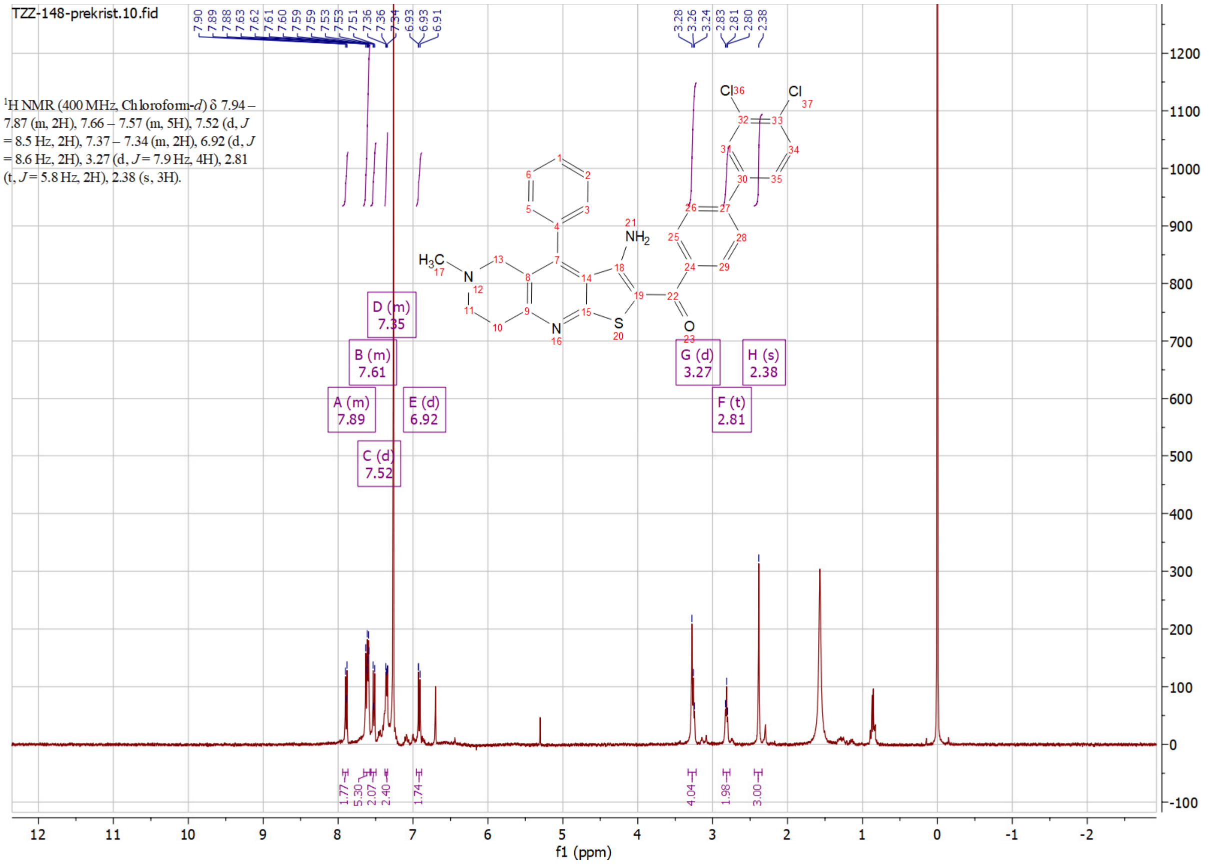

Figure S18: ^{1}H NMR spectrum of compound **7g**

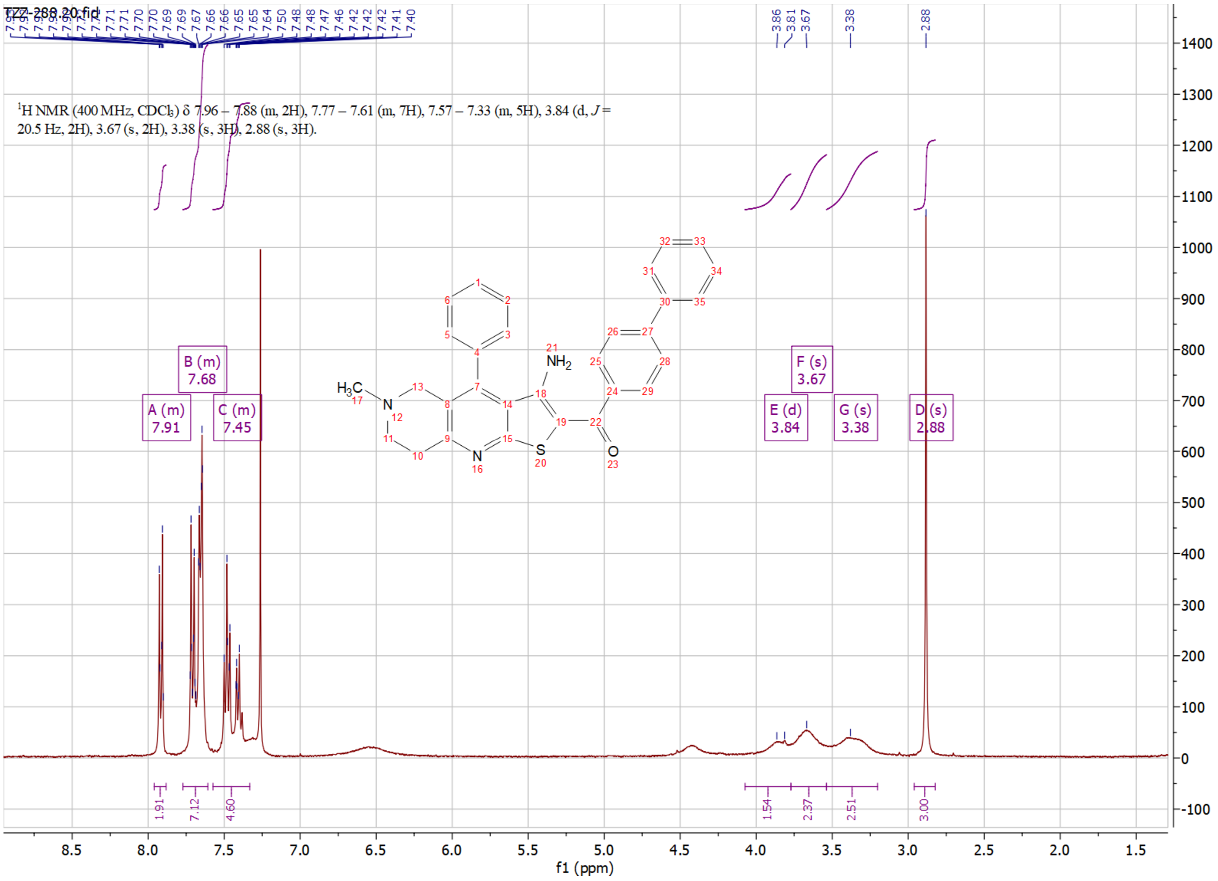

Figure S19: ^{1}H NMR spectrum of compound **7h**

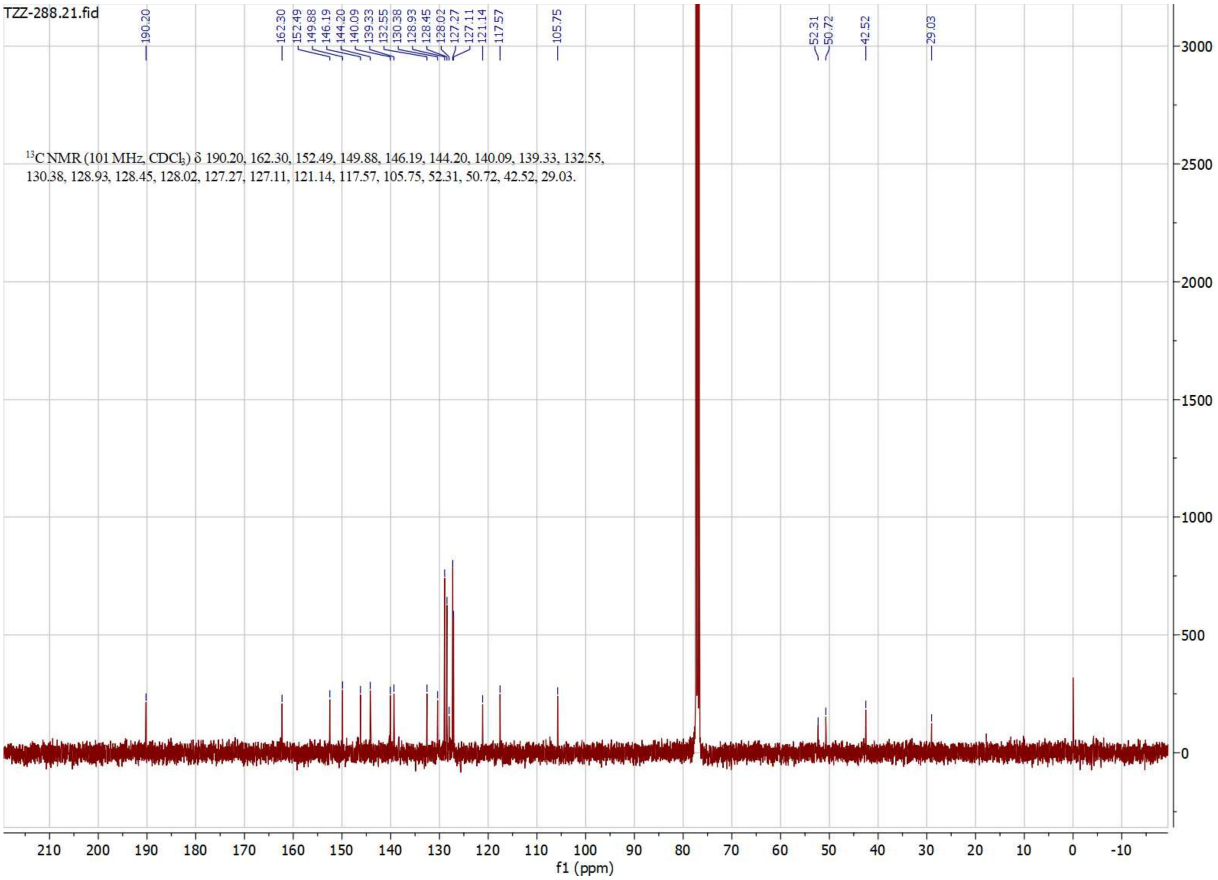

Figure S20: ^{13}C NMR spectrum of compound **7h**

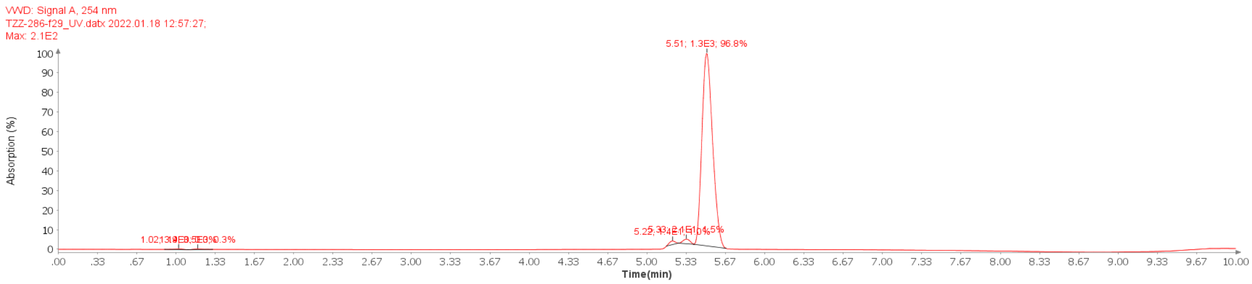

Figure S21: HPLC spectrum of compound **7h**

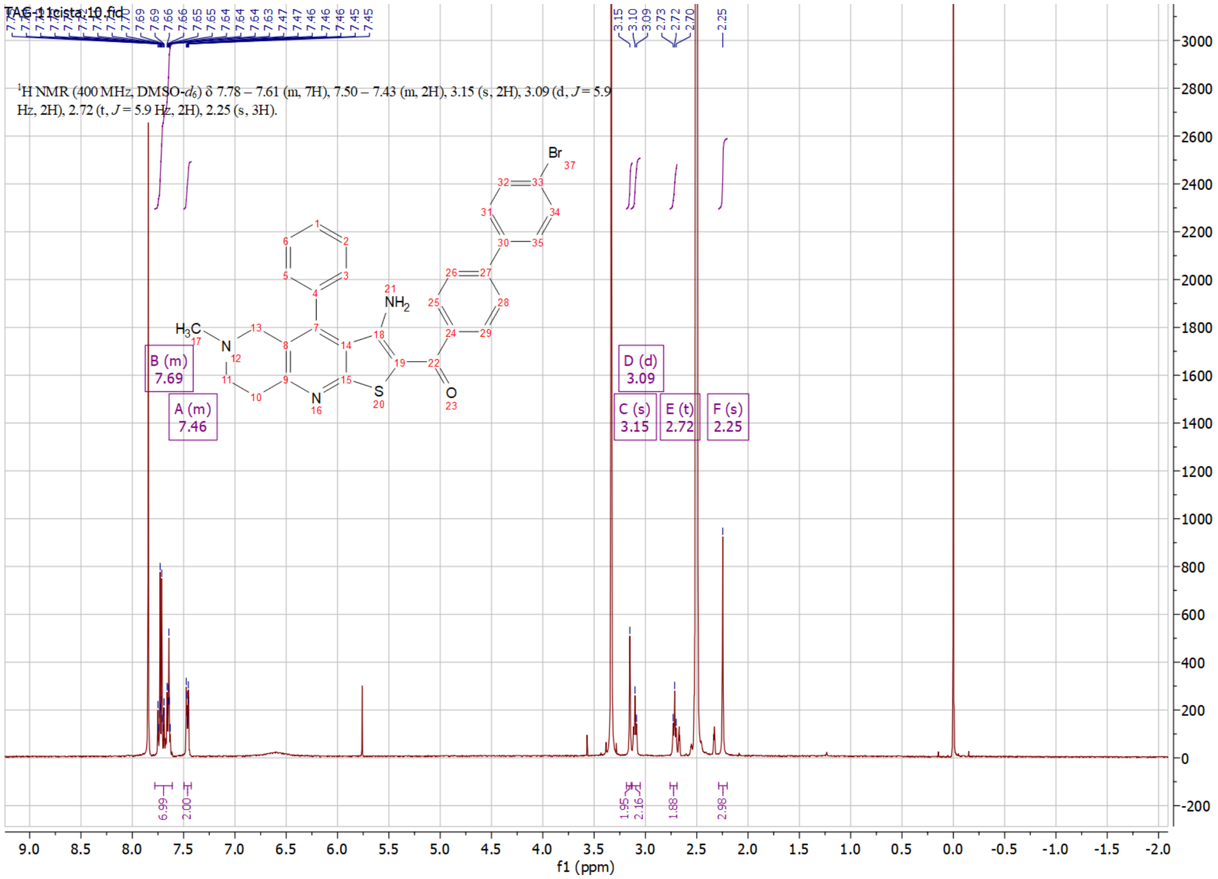

Figure S22: ^{1}H NMR spectrum of compound **7e**

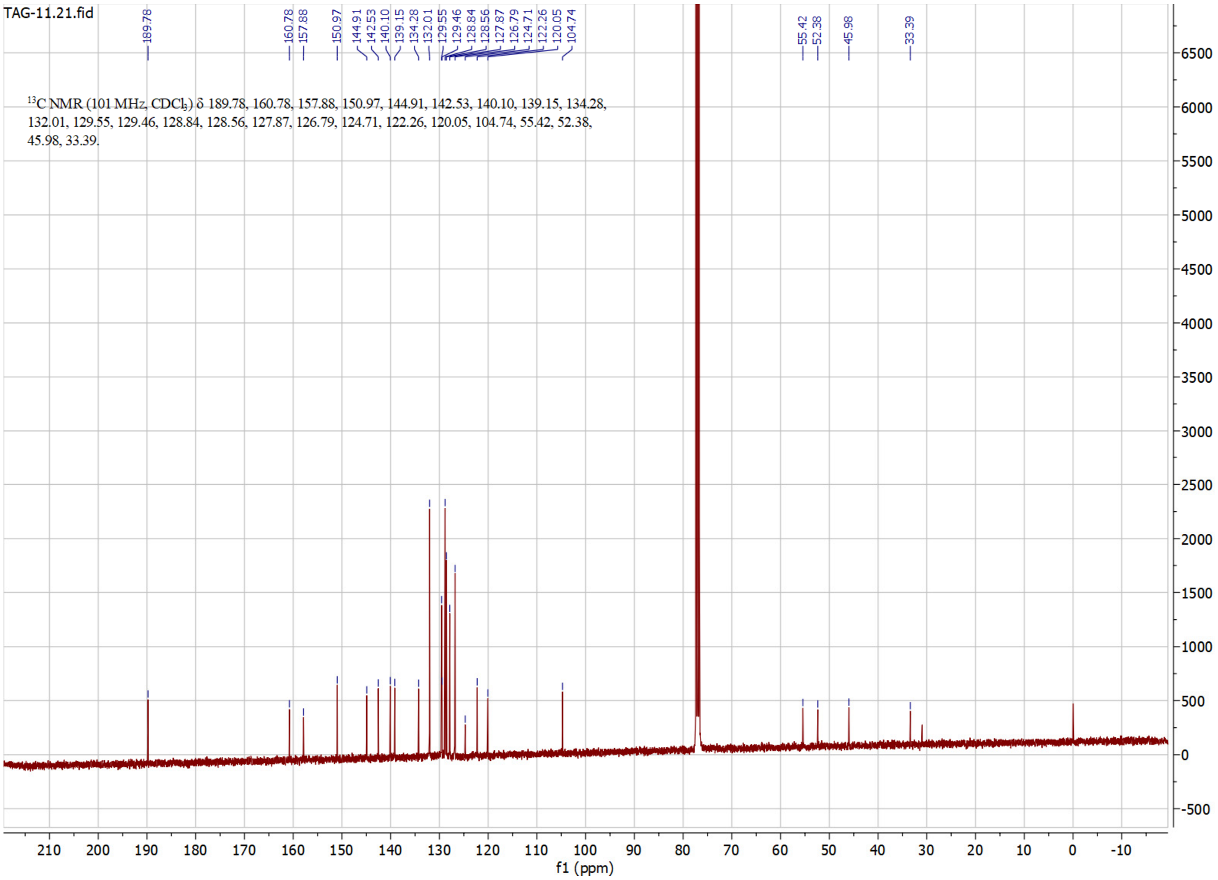

Figure S23: ^{13}C NMR spectrum of compound **7e**

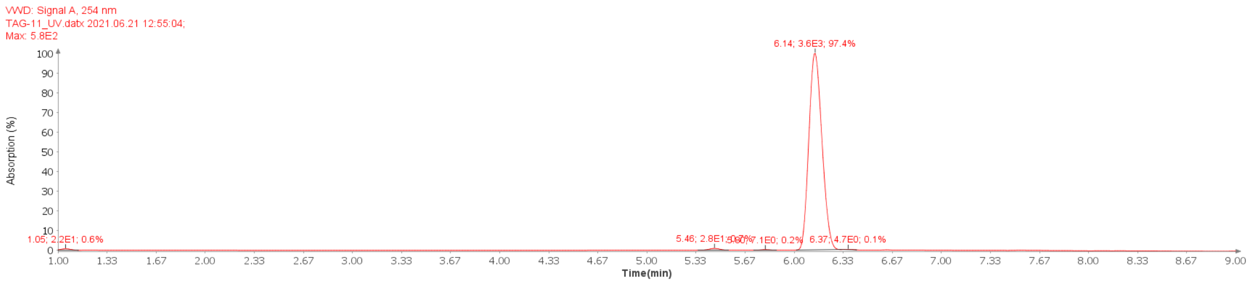

Figure S24: HPLC spectrum of compound **7e**

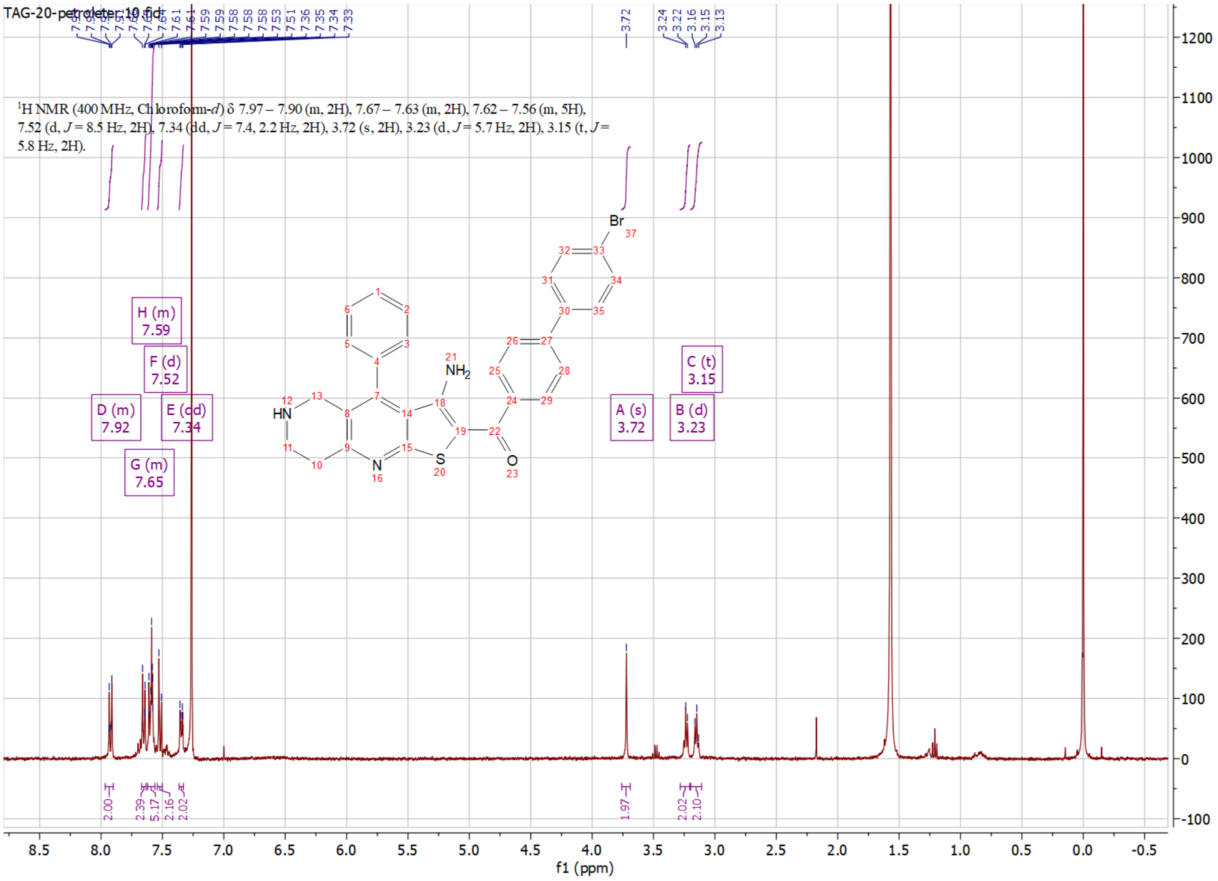

Figure S25: ^{1}H NMR spectrum of compound **7l**

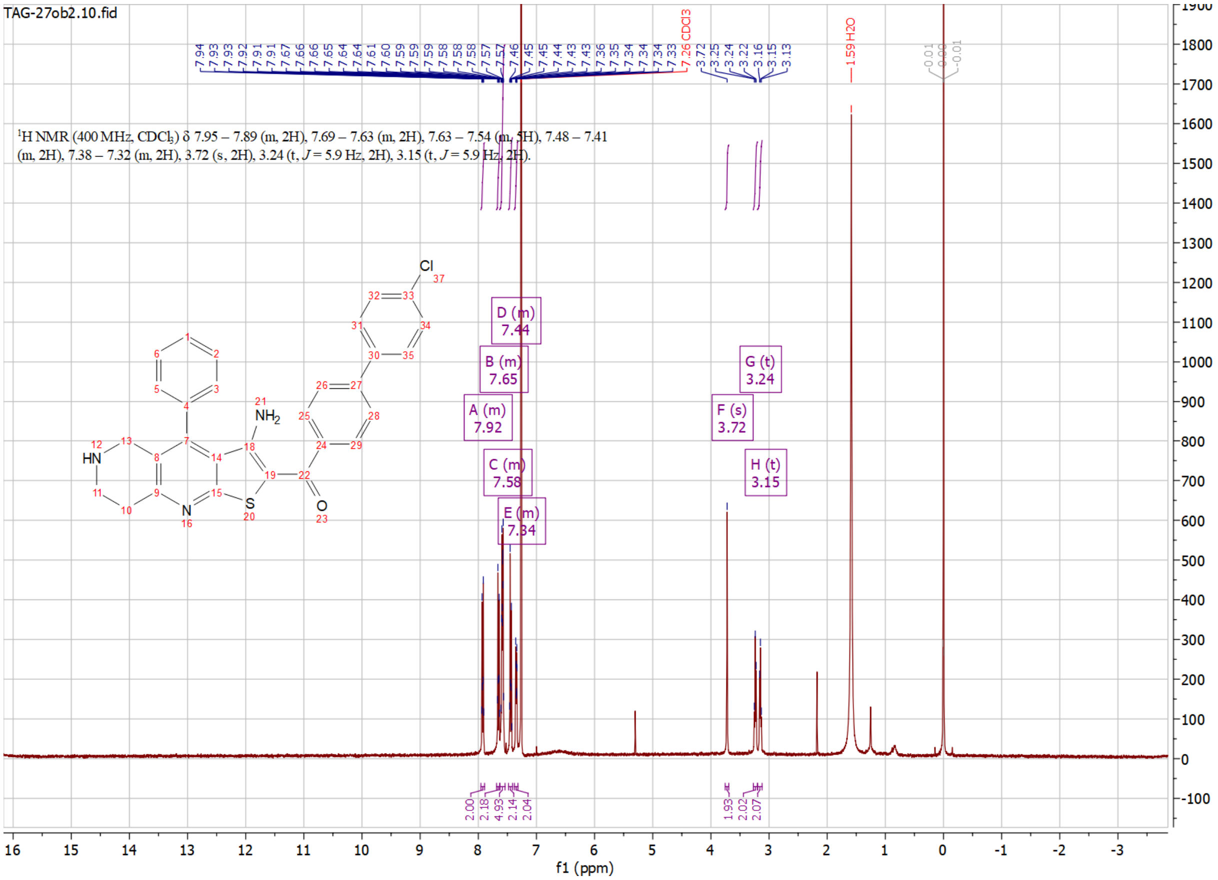

Figure S26: ^{1}H NMR spectrum of compound **7m**

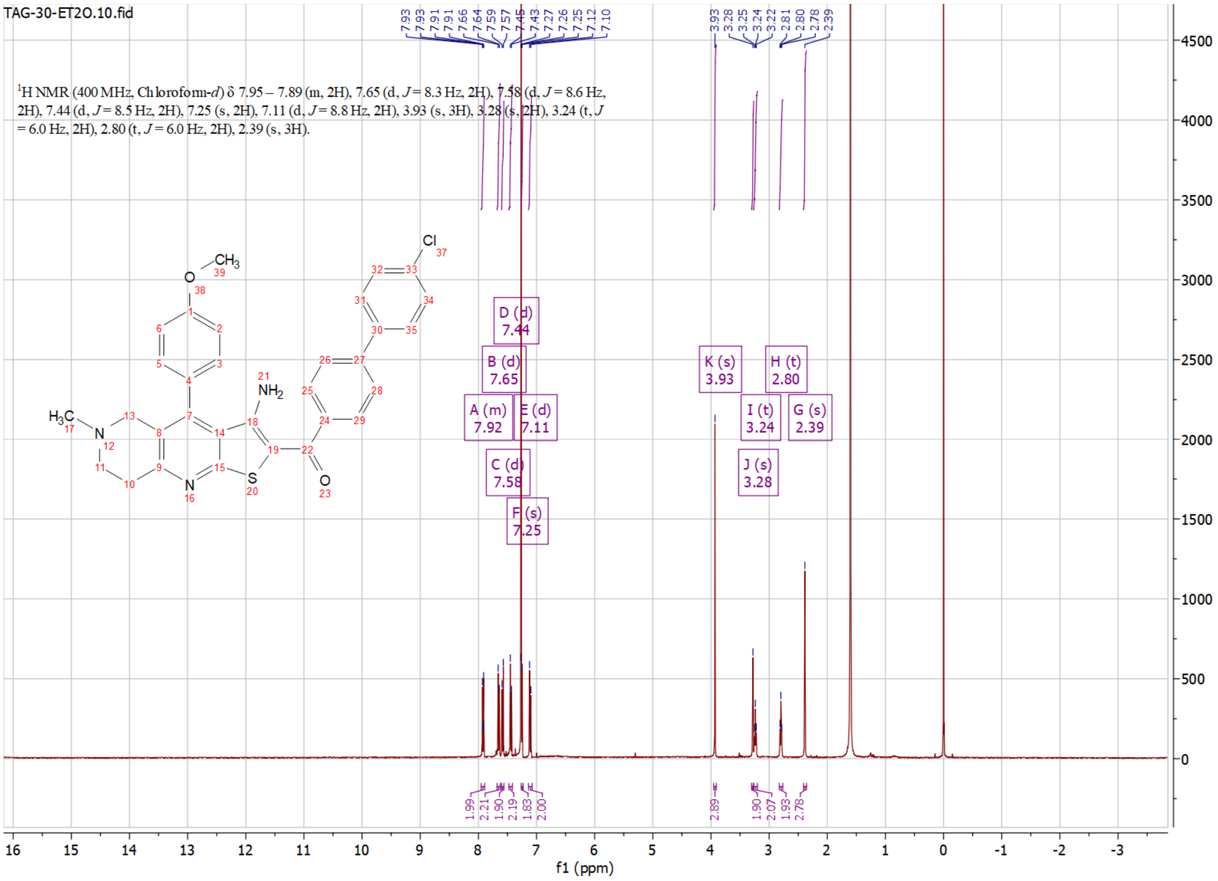

Figure S27: ^{1}H NMR spectrum of compound **7k**

Biological evaluation

Cell culture

The MCF-7 cells were cultured in Dubecco's modified Eagle's MEM medium (Sigma – Aldrich, St. Louis, MO, USA), which was supplemented with 10% fetal bovine serum (Gibco, Thermo Fisher Scientific, Waltham, MA, USA), 100 U/mL penicillin (Sigma-Aldrich, St. Louis, MO, USA), 100 µg/mL streptomycin (Sigma-Aldrich, St. Louis, MO, USA), 2 mM L-glutamine (Sigma-Aldrich, St. Louis, MO, USA). Cells were plated on 96-well plates at densitiy 4000 cells/well and were alowed to attach. Cells were treated with final compounds (6 concentrations, 3-fold dilution), positive control 17-DMAG or vehicle control (0.5% DMSO) and incubated for 72 hours.

SK-NM-C cells were cultured in RPMI 1640, HEPES modification medium (Sigma – Aldrich, St. Louis, MO, USA), supplemented with which was supplemented with 10% fetal bovine serum (Gibco, Thermo Fisher Scientific, Waltham, MA, USA), 100 U/mL penicillin (Sigma-Aldrich, St. Louis, MO, USA), 100 µg/mL streptomycin (Sigma-Aldrich, St. Louis, MO, USA), 2 mM L-glutamine (Sigma-Aldrich, St. Louis, MO, USA). Cells were plated on 96-well plates at densitiy 2000 cells/well and were alowed to attach. Cells were treated with final compounds (6 concentrations, 3-fold dilution), positive control 17-DMAG or vehicle control (0.5% DMSO) and incubated for 72 hours.

Representative IC_{50} curves

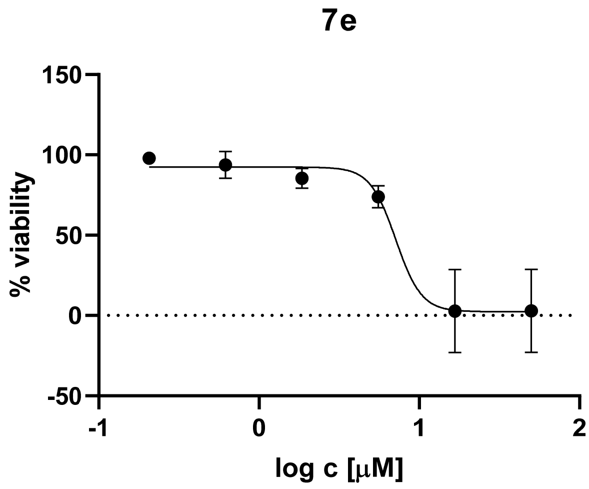

Figure S28: IC_{50} curve of compound **7e** in MCF-7 cell line

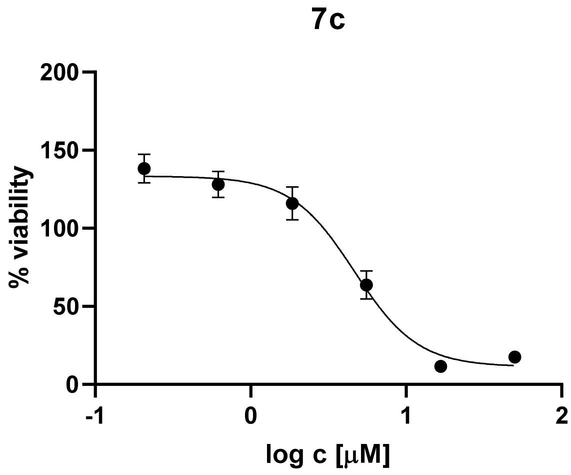

Figure S29: IC_{50} curve of compound **7c** in SK-NM-C cell line

Validation of chosen cell lines for expression of Hsp90

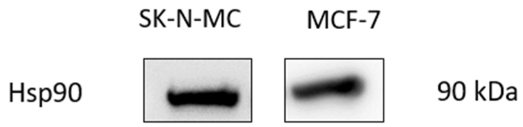

Figure S30: Western blot of validation of overexpression of Hsp90 in MCF-7 and SK-N-MC cell lines

*Luciferase refolding assay, screening of compound **7l***

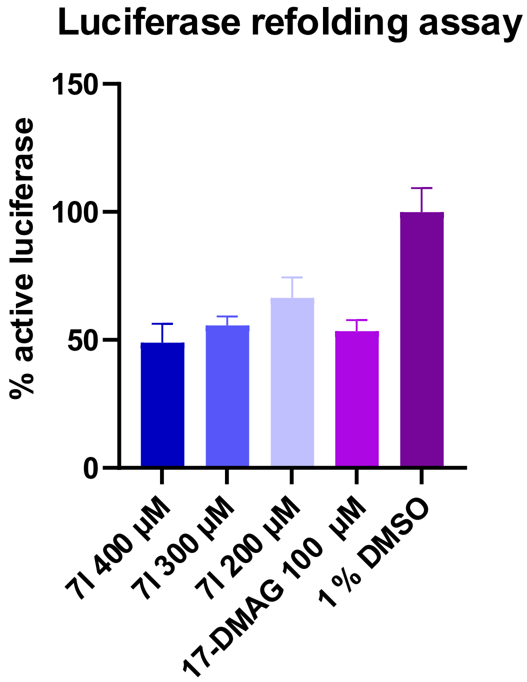

Figure S31: Percentage of active luciferase in luciferase refolding assay after treatment with compound **7l,** 17-DMAG (positive control) or 1 % DMSO (vehicle control)
